# Trends in cardiometabolic disease and health-related quality of life in the United States, 2001–2022

**DOI:** 10.1007/s11136-026-04300-1

**Published:** 2026-06-15

**Authors:** Danwei Yang, David D. Kim

**Affiliations:** 1 Department of Public Health Sciences, University of Chicago, Chicago, IL USA; 2 Department of Medicine, University of Chicago, Chicago, IL USA

**Keywords:** Health-related quality of life (HRQoL), EQ-5D, Cardiometabolic diseases, Temporal trends

## Abstract

**Purpose:**

To examine associations between cardiometabolic conditions and health-related quality of life (HRQoL) and evaluate temporal trends in condition-associated HRQoL decrements from 2001 to 2022.

**Methods:**

We analyzed nationally representative data from U.S. adults aged ≥ 18 years in the Medical Expenditure Panel Survey (2001–2022), excluding years without BMI data collection (2017, 2019, 2021). HRQoL was measured using EQ-5D utilities mapped from SF-12 scores with a validated algorithm. For each year, survey-weighted multivariable regression models estimated associations of sociodemographic characteristics, BMI, and six cardiometabolic conditions with HRQoL. Temporal trends in condition-associated HRQoL decrements were assessed using meta-regression. To estimate recent average associations, we pooled data from 2015, 2016, 2018, and 2022.

**Results:**

HRQoL improved over time, with lower values in 2001–2012 than 2013–2022 and an increase from its lowest value in 2012 (0.873) to highest in 2018 (0.888). Stroke contributed the greatest adjusted HRQoL decrement, followed by heart disease, diabetes, high blood pressure, obesity, and high cholesterol. Diabetes- and heart disease-associated decrements attenuated linearly over time (−0.0500 in 2001 to −0.0414 in 2022 and −0.0611 to −0.0487, respectively) , whereas high blood pressure-associated decrement was greatest around 2012 (-0.0361 in 2001, -0.0404 in 2012, and − 0.0313 in 2022) and obesity-associated decrement was smallest around 2012 (− 0.0311, − 0.0290, and − 0.0370).

**Conclusions:**

Changes in condition-associated HRQoL decrements over time suggest that utility values may not remain constant across calendar years. Smaller decrements for diabetes and heart disease may reflect better treatment and management, whereas the growing obesity-related decrement may indicate changes in the national severity of obesity. These patterns highlight the need for current, nationally representative utility estimates in population health research.

**Supplementary Information:**

The online version contains supplementary material available at https://doi.org/10.1007/s11136-026-04300-1.

## Abstract (plain language summary)

Cardiometabolic conditions such as diabetes, heart disease, stroke, high blood pressure, obesity, and high cholesterol are common chronic diseases in the United States and can reduce health-related quality of life. However, it is unclear whether their impact on health-related quality of life has changed over time as disease management and treatment have improved. Using nationally representative survey data from U.S. adults collected between 2001 and 2022, we estimated how much each cardiometabolic condition reduced health-related quality of life and whether the condition-specific estimates have changed over time. Overall health-related quality of life improved between 2001 and 2022, with the greatest gains among older adults. Stroke had the largest negative impact on health-related quality of life, followed by heart disease, diabetes, high blood pressure, obesity, and high cholesterol. The health-related quality-of-life gap between adults with and without diabetes or heart disease has narrowed over time, a pattern consistent with improved disease management and treatment. In contrast, the quality-of-life gap associated with obesity increased in recent years, a pattern consistent with the rise in severe obesity nationally. Researchers and policymakers may use up to date quality of life estimates rather than older figures when evaluating the burden of cardiometabolic disease. Using updated disease-attributable health-related quality of life estimates would better reflect the current burden of cardiometabolic disease.

**Supplementary Information:**

The online version contains supplementary material available at https://doi.org/10.1007/s11136-026-04300-1.

## Introduction

Health-related quality of life (HRQoL) reflects how health impacts daily functioning, psychological well-being, social participation, and perceived overall health [1, 2]. The EuroQol 5-Dimension (EQ-5D) instrument describes health across five dimensions and converts those into one single preference-based utility index scaled from 0 (death) to 1 (full health) [3]. EQ-5D utilities are commonly used in population health research and economic evaluation, where they are combined with survival to estimate quality-adjusted life years (QALYs) [4, 5].

Cardiometabolic conditions, such as cardiovascular disease, type 2 diabetes, and obesity, share common risk factors and contribute substantially to morbidity and mortality in the U.S., with this burden expected to persist [6–8]. These conditions are associated with limitations in physical functioning, daily activities, and lower HRQoL [9–11]. Over the past two decades, advances in treatment and management, including antihypertensive agents, statins, and glucose-lowering medications, may have improved symptoms and physical functioning among adults living with cardiometabolic conditions [12–15]. However, these gains may have been offset by the growing number of adults living with long-term complications, multimorbidity, and more severe obesity [16]. Together, these opposing forces make it unclear whether the population-level HRQoL burden of cardiometabolic conditions has improved, worsened, or remained stable over time [17].

Despite extensive research on HRQoL in cardiometabolic conditions, gaps remain in understanding how condition-attributed HRQoL decrements have changed over time at population-level. First, much of the evidence comes from clinical cohorts using condition-specific patient-reported outcome measures, such as the Kansas City Cardiomyopathy Questionnaire for heart failure [18–22]. These measures are useful for assessing outcomes within a specific disease but are not designed to compare HRQoL across conditions or estimate burden in the general population. Second, although nationally representative utility data, such as those from the Medical Expenditure Panel Survey (MEPS), can support comparisons across cardiometabolic conditions, many published estimates are based on earlier periods and may not reflect more recent patterns oftreatment and management [23, 24]. Third, because cardiometabolic conditions share common risk factors, inconsistent adjustment for obesity or body mass index (BMI) may bias condition-specific HRQoL estimates and limit comparability across conditions [25].

To assess whether condition-associated HRQoL decrementshave changed during a period of evolving cardiometabolic treatment in the United States, we used nationally representative MEPS data to: (1) estimate associations of sociodemographic characteristics, BMI, and cardiometabolic conditions (diabetes, heart disease, high blood pressure, high cholesterol, stroke, and obesity) with HRQoL, measured by EQ-5D utility; (2) evaluate temporal trends in condition-associated HRQoL decrements from 2001 to 2022; and (3) provide updated, nationally representative utility estimates for population health research and economic evaluation.

## Methods

### Data

We used data from the MEPS, a nationally representative survey of the U.S. civilian noninstitutionalized population. MEPS has collected the 12-Item Short Form Survey (SF-12) since 2001, directly collected EQ-5D measurement from 2000 to 2003, and included detailed sociodemographic characteristics and chronic condition indicators [26]. We analyzed adults aged ≥ 18 years in the 2001–2022 MEPS Full-Year Consolidated Public Use Files, limiting analyses to Self-Administered Questionnaire (SAQ) respondents because BMI and HRQoL measures were obtained from the SAQ. All anslyses incorporated SAQ person-level survey weights, primary sampling units, and stratification variables. Year-specific analyses used the original SAQ weights, whereas pooled analyses divided SAQ weights by the number of pooled survey years so that estimates represent the average annual U.S. adult population. Survey years 2017, 2019, and 2021 were excluded from the analysis because BMI data were not collected. Survey year 2020 was excluded from the main analysis to avoid conflating secular trends with pandemic-related changes, but its influence was evaluated in sensitivity analyses.

### Cardiometabolic conditions

MEPS priority condition indicators were used to identify diagnosed diabetes, heart disease, high blood pressure, high cholesterol (available from 2005 onward), and stroke. These indicators were selected because they are measured consistently across survey years and therefore support temporal comparisons of condition-associated HRQoL. Obesity was defined using self-reported BMI with standard cut points: underweight (< 18.5 kg/m^2^), normal weight (18.5–24.9), overweight (25.0-29.9), obesity class I (30.0–34.9), obesity class II (35.0–39.9), and obesity class III (≥ 40) [27].

### Mapping EQ-5D from SF-12

To obtain EQ-5D utilities consistently across study years, we applied the fully specified censored least absolute deviations (CLAD) mapping algorithm, which predicts EQ-5D utilities from the SF-12 scores [28]. Among the specifications evaluated in the original validation study, including ordinary least squares, Tobit, and simplified CLAD variants, the fully specified CLAD model had the lowest mean absolute error in an independent MEPS validation sample (MAE = 0.073; 95% CI, 0.071–0.075). The mapping model included the Physical Component Summary (PCS-12), Mental Component Summary (MCS-12), sociodemographic characteristics (age, sex, race/ethnicity, income, education), and the number of chronic conditions (NCC) derived from MEPS priority condition indicators. Predicted EQ-5D values were bounded to the 0–1 scale.

### Statistical analysis

We used survey-weighted multivariable linear regression models to estimate associations of sociodemographic characteristics, BMI, and cardiometabolic conditions with HRQoL, measured by the EQ-5D utility index. Linear models were used because their coefficients can be interpreted as adjusted mean differences in HRQoL utilities, allowing direct estimation of condition-specific HRQoL decrements and comparisons across years. Because the association between BMI and HRQoL may be nonlinear, with utilities generally lower in underweight range, highest in the normal-weight range, and lower at overweight and obesity range, ,BMI was modeled using linear and quadratic terms centered at 25 kg/m^2^, the WHO-defined threshold between normal weight and overweight. The linear term represents the estimated change in EQ-5D utility per 1 kg/m^2^ difference in BMI at BMI 25 kg/m^2^, whereas the quadratic term captures curvature in the BMI–HRQoL association [29].

We estimated four nested specifications: (1) sociodemographic covariates only (age group, sex, race/ethnicity, education, income); (2) sociodemographic covariates and cardiometabolic conditions (diabetes, heart disease, high blood pressure, high cholesterol, obesity, stroke); (3) sociodemographic covariates, BMI (centered at 25 kg/m^2^), and BMI^2^; and (4) sociodemographic covariates, cardiometabolic conditions, BMI, and BMI^2^. Comparing coefficients and model fit across these nested specifications allowed us to examine overlapping and independent associations of BMI and cardiometabolic conditions with HRQoL, and to assess whether gradients in the sociodemographic-attributable HRQoL persisted after adjustment. In model 4, obesity indicator (BMI ≥ 30 kg/m^2^) and continuous BMI terms were retained because they served different aspects of BMI-related HRQoL:the binary obesity classification and the continuous BMI-HRQoL dose-response relationship. We estimated the adjusted generalized variance inflation factors (GVIF) on 2022 data to assess potential collinearity between the obesity indicator and the continuous BMI terms. Model fit was assessed using the Akaike Information Criterion (AIC), comparing models 2, 3, and 4 with model 1 for each survey year, and comparing model 4 with model 2 to evaluate the added contribution of BMI terms. 

We fit year-specific models to estimate condition-specific HRQoL decrements, defined as the adjusted difference in HRQoL between adults with and without each cardiometabolic condition, corresponding to the regression coefficient for the condition indicator. For each condition, we assessed temporal trends using DerSimonian–Laird random-effects meta-regression, regressing year-specific decrements on calendar year centered at 2012 and weighting each estimate by the inverse of its variance [30]. * P*-values for trend slopes were adjusted for multiple comparisons across the six conditions using the Benjamini–Hochberg false discovery rate procedure. Linear trend models were estimated for all conditions; quadratic terms were added when inspection of year-specific estimates indicated nonlinearity. Coefficients moving toward 0 were interpreted as attenuation of HRQoL decrements, whereas coefficients moving away from 0 were interpreted as increases in magnitude.

Finally, to describe contemporary patterns with greater precision, we pooled data from recent survey years with available BMI data: 2015, 2016, 2018, and 2022. In this pooled recent-period analysis, we estimated associations of sociodemographic characteristics, BMI, and cardiometabolic conditions with HRQoL.

### Sensitivity analyses

Six sensitivity analyses were conducted to evaluate the robustness of the primary findings to survey-year exclusions, missing BMI data, ceiling effects, serial correlation, multimorbidity, and self-reported BMI bias. First, to assess whether excluding survey year 2020 affected the recent-period estimates, we re-estimated the pooled recent-period model after adding 2020 to the primary pooled years of 2015, 2016, 2018, and 2022. Second, to assess whether excluding cycles without BMI biased the temporal trend estimates, we examined high blood pressure-associated HRQoL decrements across all survey years because high blood pressure was the cardiometabolic condition most strongly correlated with BMI.We added a BMI-missing year indicator and an interaction between calendar year and BMI-missing year to test whether trends differed in years without BMI data. Third, we examined whether ceiling effects (EQ-5D utilities are bounded between 0 and 1 and have ceiling observations at 1.0) affected the estimated condition-specific decrements through repeating the primary pooled regression after excluding observations with EQ-5D = 1.0, and comparing the results with the main analysis. Fourth, to address potential serial correlation in the meta-regression, all condition-specific trend analyses were re-estimated using a continuous-time AR(1) random-effects structure. This model allowed correlation between year-specific estimates to decrease as calendar-time distance increased and accommodated unequally spaced survey years. Fifth, to examine whether co-occurring cardiometabolic conditions were associated with HRQoL beyond their additive main effects, we added two-way interaction terms for five most prevalent co-occurring condition pairs in the pooled 2015, 2016, 2018, and 2022 data. Those are high blood pressure and high cholesterol, high blood pressure and obesity, heart disease and high blood pressure, diabetes and high blood pressure, and diabetes and obesity. Sixth, because BMI and cardiometabolic conditions were based on self-report, we assessed sensitivity to self-reported BMI bias by re-estimating the primary pooled regression after applying the Stommel and Schoenborn correction : corrected BMI = (self-reported BMI − 2.204) / 0.922 [31]. The binary obesity indicator and continuous BMI terms were then recomputed using the corrected BMI values. All other model specifications remained unchanged.

## Results

### Population characteristics and HRQoL, 2001–2022

**Fig. 1:**
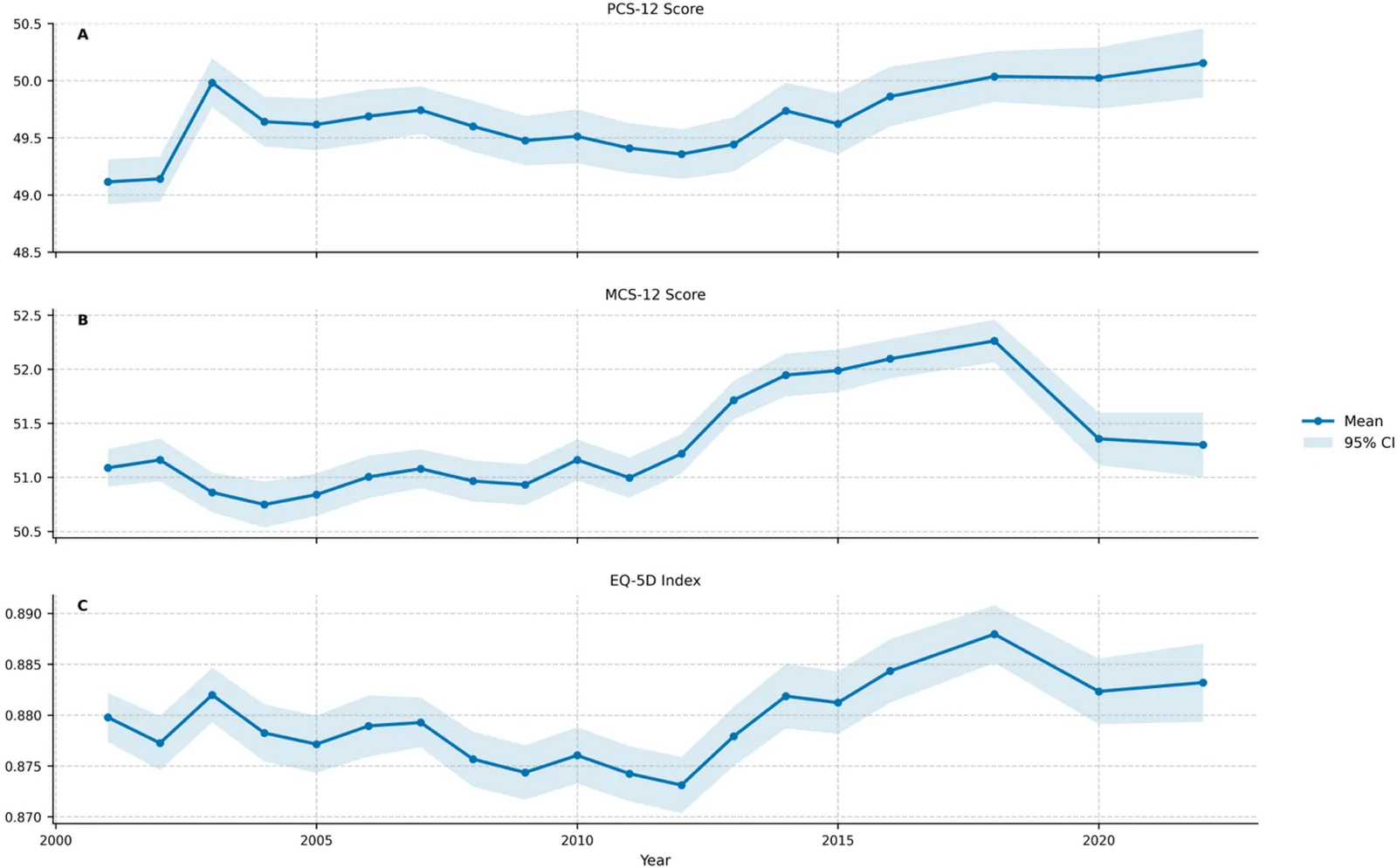
Trends in PCS-12, MCS-12, and EQ-5D index among U.S. adults, MEPS 2001–2022

Overall HRQoL, as measured by EQ-5D, PCS-12, and MCS-12, increased over the study period from 2001 to 2022, with all three measures consistently lower during the earlier decade (2001–2012) than during the later decade (2013–2022) , presented in Fig. 1. The EQ-5D index declined from the early 2000s to its lowest point in 2012 at 0.873 (95% CI, 0.870–0.876), then increased steadily to its highest point in 2018 at 0.888 (95% CI, 0.885–0.891), then declining in 2020 to 0.882 (95% CI, 0.879–0.886) and remaining relatively stable thereafter (2022: 0.883; 95% CI, 0.879–0.887). MCS-12 followed a similar pattern: it reached its lowest point in 2004 at 50.748 (95% CI, 50.536–50.959), then increased substantially through 2018, when it reached its highest point at 52.261 (95% CI, 52.064–52.458), before declining sharply in 2020 to 51.356 (95% CI, 51.113–51.599) and remaining lower in 2022 (51.301; 95% CI, 51.002–51.601). In contrast, PCS-12 showed a more gradual upward trend over the study period, with its lowest value in 2001 at 49.114 (95% CI, 48.917–49.311) and its highest value in 2022 at 50.155 (95% CI, 49.853–50.457).

HRQoL differed by sociodemographic characteristics, cardiometabolic conditions, and BMI category (Table 1). Survey-weighted pooled estimates from 2001 to 2022 represented an average annual population of 229.3 million U.S. adults. Mean HRQoL was higher among younger than older adults (0.915 for ages 30–39 vs. 0.840 for ages 60–69) and in men than women (0.891 vs. 0.869). By race and ethnicity, mean HRQoL was highest among non-Hispanic Asian adults (0.910), followed by Hispanic (0.895), non-Hispanic White (0.876), and non-Hispanic Black adults (0.870). HRQoL also increased with socioeconomic status, from 0.819 among adults with family income ≤ 100% federal poverty level to 0.917 among those with income ≥ 400%, and from 0.841 among adults with no degree to 0.917 among those with master's or doctoral degrees.

**Table 1:** Weighted sociodemographic characteristics, comorbidity burden, and HRQoL measures among U.S. adults, MEPS 2001–2022 (*N* = 229,317,502)

		% of population	Age (Years)		NCC		PCS-12		MCS-12		EQ-5D	
			Mean	SE	Mean	SE	Mean	SE	Mean	SE	Mean	SE
MEPS weighted population		100.00	46.87	0.11	1.86	0.01	49.651	0.048	51.320	0.035	0.879	0.001
*Age group*												
18–29		20.88	23.71	0.03	0.64	0.01	54.374	0.041	51.333	0.063	0.932	0.001
30–39		17.74	34.49	0.02	1.03	0.01	53.109	0.052	50.832	0.060	0.915	0.001
40–49		18.02	44.50	0.02	1.55	0.01	51.008	0.064	50.780	0.057	0.891	0.001
50–59		17.56	54.36	0.02	2.30	0.02	48.372	0.082	51.059	0.062	0.862	0.001
60–69		13.12	64.15	0.02	3.07	0.02	45.778	0.106	52.218	0.072	0.840	0.001
70–79		8.07	74.07	0.02	3.61	0.02	42.624	0.117	52.703	0.078	0.810	0.001
80+		4.62	83.26	0.02	3.67	0.02	37.875	0.138	51.264	0.117	0.756	0.002
*Sex*												
Female		51.74	47.59	0.11	1.93	0.01	48.953	0.057	50.476	0.041	0.869	0.001
Male		48.26	46.09	0.11	1.79	0.01	50.399	0.049	52.224	0.038	0.891	0.001
*Race*												
Non-Hispanic white		66.74	48.92	0.13	2.01	0.01	49.416	0.058	51.229	0.042	0.876	0.001
Non-Hispanic black		11.49	44.29	0.16	1.95	0.02	48.867	0.093	51.419	0.074	0.870	0.001
Hispanic		14.42	40.88	0.15	1.36	0.01	50.929	0.080	51.485	0.072	0.895	0.001
Non-Hispanic Asian/Pacific Islander		5.23	44.33	0.39	1.09	0.03	51.490	0.127	52.360	0.111	0.910	0.002
Non-Hispanic other		2.12	43.29	0.32	2.17	0.05	48.059	0.248	49.944	0.204	0.855	0.003
*Education*												
No degree		14.05	46.02	0.18	1.94	0.02	46.917	0.102	49.937	0.075	0.841	0.001
High school diploma		48.23	46.44	0.12	1.95	0.01	48.999	0.053	51.224	0.043	0.871	0.001
Other degree		6.83	47.02	0.20	1.93	0.02	50.061	0.102	51.371	0.092	0.885	0.001
Bachelor’s degree		17.87	46.31	0.17	1.58	0.01	52.156	0.063	51.983	0.059	0.912	0.001
Masters or Doctorate degree		9.77	50.96	0.19	1.75	0.02	52.203	0.075	52.595	0.071	0.917	0.001
I*ncome (% poverty level)*												
Poor (< 100%)		11.18	43.56	0.17	2.04	0.02	46.182	0.094	47.861	0.088	0.819	0.001
Near poor (100–124%)		4.01	48.32	0.29	2.22	0.03	46.005	0.144	49.162	0.115	0.829	0.002
Low income (125–199%)		12.90	47.64	0.19	2.04	0.02	47.477	0.084	50.210	0.072	0.852	0.001
Middle income (200–399%)		30.22	45.83	0.13	1.82	0.01	49.807	0.057	51.441	0.049	0.882	0.001
High income ( > = 400%)		41.69	48.14	0.12	1.76	0.01	51.491	0.047	52.710	0.040	0.907	0.001
*Condition*												
Type 2 diabetes		9.14	60.99	0.14	4.65	0.02	40.713	0.111	49.491	0.093	0.769	0.001
Heart disease		13.05	61.81	0.19	4.35	0.02	41.402	0.111	49.668	0.085	0.778	0.001
High blood pressure		30.70	59.48	0.12	3.74	0.01	43.999	0.072	50.510	0.056	0.810	0.001
High cholesterol		24.57	59.26	0.13	3.83	0.02	45.121	0.093	50.736	0.062	0.822	0.001
Stroke		3.36	66.88	0.20	5.10	0.03	36.790	0.157	47.787	0.160	0.718	0.002
Obesity		28.66	48.00	0.11	3.12	0.01	46.801	0.069	50.519	0.051	0.842	0.001
*BMI (kg/m* ^2^ *)*												
underweight (< 18.5)		1.85	43.75	0.35	1.22	0.03	48.973	0.187	49.989	0.163	0.868	0.002
normal weight (18.5–24.9)		33.33	44.36	0.07	1.15	0.01	51.708	0.035	51.548	0.034	0.904	0.000
overweight (25.0-29.9)		33.47	48.43	0.06	1.57	0.01	50.272	0.035	51.904	0.033	0.890	0.000
obesity class I (30.0-34.9)		17.13	48.65	0.08	2.96	0.01	48.099	0.053	51.097	0.048	0.858	0.001
obesity class II (35.0-39.9)		7.05	47.72	0.12	3.26	0.02	46.065	0.089	50.298	0.079	0.833	0.001
obesity class III (≥ 40)		4.48	45.97	0.14	3.54	0.02	42.991	0.120	48.699	0.107	0.795	0.001

NCC, Number of Chronic Conditions (from Sullivan & Ghushchyan CLAD mapping algorithm); PCS-12, Physical Component Summary Score; MCS-12, Mental Component Summary Score (both from SF-12, scored to mean 50 ± SD 10 in U.S. general population). EQ-5D, utility index mapped from PCS-12 and MCS-12 using Sullivan & Ghushchyan (2006) algorithm. All statistics are weighted using MEPS SAQ person-level weights across survey years 2001–2022 (excluding 2017, 2019, 2021 and 2020). SE estimated using effective sample size approximation. Condition rows show prevalence (% of all adults) and mean HRQoL among adults with that condition

Across cardiometabolic conditions, mean HRQoL was lowest among adults with stroke (0.718), followed by diabetes (0.769), heart disease (0.778), high blood pressure (0.810), high cholesterol (0.822), and obesity (0.842). HRQoL exhibited an inverse U-shaped pattern across BMI categories with the highest mean utility among adults in the normal-weight category (0.904) and lower values among adults who were underweight (0.868), overweight (0.890), or had obesity, with progressively lower values at higher obesity classes (≤ 0.858).

**Fig. 2:**
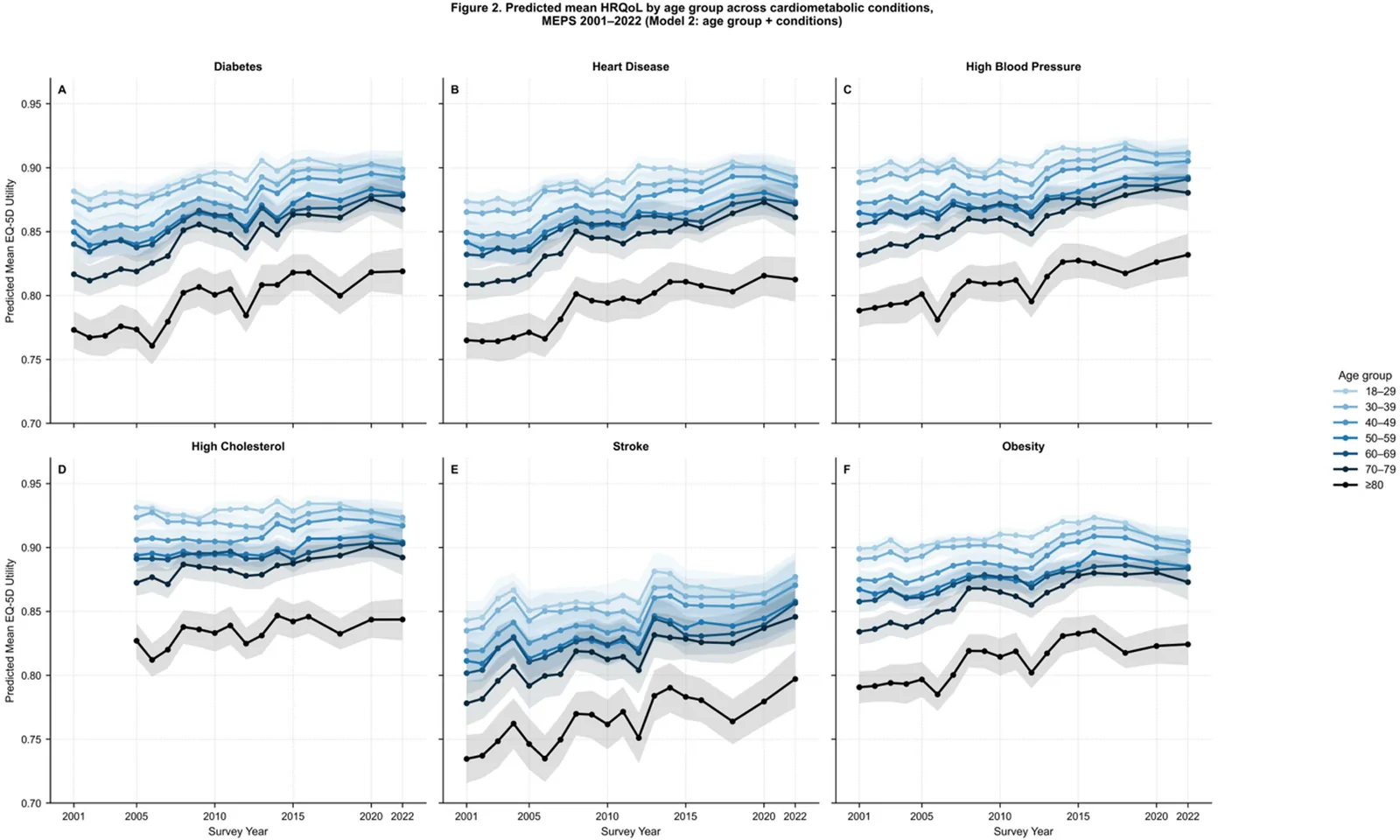
Predicted mean HRQoL by age group across cardiometabolic conditions, 2001–2022 (Model 2)

Mean HRQoL increased over time across age groups, with larger gains among older adults, particularly aged 70–79 and ≥ 80 years (Fig. 2). HRQoL improvements were also observed over time across sex, race and ethnicity, education, and income subgroups (Supplementary Fig. S1-S4). Because subgroup trajectories were broadly similar and became more parallel after 2015, we pooled 2015, 2016, 2018, and 2022 to estimate associations for recent periods.

### Association between sociodemographic, BMI, cardiometabolic conditions and HRQoL

Compared with the sociodemographic-only specification (model 1), adding cardiometabolic conditions (model 2), BMI with a quadratic term (model 3), or both (model 4) improved model fit in the aggregated data from 2015, 2016, 2018, and 2022 (Table 2) and in each survey year (Supplementary Table S3-S21). The fully adjusted model had the lowest AIC in all 19 survey years. Compared with model 1, the ΔAIC for model 4 ranged from − 896 to − 3,432. Adding cardiometabolic conditions produced the largest improvement in fit (model 2 vs. model 1; ΔAIC range, − 712 to − 3,227), whereas adding BMI terms to cardiometabolic conditions provided additional but smaller improvement (model 4 vs. model 2; ΔAIC range, − 20 to − 314; Supplemental Table S2). Although the incremental improvement from BMI was smaller than that from cardiometabolic conditions, BMI terms were statistically significant in models 3 and 4, supporting an independent association between BMI and HRQoL after adjustment for cardiometabolic conditions.

Collinearity between the obesity indicator and continuous BMI terms was not severe. The adjusted GVIFs for the obesity indicator, linear BMI, and quadratic BMI were 1.68, 2.29, and 1.78, respectively, all below 3.16 (Supplementary Table S1). These results suggest that including both the obesity indicator and continuous BMI terms was unlikely to meaningfully inflate standard errors or affect inference.

Adjustment for cardiometabolic conditions substantially attenuated the gradient in the age-attributable HRQoL, whereas adjustment for BMI alone resulted in only modest attenuation. In contrast, marginal effects of sex, race/ethnicity, education, and income changed little after adjustment for cardiometabolic conditions and/or BMI, with subgroup rankings and direction of associationspreserved across models. BMI and cardiometabolic conditions showed partially overlapping associations with HRQoL. Relative to models adjusted for cardiometabolic conditions only, additional adjustment for BMI modestly attenuated cardiometabolic condition-specific HRQoL decrements in the pooled 2015, 2016, 2018, and 2022 MEPS data, most notably for obesity (− 0.0311 to − 0.0143), diabetes (− 0.0427 to − 0.0411) (Table 2). Conversely, relative to models adjusted for BMI only, additional adjustment for cardiometabolic conditions attenuated the BMI-specific HRQoL decrements. The linear BMI coefficient changed from − 0.0033 to − 0.0014 per 1 kg/m^2^ after adjustment for cardiometabolic conditions. Despite this attenuation, both condition-specific and BMI-specific HRQoL decrements remained statistically significant in the fully adjusted model.

**Table 2:** Survey-weighted multivariable linear regression of HRQoL on sociodemographic characteristics, BMI, and cardiometabolic conditions, MEPS 2015, 2016, 2018, 2022

Variable	Model 1: sociodemographic			Model 2: sociodemographic + CMD			Model 3: sociodemographic + BMI			Model 4: sociodemographic + CMD + BMI (Primary)		
	β	(SE)	*p*	β	(SE)	*p*	β	(SE)	*p*	β	(SE)	*p*
Intercept	0.8493	(0.0027)	< 0.001	0.8687	(0.0025)	< 0.001	0.8528	(0.0029)	< 0.001	0.8671	(0.0026)	< 0.001
*Age group (ref: 18–29 years)*												
30–39 years	− 0.0264	(0.0018)	< 0.001	− 0.0149	(0.0016)	< 0.001	− 0.0197	(0.0017)	< 0.001	− 0.0140	(0.0016)	< 0.001
40–49 years	− 0.0484	(0.0019)	< 0.001	− 0.0240	(0.0018)	< 0.001	− 0.0387	(0.0019)	< 0.001	− 0.0230	(0.0018)	< 0.001
50–59 years	− 0.0764	(0.0019)	< 0.001	− 0.0376	(0.0018)	< 0.001	− 0.0674	(0.0020)	< 0.001	− 0.0370	(0.0018)	< 0.001
60–69 years	− 0.0994	(0.0023)	< 0.001	− 0.0427	(0.0021)	< 0.001	− 0.0910	(0.0023)	< 0.001	− 0.0428	(0.0021)	< 0.001
70–79 years	− 0.1197	(0.0027)	< 0.001	− 0.0473	(0.0025)	< 0.001	− 0.1134	(0.0026)	< 0.001	− 0.0483	(0.0025)	< 0.001
≥80 years	− 0.1653	(0.0032)	< 0.001	− 0.0875	(0.0032)	< 0.001	− 0.1641	(0.0031)	< 0.001	− 0.0894	(0.0032)	< 0.001
*Sex (ref: Female)*												
Male	0.0131	(0.0009)	< 0.001	0.0172	(0.0009)	< 0.001	0.0131	(0.0009)	< 0.001	0.0172	(0.0009)	< 0.001
*Race/Ethnicity (ref: Non-Hispanic White)*												
Non-hispanic black	0.0081	(0.0019)	< 0.001	0.0143	(0.0017)	< 0.001	0.0136	(0.0018)	< 0.001	0.0150	(0.0017)	< 0.001
Hispanic	0.0292	(0.0018)	< 0.001	0.0243	(0.0015)	< 0.001	0.0287	(0.0017)	< 0.001	0.0245	(0.0015)	< 0.001
Non-Hispanic Asian/Pacific Islander	0.0189	(0.0026)	< 0.001	0.0094	(0.0022)	< 0.001	0.0084	(0.0024)	< 0.001	0.0078	(0.0022)	< 0.001
Non-Hispanic Other	− 0.0140	(0.0036)	< 0.001	− 0.0083	(0.0034)	0.016	− 0.0119	(0.0036)	0.001	− 0.0082	(0.0034)	0.016
*Education (ref: No degree)*												
High school diploma / GED	0.0132	(0.0019)	< 0.001	0.0139	(0.0017)	< 0.001	0.0167	(0.0019)	< 0.001	0.0147	(0.0017)	< 0.001
Other post-secondary degree	0.0201	(0.0028)	< 0.001	0.0200	(0.0026)	< 0.001	0.0233	(0.0029)	< 0.001	0.0207	(0.0027)	< 0.001
Bachelor’s degree	0.0410	(0.0021)	< 0.001	0.0335	(0.0019)	< 0.001	0.0409	(0.0021)	< 0.001	0.0341	(0.0019)	< 0.001
Master’s or Doctorate degree	0.0531	(0.0024)	< 0.001	0.0439	(0.0021)	< 0.001	0.0516	(0.0024)	< 0.001	0.0442	(0.0021)	< 0.001
*Income (ref: Poor*,* < 100% FPL)*												
Near poor (100–124%)	0.0224	(0.0037)	< 0.001	0.0205	(0.0034)	< 0.001	0.0224	(0.0037)	< 0.001	0.0207	(0.0034)	< 0.001
Low income (125–199%)	0.0366	(0.0027)	< 0.001	0.0340	(0.0025)	< 0.001	0.0374	(0.0026)	< 0.001	0.0342	(0.0025)	< 0.001
Middle income (200–399%)	0.0602	(0.0023)	< 0.001	0.0540	(0.0022)	< 0.001	0.0611	(0.0023)	< 0.001	0.0540	(0.0022)	< 0.001
High income (≥ 400%)	0.0819	(0.0023)	< 0.001	0.0708	(0.0022)	< 0.001	0.0803	(0.0023)	< 0.001	0.0703	(0.0022)	< 0.001
*Cardiometabolic conditions*												
Type 2 diabetes				− 0.0427	(0.0023)	< 0.001				− 0.0411	(0.0023)	< 0.001
Heart disease				− 0.0501	(0.0019)	< 0.001				− 0.0500	(0.0019)	< 0.001
High blood pressure				− 0.0329	(0.0015)	< 0.001				− 0.0313	(0.0015)	< 0.001
High cholesterol				− 0.0236	(0.0014)	< 0.001				− 0.0232	(0.0014)	< 0.001
Stroke				− 0.0707	(0.0036)	< 0.001				− 0.0710	(0.0036)	< 0.001
Obesity (BMI ≥ 30)				− 0.0311	(0.0012)	< 0.001				− 0.0143	(0.0019)	< 0.001
*Body mass index*												
BMI − 25 kg/m^2^ (linear)							− 0.0033	(0.0002)	< 0.001	− 0.0014	(0.0002)	< 0.001
(BMI − 25)^2^ (quadratic)							− 0.000010	(0.000017)	0.554	− 0.000017	(0.000014)	0.228

Survey-weighted linear regression (svyglm, Gaussian family) pooling MEPS 2015, 2016, 2018, and 2022. Sampling weights divided by 4 to represent the average annual U.S. adult population. BMI − 25 (linear) estimates the mean change in EQ-5D per 1 kg/m^2^ increase in BMI at 25 kg/m^2^; the quadratic term captures curvature of the BMI–HRQoL association. FPL, Federal poverty level; β, regression coefficient (adjusted EQ-5D utility difference); SE, standard error

### Temporal trends in cardiometabolic condition-specific HRQoL decrements

**Fig. 3:**
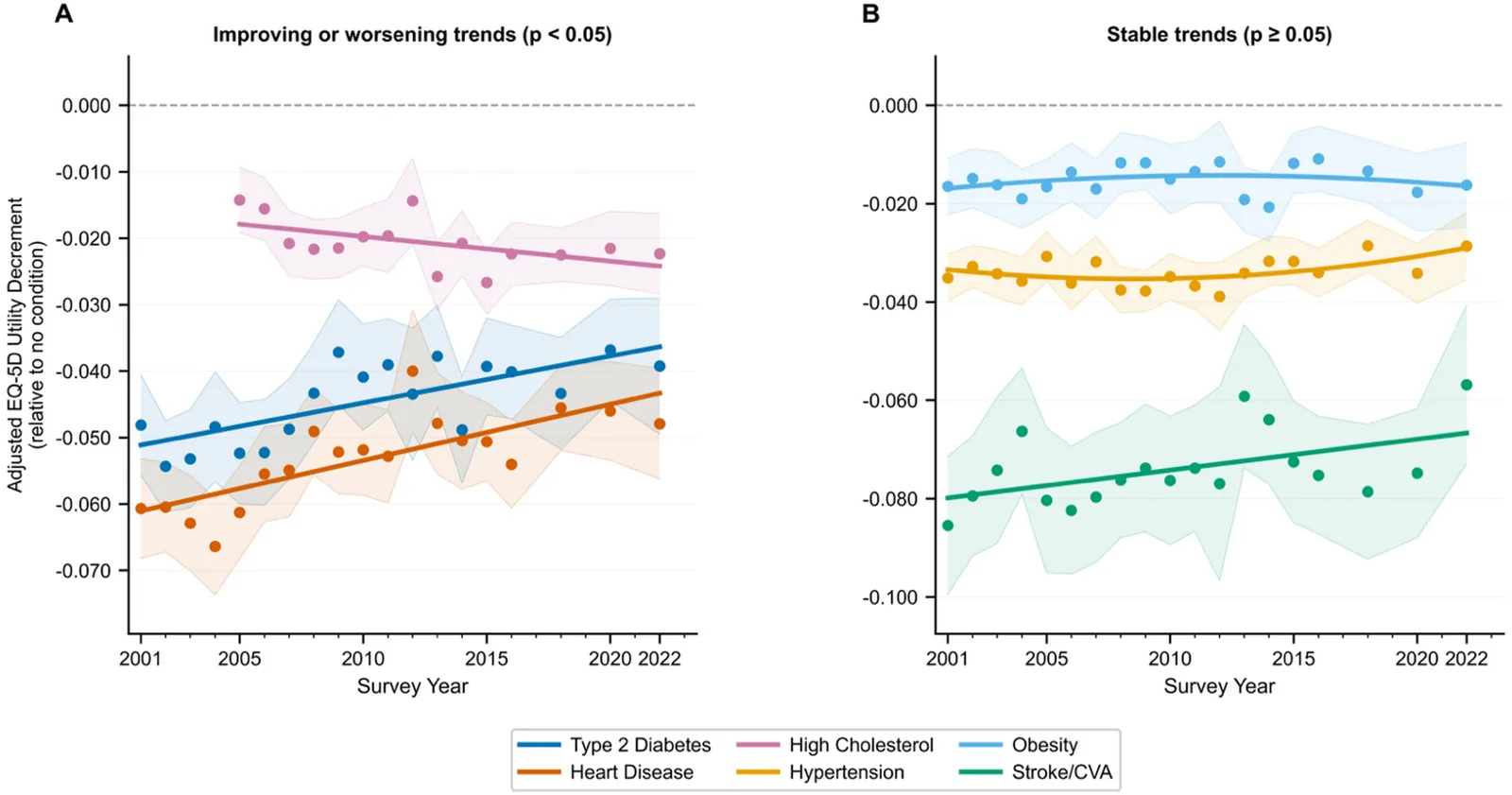
Cardiometabolic condition-associated HRQoL decrements over time, MEPS 2001–2022

Meta-regression indicated that diabetes- and heart disease-specific HRQoL decrements decreased in magnitude from 2001 to 2022, whereas the high cholesterol-associated decrement increased in magnitude from 2005 to 2022 (Fig. 3; Supplementary Table S22–S23). The diabetes-associated decrement changed from − 0.0500 in 2001 to − 0.0414 in 2022 (annual change + 0.0007, *P* < 0.0001), and the heart disease-associated decrement attenuated from − 0.0611 to − 0.0487over the same period (annual change + 0.0007, *P* < 0.0001). Stroke-associated decrements also trended toward attenuation (− 0.0854 in 2001 to − 0.0562 in 2022), although the linear trend did not reach statistical significance (annual change + 0.0005, *P* = 0.0844).

In contrast, the high cholesterol-associated decrement became more negative, from − 0.0147 in 2005 to − 0.0223 in 2022 (annual change − 0.0004, *P* = 0.0185). High blood pressure- and obesity-associated decrements exhibited nonlinear (quadratic) time patterns. High blood pressure attributed to the largest decrement around 2012, changing from − 0.0361 in 2001 to − 0.0404 in 2012 and − 0.0313 in 2022). Obesity-associated decrement was smallest around 2012, changing from − 0.0311 in 2001 to − 0.0290 in 2012, before increasing in magnitude to − 0.0370 in 2022.

### Sensitivity analyses

First, including 2020 in the recent-period pooled analysis did not significantly change condition-specific estimates. Coefficients for all six cardiometabolic conditions were within 0.003 EQ-5D utility units of the primary estimates, and statistical significance did not change for any condition (Supplementary Table S24).

Second, in the association between high blood pressure and EQ-5D across all 22 survey cycles, neither the BMI-missing year indicator nor the interaction between calendar year and BMI-missing year iwas statistically significant (*P* = 0.131 and *P* = 0.077, respectively). These findings suggest that BMI-missing years did not have systematically different high blood pressure–associated HRQoL patterns compared with the primary analytic years making it unlikely that excluding BMI-missing cycles meaningfully biased the primary temporal trend estimates (Supplementary Tables S25–S26).

Third, excluding observations at the EQ-5D ceiling, defined as EQ-5D=1.0, produced results similar to the primary analysis. These ceiling observations represented 9.3% of the pooled 2015, 2016, 2018, and 2022 MEPS sample (6,548 of 70,601 observations). After exclusion, condition-specific coefficients were within 0.003 EQ-5D utility units of the primary estimates for all six conditions (Supplementary Table S27). Signs and statistical significance were unchanged across all conditions, suggesting that ceiling observations did not meaningfully affect the estimated condition-specific HRQoL decrements.

Fourth, after accounting for potential correlation between adjacent survey years using a continuous-time AR(1) structure, condition-specific trend estimates were highly consistent with the primary DerSimonian–Laird analysis; slope differences were less than 0.00005 EQ-5D units per year for five of six conditions. The largest difference was observed for obesity (0.000146 EQ-5D units per year) but the trend remained non-significant in both analyses. Directional conclusions and significance patterns were preserved across all conditions (Supplementary Table S28).

Fifth, in the pooled multimorbidity interaction model using 2015, 2016, 2018, and 2022 data, two of five two-way interaction terms were statistically significant (Table 3). Heart disease × high blood pressure showed additional HRQoL loss beyond individual associations (β = − 0.0112; 95% CI, − 0.0177 to − 0.0047;* P* = < 0.001). Type 2 diabetes × hypertension showed a smaller combined decrement than under an additive model (β = 0.0092; 95% CI, 0.0002 to 0.0182; *P* = 0.044). Main-effect estimates for all six conditions were stable between the additive and interaction models.

**Table 3:** Multimorbidity interaction sensitivity model, MEPS 2015, 2016, 2018, and 2022

Variable	Additive model			Interaction model		
	β	(SE)	*p*	β	(SE)	*p*
Intercept	0.8671	(0.0026)	< 0.001	0.8666	(0.0026)	< 0.001
*Age group (ref: 18–29 years)*						
30–39 years	− 0.0140	(0.0015)	< 0.001	− 0.0140	(0.0015)	< 0.001
40–49 years	− 0.0230	(0.0016)	< 0.001	− 0.0228	(0.0016)	< 0.001
50–59 years	− 0.0370	(0.0017)	< 0.001	− 0.0368	(0.0017)	< 0.001
60–69 years	− 0.0428	(0.0019)	< 0.001	− 0.0428	(0.0019)	< 0.001
70–79 years	− 0.0483	(0.0022)	< 0.001	− 0.0483	(0.0022)	< 0.001
≥80 years	− 0.0894	(0.0031)	< 0.001	− 0.0895	(0.0031)	< 0.001
*Sex (ref: Female)*						
Male	0.0172	(0.0009)	< 0.001	0.0172	(0.0009)	< 0.001
*Race/Ethnicity (ref: non-hispanic white)*						
Non-Hispanic Black	0.0150	(0.0016)	< 0.001	0.0149	(0.0016)	< 0.001
Hispanic	0.0245	(0.0015)	< 0.001	0.0244	(0.0015)	< 0.001
Non-Hispanic Asian/Pacific Islander	0.0078	(0.0021)	< 0.001	0.0079	(0.0021)	< 0.001
Non-Hispanic Other	− 0.0082	(0.0031)	0.008	− 0.0081	(0.0030)	0.008
*Education (ref: No degree)*						
High school diploma / GED	0.0147	(0.0016)	< 0.001	0.0146	(0.0016)	< 0.001
Other post-secondary degree	0.0207	(0.0026)	< 0.001	0.0206	(0.0026)	< 0.001
Bachelor’s degree	0.0341	(0.0019)	< 0.001	0.0341	(0.0019)	< 0.001
Master’s or Doctorate degree	0.0442	(0.0021)	< 0.001	0.0443	(0.0021)	< 0.001
*Income (ref: Poor*,* < 100% FPL)*						
Near poor (100–124%)	0.0207	(0.0034)	< 0.001	0.0207	(0.0034)	< 0.001
Low income (125–199%)	0.0342	(0.0025)	< 0.001	0.0343	(0.0025)	< 0.001
Middle income (200–399%)	0.0540	(0.0021)	< 0.001	0.0540	(0.0021)	< 0.001
High income (≥ 400%)	0.0703	(0.0021)	< 0.001	0.0704	(0.0021)	< 0.001
*Cardiometabolic conditions*						
Type 2 diabetes	− 0.0411	(0.0022)	< 0.001	− 0.0439	(0.0041)	< 0.001
Heart disease	− 0.0500	(0.0018)	< 0.001	− 0.0436	(0.0024)	< 0.001
High blood pressure	− 0.0313	(0.0014)	< 0.001	− 0.0312	(0.0021)	< 0.001
High cholesterol	− 0.0232	(0.0013)	< 0.001	− 0.0256	(0.0016)	< 0.001
Stroke/CVA	− 0.0710	(0.0035)	< 0.001	− 0.0709	(0.0035)	< 0.001
Obesity (BMI ≥ 30)	− 0.0143	(0.0018)	< 0.001	− 0.0120	(0.0019)	< 0.001
*Body mass index*						
BMI − 25 kg/m^2^ (linear)	− 0.0014	(0.0002)	< 0.001	− 0.0014	(0.0002)	< 0.001
(BMI − 25)^2^ (quadratic)	− 0.000017	(0.000014)	0.233	− 0.000017	(0.000014)	0.236
*Two-way interactions (interaction model only)*						
High blood pressure × High cholesterol	–	–	–	0.0051	(0.0027)	0.060
High blood pressure × Obesity	–	–	–	− 0.0035	(0.0023)	0.127
Heart disease × High blood pressure	–	–	–	− 0.0112	(0.0033)	< 0.001
Type 2 diabetes × high blood pressure	–	–	–	0.0092	(0.0046)	0.044
Type 2 diabetes × Obesity	–	–	–	− 0.0071	(0.0041)	0.083

Survey-weighted linear regression (svyglm, Gaussian family) pooling MEPS 2015, 2016, 2018, and 2022. Sampling weights divided by 4. Additive model: Model 4 specification (sociodemographic covariates + six cardiometabolic conditions + BMI + BMI^2^) with no interaction terms. Interaction model: same specification plus five two-way interaction terms for the most prevalent co-occurring condition pairs. Positive interaction coefficient, sub-additive harm (co-occurrence less detrimental than sum of individual effects); negative, super-additive harm; β, regression coefficient (adjusted EQ-5D utility difference); SE, Standard error; FPL, Federal poverty level

Sixth, applying the Stommel and Schoenborn (2009) BMI correction did not meaningdully alter condition-specific HRQoL decrements. Coefficients for all six cardiometabolic conditions changed by less than 0.1%, and the obesity coefficient changed by 1.2%, from − 0.0143 to − 0.0141. The linear BMI coefficient changed by 6.8%, from − 0.00138 to − 0.00129, while the quadratic BMI term remained non-significant in both specifications. These findings indicate that self-reported BMI bias did not substantively affect the primary conclusions (Supplementary Table S29).

## Discussion

Using nationally representative MEPS data from 2001 to 2022 and a validated SF-12-to-EQ-5D mapping algorithm, we estimated associations between HRQoL and sociodemographic characteristics, BMI, and cardiometabolic conditions, assessed temporal trends in condition-specific HRQoL decrements, and generated nationally representative utility estimates for recent survey years in the past decade. Over the study period, the negative HRQOL impact associated with diabetes, heart disease, and stroke became less pronouced, whereas the impact associated with high cholesterol became more pronounced. High blood pressure and obesity showed nonlinear patterns, with the largest HRQoL decrements linked to high blood pressure in 2012 and obesity-related decrements becoming more pronounced in later years. 

In fully adjusted models, accounting for cardiometabolic conditions substantially attenuated the gradient in the age-attributable HRQoL with the largest improvements in condition-associated HRQoL observed among older adults. It suggests that higher cardiometabolic burden at older ages contributes to lower HRQoL. A residual gradient in age-attributed impact on HRQoL persisted, which may reflect other age-related conditions (e.g., chronic kidney disease, cancer, cognitive impairment) that are independently associated with HRQoL.

Adjustment for BMI attenuated condition-specific decrements, particularly diabetes and high blood pressure, suggesting that part of the observed association between these conditions and HRQoL is shared with BMI. This pattern is consistent with evidence that BMI may affect HRQoL both directly (e.g., through increased depression or anxiety) and indirectly (e.g., through raising the relative risk of cardiometabolic disease) [29, 32].

These findings have implications for health economic models of cardiometabolic conditions that alter body weight (e.g., lifestyle modification). Such models should consider (i) representing BMI as an explicit pathway to capture direct weight-related effects on HRQoL; (ii) avoiding double counting by assessing whether condition-specific utility estimates already adjust for BMI; and (iii) allowing BMI-HRQoL associations to vary by disease status when supported by empirical evidence (e.g., different BMI-utility slopes among adults with vs. without diabetes) [33]. More broadly, our results extend prior MEPS-based analyses of HRQoL, which examined associations with sociodemographic characteristics and clinical diseases but did not adjust for BMI despite its central role in cardiometabolic risk and population HRQoL [23, 34].

Diabetes-associated HRQoL decrements attenuated steadily from 2001 to 2022 (− 0.0500 to − 0.0414; annual change + 0.0007, *P* < 0.0001), indicating that the adjusted HRQoL difference between adults with and without diabetes became less negative over time. This pattern is consistent with national evidence of improved diabetes management and cardiovascular risk-factor control during 2000s, including increased use of antihypertensive and statin therapy among eligible adults and higher attainment of guideline-based targets [35]. Declines in major diabetes-related complications (e.g., acute myocardial infarction, stroke, amputation, and hyperglycemic crisis) may also have contributed to better health status and functioning among adults living with diabetes [36]. Our finding is consistent with prior MEPS-based evidence showing improved physical health among adults with diabetes, supporting the plausibility of an upward shift in population HRQoL during this period [37].

Heart disease-associated HRQoL decrements also attenuated from 2001 to 2022 (− 0.0611 to − 0.0487; annual change + 0.0007, *P* < 0.0001), indicating that the adjusted HRQoL gap between adults with and without heart disease became smaller over time. This finding is consistent with population surveillance data showing declines in angina symptom prevalence in U.S. adults, which may reflect a lower symptomatic burden of coronary disease and better functional status among adults with heart disease [38]. High cholesterol-associated HRQoL decrements became more negative from 2005 to 2022, in contrast to the attenuation observed for diabetes and heart disease. This pattern should be interpreted cautiously because high cholesterol is often asymptomatic and may be associated with HRQoL through comorbid conditions, medication use, perceived cardiovascular risk, or its role as a marker of broader cardiometabolic risk. Therefore, the increasing high cholesterol-associated decrement may reflect changes in case mix and comorbidity burden rather than a direct effect of cholesterol levels on HRQoL.

High blood pressure-associated HRQoL decrements followed a nonlinear pattern, reaching their most negative values around 2012 (− 0.0361 in 2001; − 0.0404 in 2012; − 0.0313 in 2022). This trajectory is consistent with national surveillance showing that high blood pressure control improved into the early 2010s but later declined, with more recent evidence suggesting partial recovery in control in subsequent years [39]. Changes in treatment intensity, risk-based management, complication risk, and the distribution of diagnosed hypertension severity may have contributed to this pattern.The 2017 ACC/AHA guideline’s emphasis on lower treatment thresholds and risk-based management may be consistent with improvements in health status among treated adults, although our analysis does not assess causal effects [40].

Obesity-associated HRQoL decrements followed an U-shaped temporal pattern, with the smallest decrement around 2012 (− 0.0290) and more negative decrements thereafter (− 0.0370 in 2022). This pattern aligns with substantial increases in obesity prevalence and, notably, growth in severe obesity. From 1999 to 2000 to 2017–2018, the age-adjusted prevalence of obesity increased from 30.5 to 42.4%, and severe obesity nearly doubled from 4.7 to 9.2% [41, 42]. A larger share of class II/III obesity may contribute to larger utility losses, particularly through mobility limitations, pain or discomfort, and reduced physical functioning. Changes in obesity-related comorbidity, functional limitation, and social or psychological burden may also help explain the more negative obesity-associated decrements observed after 2012. 

 The multimorbidity sensitivity analysis suggested that some cardiometabolic conditions may have nonadditive associations with HRQoL. The co-occurrence of heart disease and high blood pressure was associated with HRQoL decrements greater than the sum of their individual effects, consistent with the combined cardiovascular burden of these conditions. By contrast, type 2 diabetes co-occurring with high blood pressure showed sub-additive harm, a pattern that may reflect more intensive co-management of this recognized cardiovascular risk cluster. The stability of the primary condition-specific estimates confirms that the additive main-effects specification does not significantly bias the trend findings.

Overall, our condition-specific HRQoL estimates were broadly consistent with prior ecidence. A 2021 study reported mean EQ-5D utilities of 0.788 (SD, 0.233) for adults living with diabetes and 0.793 (SD, 0.223) for adults high blood pressure [43]. Using 2000 MEPS data, a 2005 analysis estimated a 0.073 utility decrement for severe obesity versus normal weight [44]. Earlier patient surveys from 2000 to 2001 found a mean EQ-5D of 0.80 among adults with diabetes [45], and a systematic review of stroke reported a pooled EQ-5D utility value of 0.68 (95% CI, 0.61–0.76) [46]. These comparisons support the face validity of our estimates, while differences across studies may reflect variation in study populations, survey years, disease definitions, and utility measurement methods.

Several limitations should be considered. First, EQ-5D utilities were mapped from SF-12 scores rather than directly measured. Although we used a validated mapping algorithm, mapped utilities may contain prediction error and may have a narrower distribution than directly measured EQ-5D values because mapping models predict the average EQ-5D value expected for a given SF-12 profile, which can pull very low or very high individual values toward the population mean. However, because the same SF-12 instrument and mapping algorithm were applied consistently across survey years, this limitation is unlikely to change the observed temporal trends direction. Second, the observational and repeated cross-sectional design precludes causal inference. The observed attenuation in condition-associated HRQoL decrements over time is consistent with improvements in treatment and risk-factor control, but may also reflect survivorship, shifting case mix, diagnostic reclassification, changes in healthcare access, health behaviors, or survey response patterns. Therefore, findings should be interpreted as population-level associations and temporal patterns rather than causal estimates. Third, the MEPS only includes a disease history indicator for broad "heart disease" category, combining coronary heart disease, angina, myocardial infarction, and other heart disease. Although each subtype differs in disease severity and clinical prognosis, we were not able to estimate separate results for each subtype as these indicators are clinically related and not mutually exclusive. For example, 36.9% of heart disease–positive respondents reported two or more subcategories. Modeling these subtypes separately would estimate the additional HRQoL burden of each subtype beyond co-occurring subtypes, rather than the overall burden associated with that diagnosis. Therefore, heart disease-associated HRQoL decrements should be interpreted as population-level aggregate estimates among those with any heart disease. Observed trends may also reflect changes in the composition of heart disease subtypes over time rather than uniform changes across all included conditions. Fourth, although we observed statistically significant attenuation in several disease-specific HRQoL decrements (e.g., 0.0007 HRQoL units increase per year for diabetes and heart disease), the annual change is small relative to a commonly cited threshold for clinically meaningful within-person change (0.03 utility units) [47]. To contextualize the potential relevance of these incremental shifts, we complemented trend estimates with a population-level illustration showing how modest per-person utility changes can accumulate into meaningful QALY differences when applied to highly prevalent, long-duration diseases (e.g., diabetes).

## Supplementary Information

Below is the link to the electronic supplementary material.

Supplementary Material 1

## Data Availability

The data used in this study are publicly available from the Medical Expenditure Panel Survey (MEPS), administered by the Agency for Healthcare Research and Quality (AHRQ).

## Declarations

### Preprint server

A previous version of this manuscript was posted on medRxiv as a preprint. The current manuscript has been revised in response to peer-review comments and differs from the posted preprint. DOI: 10.64898/2026.02.20.26346754.

## References

[1] Fontaine, K. (n.d.). *Arthritis and health-related quality of life.* Arthritis and health-related quality of life. Johns Hopkins Arthritis Center. Retrieved September 2, 2025 , from https://www.hopkinsarthritis.org/patient-corner/disease-management/quality-of-life-and-arthritis/?utm_source=chatgpt.com

[2] Testa, M. A., & Simonson, D. C. (1996). Assessment of quality-of-life outcomes. *The New England Journal of Medicine*, *334*(13), 835–840. 10.1056/NEJM199603283341306 8596551

[3] Herdman, M., Gudex, C., Lloyd, A., Janssen, M., Kind, P., Parkin, D., & Badia, X. (2011). Development and preliminary testing of the new five-level version of EQ-5D (EQ-5D-5L). *Quality of Life Research: An International Journal of Quality of Life Aspects of Treatment Care and Rehabilitation*, *20*(10), 1727–1736. 10.1007/s11136-011-9903-x 21479777 PMC3220807

[4] National Institute for Health and Care Excellence. (2022). 4 Economic evaluation. *In NICE health technology evaluations: The manual.* NICE.Retrieved September 26, 2025, from https://www.nice.org.uk/process/pmg36/chapter/economic-evaluation-2

[5] Devlin, P. D. N., Parkin, D., & Janssen, B. (2020). Analysis of EQ-5D values. In *Methods for Analysing and Reporting EQ-5D Data [Internet]*. Springer. 10.1007/978-3-030-47622-9_4 33347096

[6] Ahmad, F. B., Cisewski, J. A., & Anderson, R. N. (2024). Leading causes of death in the US, 2019–2023. *Journal Of The American Medical Association*, *332*(12), 957–958. 10.1001/jama.2024.15563 39116093 PMC11874302

[7] Grundy, S. M., Cleeman, J. I., Daniels, S. R., Donato, K. A., Eckel, R. H., Franklin,B. A., … Costa, F. (2005). Diagnosis and management of the metabolic syndrome. *Circulation*, *112*(17), e285–e290. 10.1161/CIRCULATIONAHA.105.169405. 16157765

[8] Joynt Maddox, K. E., Elkind, M. S. V., Aparicio, H. J., Commodore-Mensah, Y., de Ferranti, S. D., Dowd, W. N., … on behalf of the American Heart Association. (2024). Forecasting the burden of cardiovascular disease and stroke in the United States Through 2050—prevalence of risk factors and disease: A presidential advisory From the American Heart Association. *Circulation*, *150*(4), e65–e88. 10.1161/CIR.0000000000001256. 38832505

[9] Spertus, J. A., Winder, J. A., Dewhurst, T. A., Deyo, R. A., Prodzinski, J., McDonell, M., & Fihn, S. D. (1995). Development and evaluation of the Seattle Angina Questionnaire: A new functional status measure for coronary artery disease. *Journal of the American College of Cardiology*, *25*(2), 333–341. 10.1016/0735-1097(94)00397-9 7829785

[10] Rubin, R. R., & Peyrot, M. (1999). Quality of life and diabetes. *Diabetes/Metabolism Research and Reviews*, *15*(3), 205–218. 10441043 10.1002/(sici)1520-7560(199905/06)15:3<205::aid-dmrr29>3.0.co;2-o

[11] Schwimmer, J. B., Burwinkle, T. M., & Varni, J. W. (2003). Health-related quality of life of severely obese children and adolescents. *Journal Of The American Medical Association*, *289*(14), 1813–1819. 10.1001/jama.289.14.1813 12684360

[12] Piña, I. L., Camacho, A., Ibrahim, N. E., Felker, G. M., Butler, J., Maisel, A. S., & PROVE-HF Investigators. (2021). Improvement of health status following initiation of Sacubitril/Valsartan in heart failure and reduced ejection fraction. *JACC: Heart Failure*, *9*(1), 42–51. 10.1016/j.jchf.2020.09.012 33189630

[13] Weintraub, W. S., Spertus, J. A., Kolm, P., Maron, D. J., Zhang, Z., Jurkovitz, C.,… Boden, W. E. (2008). Effect of PCI on quality of life in patients with stable coronary disease. *New England Journal of Medicine*, *359*(7), 677–687. 10.1056/NEJMoa072771. 18703470

[14] Rubin, R. R., Wadden, T. A., Bahnson, J. L., Blackburn, G. L., Brancati, F. L., Bray,G. A., … Look AHEAD Research Group. (2014). Impact of intensive lifestyle intervention on depression and health-related quality of life in type 2 diabetes: the Look AHEAD Trial. *Diabetes Care*, *37*(6), 1544–1553. 10.2337/dc13-1928. PMC4030096 24855155

[15] Wilding, J. P. H., Batterham, R. L., Calanna, S., Davies, M., Gaal, L. F. V., Lingvay, I., & Kushner, R. F. (2021). Once-weekly semaglutide in adults with overweight or obesity. *New England Journal of Medicine*, *384*(11), 989–1002. 10.1056/NEJMoa2032183 33567185

[16] Forman, D. E., Maurer, M. S., Boyd, C., Brindis, R., Salive, M. E., Horne, F. M.,… Rich, M. W. (2018). Multimorbidity in older adults with cardiovascular disease.*Journal of the American College of Cardiology*, *71*(19), 2149–2161. 10.1016/j.jacc.2018.03.022. PMC6028235 29747836

[17] Wu, Z., Qian, F., Zou, S., Zou, X., Zhang, R., Guo, X., & Li, H. (2025). Trends and disparities in health status and health care in the United States during COVID-19 pandemic. *BMC Medicine*, *23*, 478. 10.1186/s12916-025-04315-4 40817069 PMC12357392

[18] Spertus, J. A., Jones, P. G., Sandhu, A. T., & Arnold, S. V. (2020). Interpreting the Kansas City Cardiomyopathy questionnaire in clinical trials and clinical care: JACC state-of-the-art review. *Journal of the American College of Cardiology*, *76*(20), 2379–2390. 10.1016/j.jacc.2020.09.542 33183512

[19] Thomas, M., Jones, P. G., Arnold, S. V., & Spertus, J. A. (2021). State of the art review: Interpreting the Seattle Angina Questionnaire as an outcome in clinical trials and in clinical care. *JAMA cardiology*, *6*(5), 593–599. 10.1001/jamacardio.2020.7478 33566062 PMC8651216

[20] Williams, L. S., Weinberger, M., Harris, L. E., Clark, D. O., & Biller, J. (1999). Development of a stroke-specific quality of Life Scale. *Stroke*, *30*(7), 1362–1369. 10.1161/01.STR.30.7.1362 10390308

[21] Bradley, C., Todd, C., Gorton, T., Symonds, E., Martin, A., & Plowright, R. (1999). The development of an individualized questionnaire measure of perceived impact of diabetes on quality of life: the ADDQoL. *Quality of Life Research: An International Journal of Quality of Life Aspects of Treatment Care and Rehabilitation*, *8*(1–2), 79–91. 10.1023/a:1026485130100 10457741

[22] Kolotkin, R. L., Crosby, R. D., Kosloski, K. D., & Williams, G. R. (2001). Development of a brief measure to assess quality of life in obesity. *Obesity Research*, *9*(2), 102–111. 10.1038/oby.2001.13 11316344

[23] Sullivan, P. W., & Ghushchyan, V. (2006). Preference-based EQ-5D index scores for chronic conditions in the United States. *Medical decision making: an international journal of the Society for Medical Decision Making*, *26*(4), 410–420. 10.1177/0272989X06290495 16855129 PMC2634296

[24] Lawrence, W. F., & Fleishman, J. A. (2004). Predicting EuroQoL EQ-5D preference scores from the SF-12 health survey in a nationally representative sample. *Medical Decision Making: An International Journal of the Society for Medical Decision Making*, *24*(2), 160–169. 10.1177/0272989X04264015 15090102

[25] Maheswaran, H., Petrou, S., Rees, K., & Stranges, S. (2013). Estimating EQ-5D utility values for major health behavioural risk factors in England. *Journal of Epidemiology and Community Health*, *67*(2), 172–180. 10.1136/jech-2012-201019 22844084

[26] Agency for Healthcare Research and Quality. (n.d.). *Medical Expenditure Panel Survey public use file search results. *Retrieved September 2, 2025, from https://meps.ahrq.gov/mepsweb/

[27] Nuttall, F. Q. (2015). Body mass index. *Nutrition Today*, *50*(3), 117–128. 10.1097/NT.0000000000000092 27340299 PMC4890841

[28] Sullivan, P. W., & Ghushchyan, V. (2006). Mapping the EQ-5D index from the SF-12: US general population preferences in a nationally representative sample. *Medical Decision Making: An International Journal of the Society for Medical Decision Making*, *26*(4), 401–409. 10.1177/0272989X06290496 16855128 PMC2713176

[29] Apple, R., Samuels, L. R., Fonnesbeck, C., Schlundt, D., Mulvaney, S., Hargreaves, M., & Heerman, W. J. (2018). Body mass index and health-related quality of life. *Obesity Science & Practice*, *4*(5), 417–426. 10.1002/osp4.292 30338112 PMC6180707

[30] DerSimonian, R., & Laird, N. (1986). Meta-analysis in clinical trials. *Controlled Clinical Trials*, *7*(3), 177–188. 10.1016/0197-2456(86)90046-2 3802833

[31] Stommel, M., & Schoenborn, C. A. (2009). Accuracy and usefulness of BMI measures based on self-reported weight and height: Findings from the NHANES & NHIS 2001–2006. *BMC public health*, *9*, 421. 10.1186/1471-2458-9-421 19922675 PMC2784464

[32] Jayedi, A., Soltani, S., Motlagh, S. Z., Emadi, A., Shahinfar, H., Moosavi, H., & Shab-Bidar, S. (2022). Anthropometric and adiposity indicators and risk of type 2 diabetes: systematic review and dose-response meta-analysis of cohort studies. 10.1136/bmj-2021-067516 PMC8764578 35042741

[33] Mahdi, S., Marr, C., Buckland, N. J., & Chilcott, J. (2022). Methods for the economic evaluation of obesity prevention dietary interventions in children: A systematic review and critical appraisal of the evidence. *Obesity Reviews*, *23*(9), e13457. 10.1111/obr.13457 35478373 PMC9542346

[34] Lubetkin, E. I., Jia, H., Franks, P., & Gold, M. R. (2005). Relationship among sociodemographic factors, clinical conditions, and health-related quality of life: examining the EQ-5D in the U.S. general population. *Quality of Life Research*, *14*(10), 2187–2196. 10.1007/s11136-005-8028-5 16328899

[35] Fang, M. (2020). Trends in diabetes management among US adults: 1999–2016. *Journal of General Internal Medicine*, *35*(5), 1427–1434. 10.1007/s11606-019-05587-2 31898135 PMC7210372

[36] Gregg, E. W., Li, Y., Wang, J., Burrows, N. R., Ali, M. K., Rolka, D., & Geiss, L. (2014). Changes in diabetes-related complications in the United States, 1990–2010. *The New England Journal of Medicine*, *370*(16), 1514–1523. 10.1056/NEJMoa1310799 24738668

[37] Campbell, J. A., Bishu, K. G., Walker, R. J., & Egede, L. E. (2017). Trends of medical expenditures and quality of life in US adults with diabetes: the medical expenditure panel survey, 2002–2011. *Health and Quality of Life Outcomes*, *15*(1), 70. 10.1186/s12955-017-0651-7 28407776 PMC5390377

[38] Will, J. C., Yuan, K., & Ford, E. (2014). National trends in the prevalence and medical history of Angina: 1988 to 2012. *Circulation: Cardiovascular Quality and Outcomes*, *7*(3), 407–413. 10.1161/CIRCOUTCOMES.113.000779 24847083 PMC4366681

[39] Hardy, S. T., Jaeger, B. C., Foti, K., Ghazi, L., Wozniak, G., & Muntner, P. (2024). Trends in blood pressure control among US adults with hypertension, 2013–2014 to 2021–2023. *American Journal of Hypertension*, *38*(2), 120–128. 10.1093/ajh/hpae141 PMC11735471 39504487

[40] Whelton, P. K., Carey, R. M., Aronow, W. S., Casey, D. E., Collins, K. J., Dennison Himmelfarb, C., … Wright, J. T. (2018). 2017 ACC/AHA/AAPA/ABC/ACPM/AGS/APhA/ASH/ASPC/NMA/PCNA guideline for the prevention, detection, evaluation, and management of high blood pressure in adults: A report of the American college of cardiology/American heart association task force on clinical practice guidelines. *Hypertension*, *71*(6), e13–e115. 10.1161/HYP.0000000000000065. 29133356

[41] Centers for Disease Control and Prevention. (2024). *Adult obesity facts.* Retrieved February 9, 2026, from https://www.cdc.gov/obesity/adult-obesity-facts/index.html

[42] Hales, C. M., Carroll, M. D., Fryar, C. D., & Ogden, C. L. (2020). Prevalence of obesity and severe obesity among adults: United States, 2017–2018. *NCHS Data Brief*, (360), 1–8. https://www.cdc.gov/nchs/products/databriefs/db360.htm 32487284

[43] Jiang, R., Janssen, M. F. B., & Pickard, A. S. (2021). US population norms for the EQ-5D-5L and comparison of norms from face-to-face and online samples. *Quality of Life Research*, *30*(3), 803–816. 10.1007/s11136-020-02650-y 33025373 PMC7952367

[44] Jia, H., & Lubetkin, E. I. (2005). The impact of obesity on health-related quality-of-life in the general adult US population. *Journal of Public Health (Oxford England)*, *27*(2), 156–164. 10.1093/pubmed/fdi025 15820993

[45] Zhang, P., Brown, M. B., Bilik, D., Ackermann, R. T., Li, R., & Herman, W. H. (2012). Health utility scores for people with type 2 diabetes in U.S. managed care health plans. *Diabetes Care*, *35*(11), 2250–2256. 10.2337/dc11-2478 22837369 PMC3476906

[46] Joundi, R. A., Adekanye, J., Leung, A. A., Ronksley, P., Smith, E. E., Rebchuk, A. D., & Bresee, L. C. (2022). Health state utility values in people with stroke: A systematic review and meta-analysis. *Journal of the American Heart Association*. 10.1161/JAHA.121.024296 35730598 PMC9333363

[47] Crosby, R. D., Kolotkin, R. L., & Williams, G. R. (2003). Defining clinically meaningful change in health-related quality of life. *Journal of Clinical Epidemiology*, *56*(5), 395–407. 10.1016/S0895-4356(03)00044-1 12812812

