# Supplementary material for: Trends in cardiometabolic disease and health-related quality of life in the United States, 2001–2022
Source: Qual Life Res. 2026 Jun 15;35(8):195. doi: 10.1007/s11136-026-04300-1 (PMC13269321; doi:10.1007/s11136-026-04300-1)
Supplement: Supplementary file 1 — Supplementary Material 1 [file 11136_2026_4300_MOESM1_ESM.docx]

**Trends in Cardiometabolic Disease and Health-Related Quality of Life in the United States, 2001-2022**

*Quality of Life Research*

Danwei Yang^1^, *David D. Kim^1,2^

^1^Department of Public Health Sciences, University of Chicago, Chicago, IL, United States

^2^Department of Medicine, University of Chicago, Chicago, IL, United States

*David.Kim@bsd.uchicago.edu

**Supplementary Material**

Table of Contents

[Section I. Model Specification and Fit Diagnostics 1](#_Toc229759614)

[Table S1. Generalized Variance Inflation Factors for Fully Adjusted Model (Model 4), MEPS 2022 (n = 11,275) 1](#_Toc229759615)

[Table S2. Akaike Information Criterion (AIC) by Model Specification and Survey Year, MEPS 2001–2022 1](#_Toc229759616)

[Section II. Primary Analysis: Subgroup Trends and Year-Specific Regression Results 1](#_Toc229759617)

[Figure S1. EQ-5D Utility Trends by Sex, MEPS 2001–2022 1](#_Toc229759618)

[Figure S2. EQ-5D Utility Trends by Race/Ethnicity, MEPS 2001–2022 1](#_Toc229759619)

[Figure S3. EQ-5D Utility Trends by Education Level, MEPS 2001–2022 2](#_Toc229759620)

[Figure S4. EQ-5D Utility Trends by Income Level, MEPS 2001–2022 2](#_Toc229759621)

[Table S3. Survey-Weighted Linear Regression of EQ-5D Utility on Cardiometabolic Conditions and Covariates, MEPS 2022 3](#_Toc229759622)

[Table S4. Survey-Weighted Linear Regression of EQ-5D Utility on Cardiometabolic Conditions and Covariates, MEPS 2020 4](#_Toc229759623)

[Table S5. Survey-Weighted Linear Regression of EQ-5D Utility on Cardiometabolic Conditions and Covariates, MEPS 2018 5](#_Toc229759624)

[Table S6. Survey-Weighted Linear Regression of EQ-5D Utility on Cardiometabolic Conditions and Covariates, MEPS 2016 6](#_Toc229759625)

[Table S7. Survey-Weighted Linear Regression of EQ-5D Utility on Cardiometabolic Conditions and Covariates, MEPS 2015 7](#_Toc229759626)

[Table S8. Survey-Weighted Linear Regression of EQ-5D Utility on Cardiometabolic Conditions and Covariates, MEPS 2014 8](#_Toc229759627)

[Table S9. Survey-Weighted Linear Regression of EQ-5D Utility on Cardiometabolic Conditions and Covariates, MEPS 2013 9](#_Toc229759628)

[Table S10. Survey-Weighted Linear Regression of EQ-5D Utility on Cardiometabolic Conditions and Covariates, MEPS 2012 10](#_Toc229759629)

[Table S11. Survey-Weighted Linear Regression of EQ-5D Utility on Cardiometabolic Conditions and Covariates, MEPS 2011 11](#_Toc229759630)

[Table S12. Survey-Weighted Linear Regression of EQ-5D Utility on Cardiometabolic Conditions and Covariates, MEPS 2010 12](#_Toc229759631)

[Table S13. Survey-Weighted Linear Regression of EQ-5D Utility on Cardiometabolic Conditions and Covariates, MEPS 2009 13](#_Toc229759632)

[Table S14. Survey-Weighted Linear Regression of EQ-5D Utility on Cardiometabolic Conditions and Covariates, MEPS 2008 14](#_Toc229759633)

[Table S15. Survey-Weighted Linear Regression of EQ-5D Utility on Cardiometabolic Conditions and Covariates, MEPS 2007 15](#_Toc229759634)

[Table S16. Survey-Weighted Linear Regression of EQ-5D Utility on Cardiometabolic Conditions and Covariates, MEPS 2006 16](#_Toc229759635)

[Table S17. Survey-Weighted Linear Regression of EQ-5D Utility on Cardiometabolic Conditions and Covariates, MEPS 2005 17](#_Toc229759636)

[Table S18. Survey-Weighted Linear Regression of EQ-5D Utility on Cardiometabolic Conditions and Covariates, MEPS 2004 18](#_Toc229759637)

[Table S19. Survey-Weighted Linear Regression of EQ-5D Utility on Cardiometabolic Conditions and Covariates, MEPS 2003 19](#_Toc229759638)

[Table S20. Survey-Weighted Linear Regression of EQ-5D Utility on Cardiometabolic Conditions and Covariates, MEPS 2002 20](#_Toc229759639)

[Table S21. Survey-Weighted Linear Regression of EQ-5D Utility on Cardiometabolic Conditions and Covariates, MEPS 2001 21](#_Toc229759640)

[Table S22. Meta-Regression of Disease-Specific EQ-5D Trends: Linear Specification, MEPS 2001–2022 22](#_Toc229759641)

[Table S23. Meta-Regression of Disease-Specific EQ-5D Trends: Quadratic Specification, MEPS 2001–2022 22](#_Toc229759642)

[Section III. Sensitivity Analyses 23](#_Toc229759643)

[Table S24. Survey-Weighted Linear Regression of EQ-5D Utility Including COVID-19 Pandemic Year, MEPS 2015, 2016, 2018, 2020, and 2022 23](#_Toc229759644)

[Table S25. Annual High Blood Pressure Associated EQ-5D Decrement Across All Survey Years, MEPS 2001–2022 24](#_Toc229759645)

[Table S26. Meta-Regression Test for High Blood Pressure Associated EQ-5D Trends in BMI-Missing Cycles 24](#_Toc229759646)

[Table S27. Full Model Comparison: Survey-Weighted Linear Regression of EQ-5D Utility With and Without Ceiling Observations (EQ-5D = 1.0), Pooled MEPS 2015, 2016, 2018, and 2022 (Model 4, Fully Adjusted) 25](#_Toc229759647)

[Table S28. Comparison of DerSimonian–Laird and Continuous-Time AR(1) Meta-Regression Estimates for Disease-Specific HRQoL Trends 26](#_Toc229759648)

[Table S29. Survey-Weighted Linear Regression of EQ-5D Utility Using Self-Reported Versus Corrected BMI, Pooled MEPS 2015, 2016, 2018, and 2022 (Model 4, Fully Adjusted) 26](#_Toc229759649)

# **Section I. Model Specification and Fit Diagnostics**

## Table S1. Generalized Variance Inflation Factors for Fully Adjusted Model (Model 4), MEPS 2022 (n = 11,275)

| **Variable** | **GVIF** | **df** | **GVIF¹⁽²·df⁾** | **Adjusted VIF** |
| --- | --- | --- | --- | --- |
| Age group | 1.724 | 6 | 1.046 | 1.095 |
| Sex | 1.039 | 1 | 1.019 | 1.039 |
| Race/ethnicity | 1.371 | 4 | 1.040 | 1.082 |
| Income level | 1.391 | 4 | 1.042 | 1.086 |
| Education level | 1.459 | 4 | 1.048 | 1.099 |
| Type 2 diabetes | 1.215 | 1 | 1.102 | 1.215 |
| Heart disease | 1.185 | 1 | 1.089 | 1.185 |
| High blood pressure | 1.554 | 1 | 1.247 | 1.554 |
| High cholesterol | 1.412 | 1 | 1.188 | 1.412 |
| Stroke | 1.086 | 1 | 1.042 | 1.086 |
| Obesity (BMI ≥ 30) | 2.828 | 1 | 1.682 | 2.828 |
| BMI − 25, linear | 5.722 | 1 | 2.392 | 5.722 |
| (BMI − 25)², quadratic | 3.174 | 1 | 1.782 | 3.174 |

*GVIF = Generalized Variance Inflation Factor (Fox & Monette, 1992). For categorical predictors with df > 1, GVIF¹⁽²·df⁾ is used for comparability with the standard VIF threshold. Adjusted VIF = [GVIF¹⁽²·df⁾]²..*

## Table S2. Akaike Information Criterion (AIC) by Model Specification and Survey Year, MEPS 2001–2022

| Survey Year | Model 1 | Model 2 | Model 3 | Model 4 | ΔAIC  M2 vs M1 | ΔAIC  M3 vs M1 | ΔAIC  M4 vs M1 | ΔAIC  M4 vs M2 | Best Model |
| --- | --- | --- | --- | --- | --- | --- | --- | --- | --- |
| 2001 | -33308 | -35247 | -33416 | -35361 | -1939 | -108 | -2053 | -114 | M4 |
| 2002 | -38104 | -40386 | -38207 | -40429 | -2281 | -103 | -2325 | -44 | M4 |
| 2003 | -32660 | -34710 | -32833 | -34764 | -2050 | -173 | -2104 | -54 | M4 |
| 2004 | -32280 | -34241 | -32371 | -34290 | -1961 | -92 | -2010 | -49 | M4 |
| 2005 | -32140 | -34471 | -32415 | -34565 | -2331 | -275 | -2425 | -94 | M4 |
| 2006 | -32777 | -35073 | -33037 | -35167 | -2297 | -260 | -2390 | -93 | M4 |
| 2007 | -29867 | -32157 | -30077 | -32257 | -2289 | -209 | -2389 | -100 | M4 |
| 2008 | -31334 | -34154 | -32003 | -34446 | -2820 | -670 | -3112 | -292 | M4 |
| 2009 | -35167 | -37991 | -35613 | -38154 | -2825 | -446 | -2988 | -163 | M4 |
| 2010 | -31258 | -33729 | -31675 | -33934 | -2471 | -417 | -2676 | -205 | M4 |
| 2011 | -33666 | -36246 | -34063 | -36495 | -2579 | -397 | -2829 | -250 | M4 |
| 2012 | -18975 | -20312 | -19176 | -20364 | -1337 | -201 | -1390 | -53 | M4 |
| 2013 | -35029 | -37732 | -35461 | -37895 | -2703 | -432 | -2866 | -163 | M4 |
| 2014 | -33233 | -36014 | -33514 | -36034 | -2781 | -281 | -2801 | -20 | M4 |
| 2015 | -33941 | -37052 | -34432 | -37127 | -3111 | -491 | -3185 | -74 | M4 |
| 2016 | -34133 | -37360 | -34727 | -37565 | -3227 | -594 | -3432 | -205 | M4 |
| 2018 | -31842 | -33053 | -31069 | -33367 | -1211 | 773 | -1525 | -314 | M4 |
| 2020 | -22843 | -24778 | -23176 | -24927 | -1935 | -333 | -2084 | -149 | M4 |
| 2022 | -18324 | -19035 | -18094 | -19219 | -712 | 230 | -896 | -184 | M4 |

*Model 1: Sociodemographic covariates only (age group, sex, race/ethnicity, education, income).*

*Model 2: Model 1 + cardiometabolic conditions (diabetes, heart disease, high blood pressure, high cholesterol, stroke, obesity)*

*Model 3: Model 1 + BMI (centered at 25 kg/m²) + BMI²*

*Model 4: Model 1 + cardiometabolic conditions + BMI + BMI² (fully adjusted)*

*AIC computed via the quasi-likelihood approximation for survey-weighted regression. AIC values are valid for within-year model comparisons only and should not be compared across years.*

# **Section II. Primary Analysis: Subgroup Trends and Year-Specific Regression Results**

## Figure S1. EQ-5D Utility Trends by Sex, MEPS 2001–2022


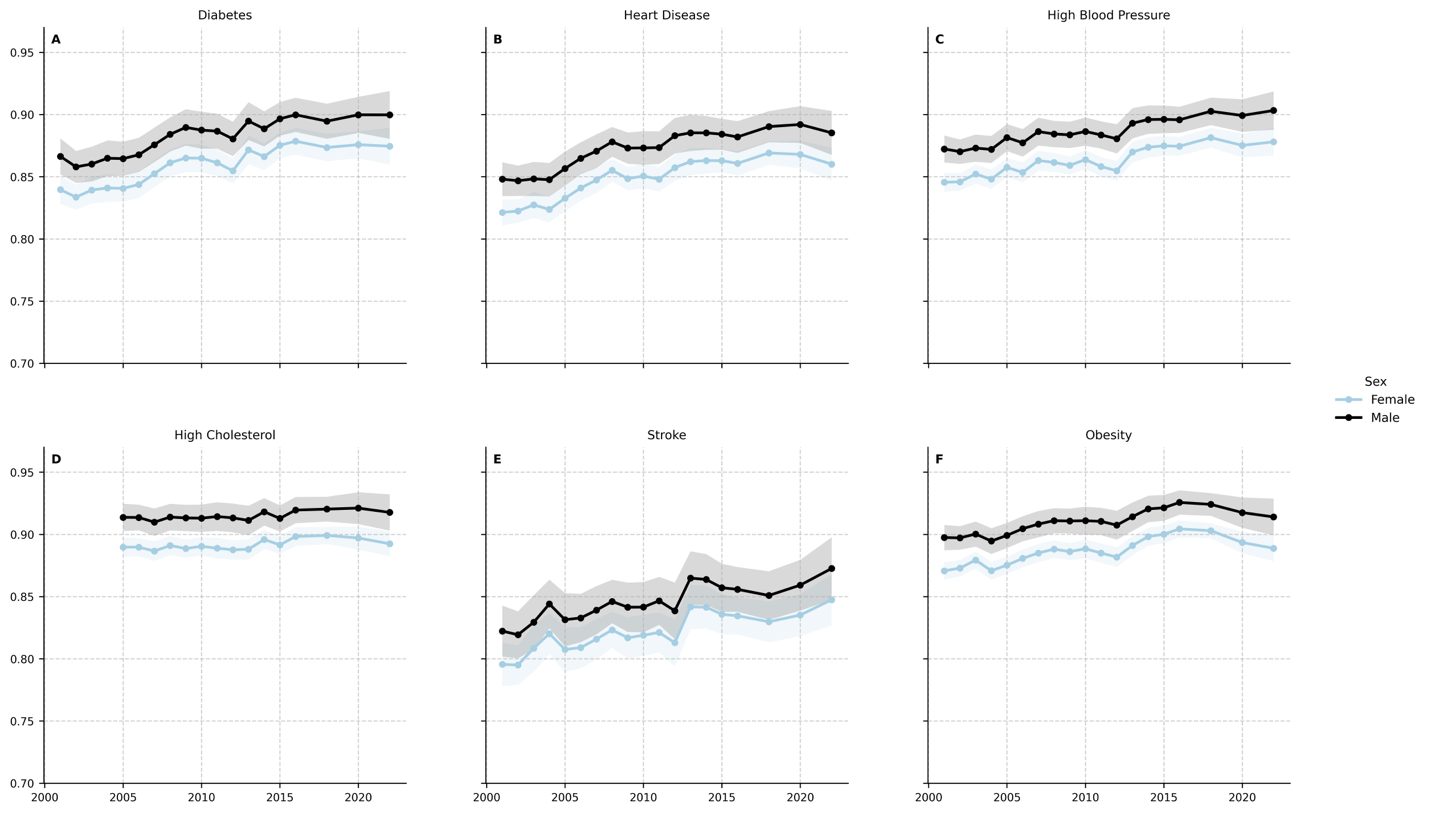


## Figure S2. EQ-5D Utility Trends by Race/Ethnicity, MEPS 2001–2022


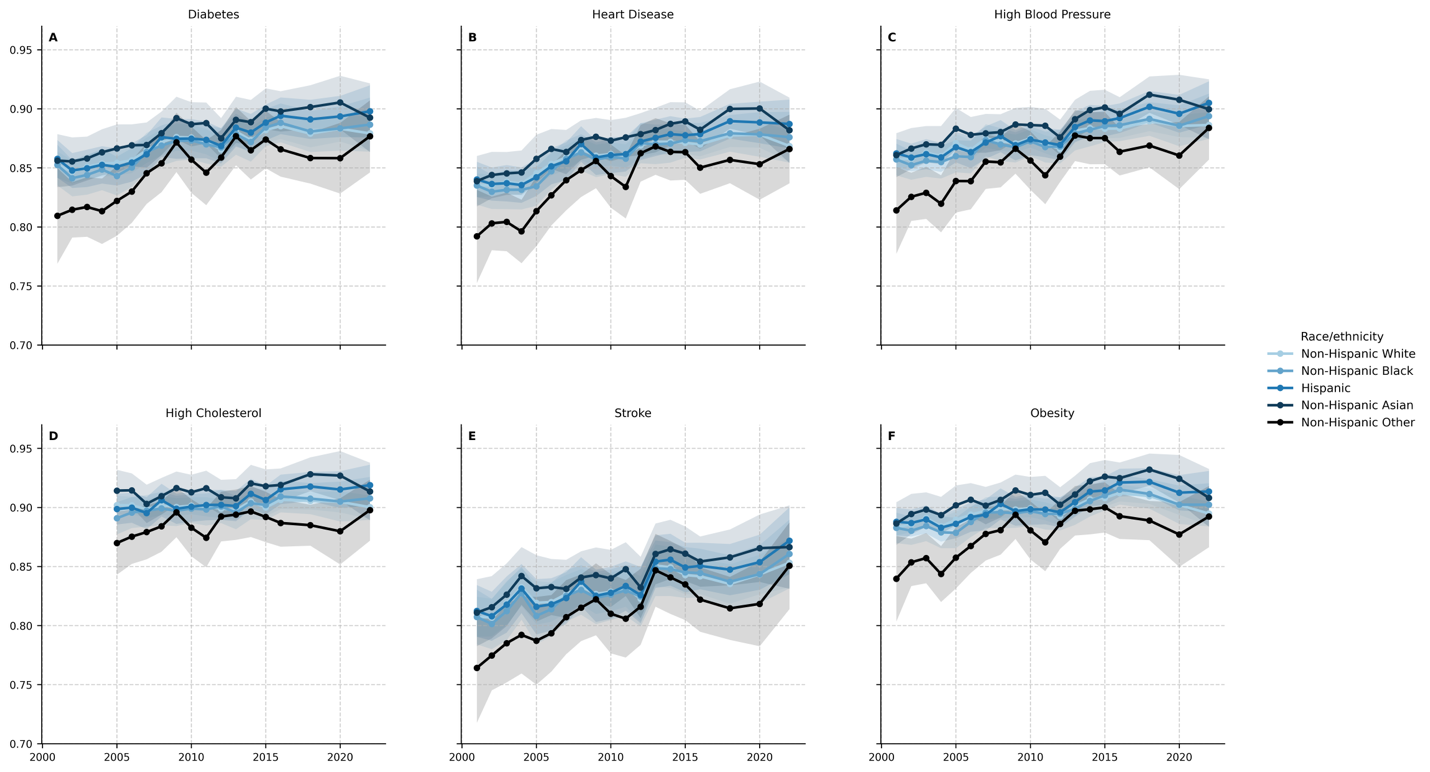


## Figure S3. EQ-5D Utility Trends by Education Level, MEPS 2001–2022


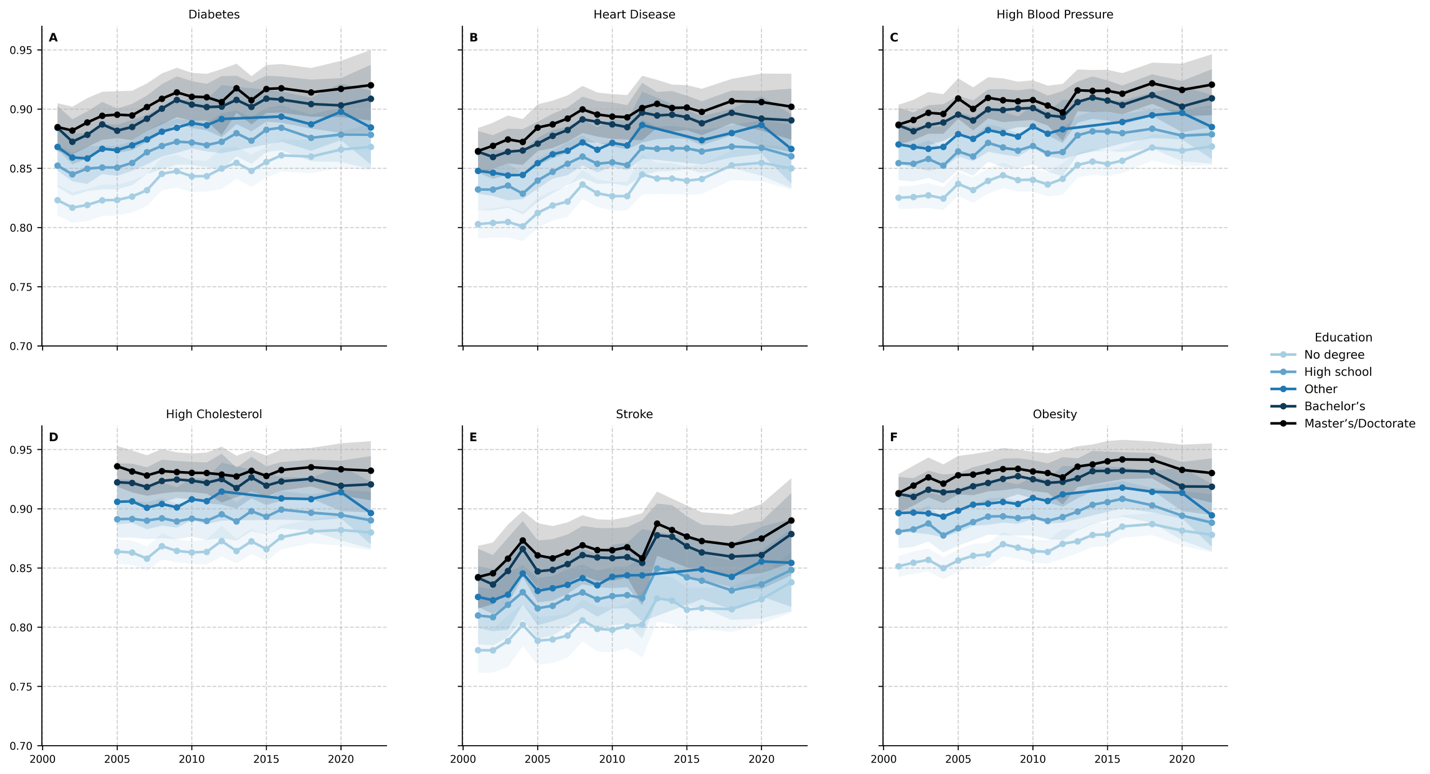


## Figure S4. EQ-5D Utility Trends by Income Level, MEPS 2001–2022


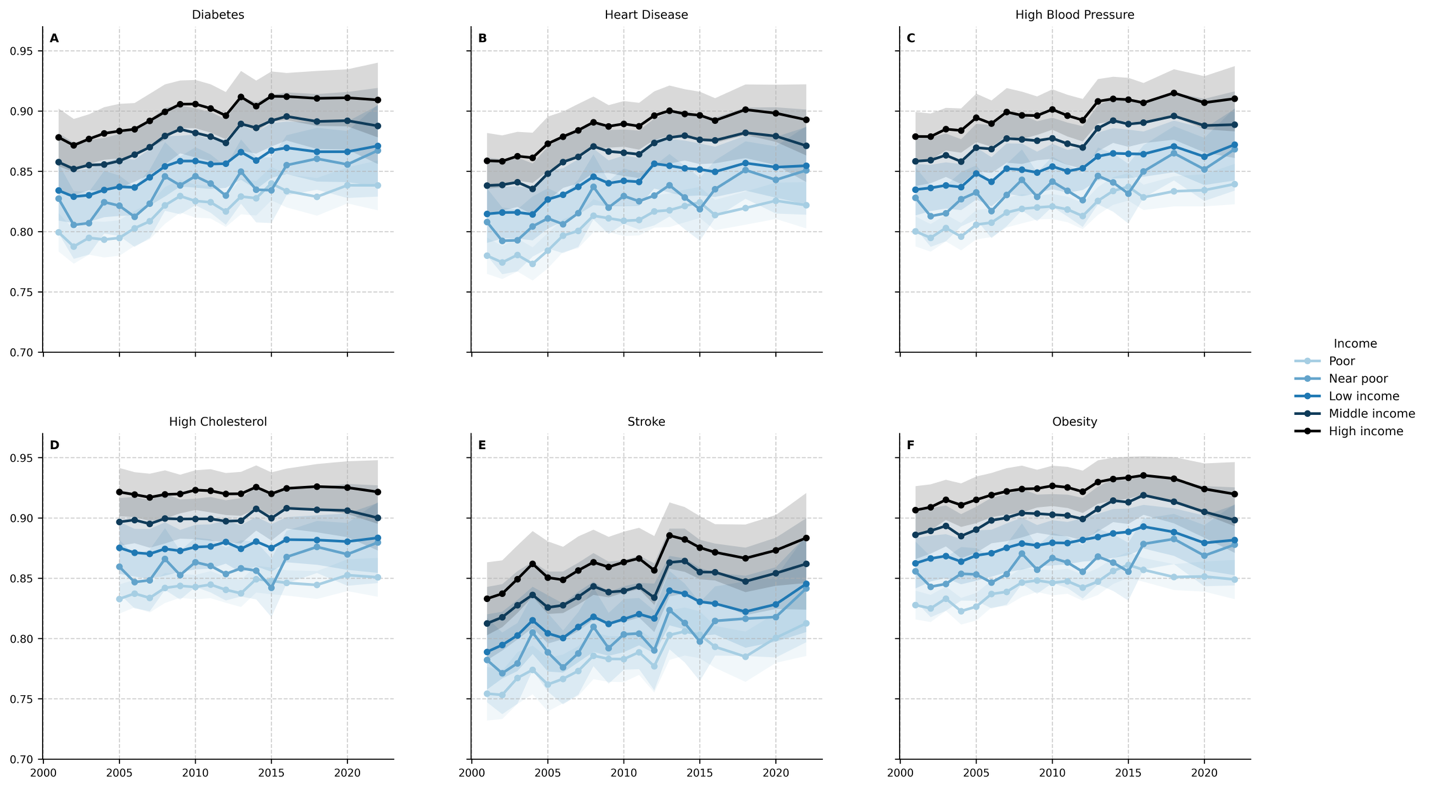


## Table S3. Survey-Weighted Linear Regression of EQ-5D Utility on Cardiometabolic Conditions and Covariates, MEPS 2022

|  | Model 1: Sociodemographic | | | Model 2: Sociodemographic + CMD | | | Model 3: Sociodemographic + BMI (centered at 25) | | | Model 4: Sociodemographic + CMD + BMI (centered at 25) | | |
| --- | --- | --- | --- | --- | --- | --- | --- | --- | --- | --- | --- | --- |
| Variable | Beta | (SE) | p | Beta | (SE) | p | Beta | (SE) | p | Beta | (SE) | p |
| Intercept | 0.8419 | (0.0074) | <0.0001 | 0.8624 | (0.0069) | <0.0001 | 0.8539 | (0.0070) | <0.0001 | 0.8670 | (0.0069) | <0.0001 |
| *Age group (ref: 18-29 years)* | | | | | | | | | | | | |
| 30-39 | -0.0189 | (0.0044) | <0.0001 | -0.0087 | (0.0040) | 0.0326 | -0.0122 | (0.0042) | 0.0040 | -0.0091 | (0.0040) | 0.0238 |
| 40-49 | -0.0390 | (0.0046) | <0.0001 | -0.0176 | (0.0045) | <0.001 | -0.0294 | (0.0048) | <0.0001 | -0.0184 | (0.0045) | <0.0001 |
| 50-59 | -0.0650 | (0.0047) | <0.0001 | -0.0281 | (0.0046) | <0.0001 | -0.0565 | (0.0047) | <0.0001 | -0.0303 | (0.0046) | <0.0001 |
| 60-69 | -0.0795 | (0.0047) | <0.0001 | -0.0250 | (0.0047) | <0.0001 | -0.0719 | (0.0047) | <0.0001 | -0.0288 | (0.0047) | <0.0001 |
| 70-79 | -0.1040 | (0.0056) | <0.0001 | -0.0353 | (0.0053) | <0.0001 | -0.0981 | (0.0055) | <0.0001 | -0.0402 | (0.0053) | <0.0001 |
| 80+ | -0.1470 | (0.0067) | <0.0001 | -0.0774 | (0.0068) | <0.0001 | -0.1512 | (0.0068) | <0.0001 | -0.0831 | (0.0068) | <0.0001 |
| *Sex (ref: Female)* | | | | | | | | | | | | |
| Male | 0.0186 | (0.0022) | <0.0001 | 0.0214 | (0.0022) | <0.0001 | 0.0153 | (0.0022) | <0.0001 | 0.0191 | (0.0021) | <0.0001 |
| *Race/Ethnicity (ref: Non-Hispanic White)* | | | | | | | | | | | | |
| Non-Hispanic Black | 0.0113 | (0.0046) | 0.0139 | 0.0183 | (0.0045) | <0.0001 | 0.0196 | (0.0045) | <0.0001 | 0.0193 | (0.0044) | <0.0001 |
| Hispanic | 0.0369 | (0.0043) | <0.0001 | 0.0304 | (0.0040) | <0.0001 | 0.0326 | (0.0040) | <0.0001 | 0.0289 | (0.0039) | <0.0001 |
| Non-Hispanic Asian/Pacific Islander | 0.0125 | (0.0065) | 0.0551 | 0.0057 | (0.0055) | 0.3009 | 0.0042 | (0.0056) | 0.4512 | 0.0046 | (0.0055) | 0.3982 |
| Non-Hispanic Other | -0.0033 | (0.0076) | 0.6656 | 0.0015 | (0.0076) | 0.8475 | -0.0030 | (0.0072) | 0.6770 | 0.0013 | (0.0072) | 0.8556 |
| *Education (ref: No degree)* | | | | | | | | | | | | |
| High school diploma / GED | 0.0051 | (0.0049) | 0.3005 | 0.0080 | (0.0048) | 0.0934 | 0.0079 | (0.0051) | 0.1210 | 0.0079 | (0.0048) | 0.1036 |
| Other post-secondary degree | 0.0113 | (0.0065) | 0.0812 | 0.0141 | (0.0061) | 0.0222 | 0.0134 | (0.0066) | 0.0428 | 0.0133 | (0.0062) | 0.0326 |
| Bachelor's degree | 0.0349 | (0.0056) | <0.0001 | 0.0325 | (0.0053) | <0.0001 | 0.0349 | (0.0055) | <0.0001 | 0.0321 | (0.0052) | <0.0001 |
| Master's or Doctorate degree | 0.0504 | (0.0060) | <0.0001 | 0.0444 | (0.0056) | <0.0001 | 0.0473 | (0.0059) | <0.0001 | 0.0432 | (0.0056) | <0.0001 |
| *Income (% poverty level, ref: Poor <100%)* | | | | | | | | | | | | |
| Near poor (100%-124%) | 0.0335 | (0.0098) | <0.001 | 0.0280 | (0.0090) | 0.0021 | 0.0311 | (0.0098) | 0.0017 | 0.0288 | (0.0088) | 0.0012 |
| Low income (125%-199%) | 0.0374 | (0.0071) | <0.0001 | 0.0331 | (0.0065) | <0.0001 | 0.0373 | (0.0067) | <0.0001 | 0.0337 | (0.0064) | <0.0001 |
| Middle income (200%-399%) | 0.0526 | (0.0062) | <0.0001 | 0.0458 | (0.0057) | <0.0001 | 0.0530 | (0.0061) | <0.0001 | 0.0458 | (0.0057) | <0.0001 |
| High income (≥400%) | 0.0776 | (0.0060) | <0.0001 | 0.0643 | (0.0054) | <0.0001 | 0.0739 | (0.0057) | <0.0001 | 0.0634 | (0.0054) | <0.0001 |
| *Cardiometabolic Conditions* | | | | | | | | | | | | |
| Diabetes |  |  |  | -0.0414 | (0.0052) | <0.0001 |  |  |  | -0.0393 | (0.0052) | <0.0001 |
| Heart Disease |  |  |  | -0.0487 | (0.0043) | <0.0001 |  |  |  | -0.0479 | (0.0042) | <0.0001 |
| High Blood Pressure |  |  |  | -0.0313 | (0.0035) | <0.0001 |  |  |  | -0.0286 | (0.0035) | <0.0001 |
| High Cholesterol |  |  |  | -0.0227 | (0.0031) | <0.0001 |  |  |  | -0.0223 | (0.0031) | <0.0001 |
| Stroke |  |  |  | -0.0562 | (0.0081) | <0.0001 |  |  |  | -0.0568 | (0.0082) | <0.0001 |
| Obesity |  |  |  | -0.0370 | (0.0029) | <0.0001 |  |  |  | -0.0162 | (0.0044) | <0.001 |
| *Body Mass Index* | | | | | | | | | | | | |
| BMI (linear, centered at 25) |  |  |  |  |  |  | -0.0023 | (0.0003) | <0.0001 | -0.0002 | (0.0004) | 0.6607 |
| BMI² (quadratic) |  |  |  |  |  |  | -0.0001 | (0.0000) | <0.0001 | -0.0001 | (0.0000) | <0.0001 |

## Table S4. Survey-Weighted Linear Regression of EQ-5D Utility on Cardiometabolic Conditions and Covariates, MEPS 2020

|  | Model 1: Sociodemographic | | | Model 2: Sociodemographic + CMD | | | Model 3: Sociodemographic + BMI (centered at 25) | | | Model 4: Sociodemographic + CMD + BMI (centered at 25) | | |
| --- | --- | --- | --- | --- | --- | --- | --- | --- | --- | --- | --- | --- |
| Variable | Beta | (SE) | p | Beta | (SE) | p | Beta | (SE) | p | Beta | (SE) | p |
| Intercept | 0.8457 | (0.0053) | <0.0001 | 0.8693 | (0.0048) | <0.0001 | 0.8583 | (0.0053) | <0.0001 | 0.8722 | (0.0048) | <0.0001 |
| Age group (ref: 18-29 years) | | | | | | | | | | | | |
| 30-39 | -0.0201 | (0.0036) | <0.0001 | -0.0071 | (0.0033) | 0.0323 | -0.0104 | (0.0036) | 0.0039 | -0.0056 | (0.0033) | 0.0886 |
| 40-49 | -0.0432 | (0.0041) | <0.0001 | -0.0179 | (0.0037) | <0.0001 | -0.0329 | (0.0040) | <0.0001 | -0.0173 | (0.0036) | <0.0001 |
| 50-59 | -0.0669 | (0.0038) | <0.0001 | -0.0273 | (0.0035) | <0.0001 | -0.0579 | (0.0037) | <0.0001 | -0.0283 | (0.0035) | <0.0001 |
| 60-69 | -0.0876 | (0.0041) | <0.0001 | -0.0299 | (0.0041) | <0.0001 | -0.0795 | (0.0041) | <0.0001 | -0.0316 | (0.0041) | <0.0001 |
| 70-79 | -0.1055 | (0.0046) | <0.0001 | -0.0318 | (0.0044) | <0.0001 | -0.1003 | (0.0045) | <0.0001 | -0.0352 | (0.0044) | <0.0001 |
| 80+ | -0.1594 | (0.0059) | <0.0001 | -0.0802 | (0.0063) | <0.0001 | -0.1599 | (0.0061) | <0.0001 | -0.0834 | (0.0063) | <0.0001 |
| *Sex (ref: Female)* | | | | | | | | | | | | |
| Male | 0.0157 | (0.0020) | <0.0001 | 0.0201 | (0.0017) | <0.0001 | 0.0129 | (0.0019) | <0.0001 | 0.0183 | (0.0018) | <0.0001 |
| *Race/Ethnicity (ref: Non-Hispanic White)* | | | | | | | | | | | | |
| Non-Hispanic Black | 0.0033 | (0.0041) | 0.4196 | 0.0087 | (0.0040) | 0.0300 | 0.0077 | (0.0042) | 0.0654 | 0.0093 | (0.0040) | 0.0211 |
| Hispanic | 0.0264 | (0.0037) | <0.0001 | 0.0229 | (0.0032) | <0.0001 | 0.0252 | (0.0033) | <0.0001 | 0.0217 | (0.0031) | <0.0001 |
| Non-Hispanic Asian/Pacific Islander | 0.0256 | (0.0059) | <0.0001 | 0.0172 | (0.0052) | 0.0012 | 0.0161 | (0.0052) | 0.0024 | 0.0164 | (0.0052) | 0.0020 |
| Non-Hispanic Other | -0.0321 | (0.0089) | <0.001 | -0.0212 | (0.0089) | 0.0184 | -0.0265 | (0.0097) | 0.0069 | -0.0196 | (0.0091) | 0.0325 |
| *Education (ref: No degree)* | | | | | | | | | | | | |
| High school diploma / GED | 0.0104 | (0.0041) | 0.0116 | 0.0096 | (0.0041) | 0.0208 | 0.0127 | (0.0041) | 0.0025 | 0.0099 | (0.0040) | 0.0147 |
| Other post-secondary degree | 0.0260 | (0.0049) | <0.0001 | 0.0249 | (0.0046) | <0.0001 | 0.0280 | (0.0048) | <0.0001 | 0.0248 | (0.0046) | <0.0001 |
| Bachelor's degree | 0.0332 | (0.0041) | <0.0001 | 0.0247 | (0.0040) | <0.0001 | 0.0306 | (0.0041) | <0.0001 | 0.0244 | (0.0040) | <0.0001 |
| Master's or Doctorate degree | 0.0488 | (0.0049) | <0.0001 | 0.0368 | (0.0045) | <0.0001 | 0.0423 | (0.0048) | <0.0001 | 0.0363 | (0.0045) | <0.0001 |
| *Income (% poverty level, ref: Poor <100%)* | | | | | | | | | | | | |
| Near poor (100%-124%) | 0.0171 | (0.0067) | 0.0118 | 0.0171 | (0.0061) | 0.0057 | 0.0180 | (0.0066) | 0.0069 | 0.0163 | (0.0060) | 0.0069 |
| Low income (125%-199%) | 0.0357 | (0.0054) | <0.0001 | 0.0271 | (0.0050) | <0.0001 | 0.0337 | (0.0055) | <0.0001 | 0.0270 | (0.0050) | <0.0001 |
| Middle income (200%-399%) | 0.0574 | (0.0049) | <0.0001 | 0.0494 | (0.0045) | <0.0001 | 0.0558 | (0.0049) | <0.0001 | 0.0488 | (0.0045) | <0.0001 |
| High income (≥400%) | 0.0765 | (0.0052) | <0.0001 | 0.0640 | (0.0047) | <0.0001 | 0.0729 | (0.0051) | <0.0001 | 0.0631 | (0.0047) | <0.0001 |
| *Cardiometabolic Conditions* | | | | | | | | | | | | |
| Diabetes |  |  |  | -0.0405 | (0.0038) | <0.0001 |  |  |  | -0.0368 | (0.0039) | <0.0001 |
| Heart Disease |  |  |  | -0.0469 | (0.0038) | <0.0001 |  |  |  | -0.0460 | (0.0038) | <0.0001 |
| High Blood Pressure |  |  |  | -0.0356 | (0.0031) | <0.0001 |  |  |  | -0.0342 | (0.0031) | <0.0001 |
| High Cholesterol |  |  |  | -0.0213 | (0.0028) | <0.0001 |  |  |  | -0.0215 | (0.0028) | <0.0001 |
| Stroke |  |  |  | -0.0741 | (0.0067) | <0.0001 |  |  |  | -0.0748 | (0.0066) | <0.0001 |
| Obesity |  |  |  | -0.0335 | (0.0024) | <0.0001 |  |  |  | -0.0177 | (0.0040) | <0.0001 |
| *Body Mass Index* | | | | | | | | | | | | |
| BMI (linear, centered at 25) |  |  |  |  |  |  | -0.0023 | (0.0003) | <0.0001 | -0.0000 | (0.0004) | 0.9280 |
| BMI² (quadratic) |  |  |  |  |  |  | -0.0001 | (0.0000) | <0.0001 | -0.0001 | (0.0000) | <0.0001 |

## Table S5. Survey-Weighted Linear Regression of EQ-5D Utility on Cardiometabolic Conditions and Covariates, MEPS 2018

|  | Model 1: Sociodemographic | | | Model 2: Sociodemographic + CMD | | | Model 3: Sociodemographic + BMI (centered at 25) | | | Model 4: Sociodemographic + CMD + BMI (centered at 25) | | |
| --- | --- | --- | --- | --- | --- | --- | --- | --- | --- | --- | --- | --- |
| Variable | Beta | (SE) | p | Beta | (SE) | p | Beta | (SE) | p | Beta | (SE) | p |
| Intercept | 0.8518 | (0.0047) | <0.0001 | 0.8703 | (0.0043) | <0.0001 | 0.8597 | (0.0047) | <0.0001 | 0.8729 | (0.0043) | <0.0001 |
| *Age group (ref: 18-29 years)* | | | | | | | | | | | | |
| 30-39 | -0.0258 | (0.0027) | <0.0001 | -0.0151 | (0.0024) | <0.0001 | -0.0206 | (0.0025) | <0.0001 | -0.0145 | (0.0023) | <0.0001 |
| 40-49 | -0.0480 | (0.0029) | <0.0001 | -0.0252 | (0.0027) | <0.0001 | -0.0397 | (0.0028) | <0.0001 | -0.0245 | (0.0026) | <0.0001 |
| 50-59 | -0.0758 | (0.0032) | <0.0001 | -0.0385 | (0.0031) | <0.0001 | -0.0701 | (0.0034) | <0.0001 | -0.0393 | (0.0029) | <0.0001 |
| 60-69 | -0.0970 | (0.0036) | <0.0001 | -0.0447 | (0.0035) | <0.0001 | -0.0924 | (0.0035) | <0.0001 | -0.0463 | (0.0034) | <0.0001 |
| 70-79 | -0.1195 | (0.0043) | <0.0001 | -0.0506 | (0.0039) | <0.0001 | -0.1163 | (0.0042) | <0.0001 | -0.0535 | (0.0038) | <0.0001 |
| 80+ | -0.1749 | (0.0053) | <0.0001 | -0.0997 | (0.0057) | <0.0001 | -0.1771 | (0.0055) | <0.0001 | -0.1031 | (0.0056) | <0.0001 |
| *Sex (ref: Female)* | | | | | | | | | | | | |
| Male | 0.0110 | (0.0016) | <0.0001 | 0.0154 | (0.0016) | <0.0001 | 0.0082 | (0.0016) | <0.0001 | 0.0137 | (0.0016) | <0.0001 |
| *Race/Ethnicity (ref: Non-Hispanic White)* | | | | | | | | | | | | |
| Non-Hispanic Black | 0.0069 | (0.0030) | 0.0237 | 0.0126 | (0.0028) | <0.0001 | 0.0131 | (0.0030) | <0.0001 | 0.0149 | (0.0028) | <0.0001 |
| Hispanic | 0.0295 | (0.0027) | <0.0001 | 0.0240 | (0.0024) | <0.0001 | 0.0277 | (0.0026) | <0.0001 | 0.0239 | (0.0024) | <0.0001 |
| Non-Hispanic Asian/Pacific Islander | 0.0268 | (0.0036) | <0.0001 | 0.0159 | (0.0035) | <0.0001 | 0.0158 | (0.0035) | <0.0001 | 0.0145 | (0.0036) | <0.001 |
| Non-Hispanic Other | -0.0215 | (0.0053) | <0.0001 | -0.0149 | (0.0050) | 0.0035 | -0.0181 | (0.0052) | <0.001 | -0.0144 | (0.0050) | 0.0047 |
| *Education (ref: No degree)* | | | | | | | | | | | | |
| High school diploma / GED | 0.0096 | (0.0028) | <0.001 | 0.0102 | (0.0027) | <0.001 | 0.0133 | (0.0030) | <0.0001 | 0.0108 | (0.0027) | <0.001 |
| Other post-secondary degree | 0.0164 | (0.0041) | <0.001 | 0.0191 | (0.0041) | <0.0001 | 0.0211 | (0.0045) | <0.0001 | 0.0195 | (0.0042) | <0.0001 |
| Bachelor's degree | 0.0386 | (0.0033) | <0.0001 | 0.0308 | (0.0033) | <0.0001 | 0.0382 | (0.0035) | <0.0001 | 0.0309 | (0.0033) | <0.0001 |
| Master's or Doctorate degree | 0.0495 | (0.0037) | <0.0001 | 0.0408 | (0.0037) | <0.0001 | 0.0487 | (0.0039) | <0.0001 | 0.0406 | (0.0036) | <0.0001 |
| *Income (% poverty level, ref: Poor <100%)* | | | | | | | | | | | | |
| Near poor (100%-124%) | 0.0300 | (0.0056) | <0.0001 | 0.0298 | (0.0053) | <0.0001 | 0.0292 | (0.0061) | <0.0001 | 0.0292 | (0.0054) | <0.0001 |
| Low income (125%-199%) | 0.0406 | (0.0050) | <0.0001 | 0.0378 | (0.0046) | <0.0001 | 0.0411 | (0.0048) | <0.0001 | 0.0374 | (0.0044) | <0.0001 |
| Middle income (200%-399%) | 0.0661 | (0.0040) | <0.0001 | 0.0601 | (0.0039) | <0.0001 | 0.0670 | (0.0042) | <0.0001 | 0.0594 | (0.0038) | <0.0001 |
| High income (≥400%) | 0.0867 | (0.0042) | <0.0001 | 0.0766 | (0.0039) | <0.0001 | 0.0851 | (0.0043) | <0.0001 | 0.0749 | (0.0039) | <0.0001 |
| *Cardiometabolic Conditions* | | | | | | | | | | | | |
| Diabetes |  |  |  | -0.0463 | (0.0042) | <0.0001 |  |  |  | -0.0434 | (0.0043) | <0.0001 |
| Heart Disease |  |  |  | -0.0459 | (0.0033) | <0.0001 |  |  |  | -0.0456 | (0.0032) | <0.0001 |
| High Blood Pressure |  |  |  | -0.0313 | (0.0027) | <0.0001 |  |  |  | -0.0286 | (0.0027) | <0.0001 |
| High Cholesterol |  |  |  | -0.0223 | (0.0020) | <0.0001 |  |  |  | -0.0225 | (0.0020) | <0.0001 |
| Stroke |  |  |  | -0.0782 | (0.0070) | <0.0001 |  |  |  | -0.0786 | (0.0069) | <0.0001 |
| Obesity |  |  |  | -0.0310 | (0.0018) | <0.0001 |  |  |  | -0.0134 | (0.0032) | <0.0001 |
| *Body Mass Index* | | | | | | | | | | | | |
| BMI (linear, centered at 25) |  |  |  |  |  |  | -0.0019 | (0.0001) | <0.0001 | -0.0006 | (0.0002) | 0.0055 |
| BMI² (quadratic) |  |  |  |  |  |  | -0.0001 | (0.0000) | <0.0001 | -0.0001 | (0.0000) | <0.0001 |

## Table S6. Survey-Weighted Linear Regression of EQ-5D Utility on Cardiometabolic Conditions and Covariates, MEPS 2016

|  | Model 1: Sociodemographic | | | Model 2: Sociodemographic + CMD | | | Model 3: Sociodemographic + BMI (centered at 25) | | | Model 4: Sociodemographic + CMD + BMI (centered at 25) | | |
| --- | --- | --- | --- | --- | --- | --- | --- | --- | --- | --- | --- | --- |
| Variable | Beta | (SE) | p | Beta | (SE) | p | Beta | (SE) | p | Beta | (SE) | p |
| Intercept | 0.8526 | (0.0044) | <0.0001 | 0.8709 | (0.0040) | <0.0001 | 0.8580 | (0.0045) | <0.0001 | 0.8718 | (0.0041) | <0.0001 |
| *Age group (ref: 18-29 years)* | | | | | | | | | | | | |
| 30-39 | -0.0304 | (0.0028) | <0.0001 | -0.0173 | (0.0025) | <0.0001 | -0.0235 | (0.0026) | <0.0001 | -0.0168 | (0.0026) | <0.0001 |
| 40-49 | -0.0539 | (0.0031) | <0.0001 | -0.0270 | (0.0029) | <0.0001 | -0.0445 | (0.0030) | <0.0001 | -0.0268 | (0.0029) | <0.0001 |
| 50-59 | -0.0790 | (0.0032) | <0.0001 | -0.0385 | (0.0028) | <0.0001 | -0.0702 | (0.0033) | <0.0001 | -0.0390 | (0.0029) | <0.0001 |
| 60-69 | -0.1089 | (0.0034) | <0.0001 | -0.0492 | (0.0031) | <0.0001 | -0.1006 | (0.0034) | <0.0001 | -0.0504 | (0.0032) | <0.0001 |
| 70-79 | -0.1287 | (0.0046) | <0.0001 | -0.0525 | (0.0044) | <0.0001 | -0.1233 | (0.0045) | <0.0001 | -0.0547 | (0.0044) | <0.0001 |
| 80+ | -0.1720 | (0.0059) | <0.0001 | -0.0884 | (0.0059) | <0.0001 | -0.1685 | (0.0060) | <0.0001 | -0.0912 | (0.0059) | <0.0001 |
| *Sex (ref: Female)* | | | | | | | | | | | | |
| Male | 0.0103 | (0.0016) | <0.0001 | 0.0155 | (0.0015) | <0.0001 | 0.0109 | (0.0015) | <0.0001 | 0.0148 | (0.0015) | <0.0001 |
| *Race/Ethnicity (ref: Non-Hispanic White)* | | | | | | | | | | | | |
| Non-Hispanic Black | 0.0067 | (0.0031) | 0.0327 | 0.0130 | (0.0027) | <0.0001 | 0.0132 | (0.0029) | <0.0001 | 0.0143 | (0.0026) | <0.0001 |
| Hispanic | 0.0244 | (0.0028) | <0.0001 | 0.0214 | (0.0024) | <0.0001 | 0.0259 | (0.0027) | <0.0001 | 0.0212 | (0.0024) | <0.0001 |
| Non-Hispanic Asian/Pacific Islander | 0.0158 | (0.0033) | <0.0001 | 0.0050 | (0.0030) | 0.0978 | 0.0053 | (0.0034) | 0.1230 | 0.0045 | (0.0031) | 0.1465 |
| Non-Hispanic Other | -0.0214 | (0.0066) | 0.0013 | -0.0153 | (0.0061) | 0.0132 | -0.0192 | (0.0065) | 0.0035 | -0.0157 | (0.0062) | 0.0119 |
| *Education (ref: No degree)* | | | | | | | | | | | | |
| High school diploma / GED | 0.0140 | (0.0031) | <0.0001 | 0.0149 | (0.0027) | <0.0001 | 0.0179 | (0.0030) | <0.0001 | 0.0157 | (0.0027) | <0.0001 |
| Other post-secondary degree | 0.0227 | (0.0042) | <0.0001 | 0.0208 | (0.0039) | <0.0001 | 0.0247 | (0.0042) | <0.0001 | 0.0214 | (0.0039) | <0.0001 |
| Bachelor's degree | 0.0403 | (0.0035) | <0.0001 | 0.0311 | (0.0031) | <0.0001 | 0.0393 | (0.0034) | <0.0001 | 0.0315 | (0.0031) | <0.0001 |
| Master's or Doctorate degree | 0.0503 | (0.0039) | <0.0001 | 0.0423 | (0.0036) | <0.0001 | 0.0493 | (0.0039) | <0.0001 | 0.0423 | (0.0036) | <0.0001 |
| *Income (% poverty level, ref: Poor <100%)* | | | | | | | | | | | | |
| Near poor (100%-124%) | 0.0253 | (0.0063) | <0.0001 | 0.0245 | (0.0054) | <0.0001 | 0.0259 | (0.0058) | <0.0001 | 0.0247 | (0.0053) | <0.0001 |
| Low income (125%-199%) | 0.0406 | (0.0041) | <0.0001 | 0.0372 | (0.0041) | <0.0001 | 0.0416 | (0.0041) | <0.0001 | 0.0372 | (0.0041) | <0.0001 |
| Middle income (200%-399%) | 0.0665 | (0.0034) | <0.0001 | 0.0595 | (0.0032) | <0.0001 | 0.0658 | (0.0035) | <0.0001 | 0.0589 | (0.0032) | <0.0001 |
| High income (≥400%) | 0.0859 | (0.0036) | <0.0001 | 0.0744 | (0.0033) | <0.0001 | 0.0832 | (0.0035) | <0.0001 | 0.0733 | (0.0033) | <0.0001 |
| *Cardiometabolic Conditions* | | | | | | | | | | | | |
| Diabetes |  |  |  | -0.0425 | (0.0036) | <0.0001 |  |  |  | -0.0401 | (0.0036) | <0.0001 |
| Heart Disease |  |  |  | -0.0546 | (0.0034) | <0.0001 |  |  |  | -0.0540 | (0.0033) | <0.0001 |
| High Blood Pressure |  |  |  | -0.0359 | (0.0025) | <0.0001 |  |  |  | -0.0340 | (0.0025) | <0.0001 |
| High Cholesterol |  |  |  | -0.0222 | (0.0024) | <0.0001 |  |  |  | -0.0224 | (0.0025) | <0.0001 |
| Stroke |  |  |  | -0.0751 | (0.0060) | <0.0001 |  |  |  | -0.0753 | (0.0061) | <0.0001 |
| Obesity |  |  |  | -0.0271 | (0.0021) | <0.0001 |  |  |  | -0.0109 | (0.0034) | 0.0015 |
| *Body Mass Index* | | | | | | | | | | | | |
| BMI (linear, centered at 25) |  |  |  |  |  |  | -0.0028 | (0.0003) | <0.0001 | -0.0007 | (0.0004) | 0.0764 |
| BMI² (quadratic) |  |  |  |  |  |  | -0.0001 | (0.0000) | <0.001 | -0.0001 | (0.0000) | <0.0001 |

## Table S7. Survey-Weighted Linear Regression of EQ-5D Utility on Cardiometabolic Conditions and Covariates, MEPS 2015

|  | Model 1: Sociodemographic | | | Model 2: Sociodemographic + CMD | | | Model 3: Sociodemographic + BMI (centered at 25) | | | Model 4: Sociodemographic + CMD + BMI (centered at 25) | | |
| --- | --- | --- | --- | --- | --- | --- | --- | --- | --- | --- | --- | --- |
| Variable | Beta | (SE) | p | Beta | (SE) | p | Beta | (SE) | p | Beta | (SE) | p |
| Intercept | 0.8534 | (0.0044) | <0.0001 | 0.8721 | (0.0043) | <0.0001 | 0.8561 | (0.0045) | <0.0001 | 0.8698 | (0.0043) | <0.0001 |
| *Age group (ref: 18-29 years)* | | | | | | | | | | | | |
| 30-39 | -0.0296 | (0.0030) | <0.0001 | -0.0174 | (0.0027) | <0.0001 | -0.0219 | (0.0029) | <0.0001 | -0.0161 | (0.0027) | <0.0001 |
| 40-49 | -0.0532 | (0.0029) | <0.0001 | -0.0264 | (0.0029) | <0.0001 | -0.0424 | (0.0028) | <0.0001 | -0.0250 | (0.0029) | <0.0001 |
| 50-59 | -0.0858 | (0.0029) | <0.0001 | -0.0446 | (0.0029) | <0.0001 | -0.0744 | (0.0028) | <0.0001 | -0.0433 | (0.0028) | <0.0001 |
| 60-69 | -0.1129 | (0.0045) | <0.0001 | -0.0517 | (0.0038) | <0.0001 | -0.1020 | (0.0042) | <0.0001 | -0.0513 | (0.0038) | <0.0001 |
| 70-79 | -0.1276 | (0.0043) | <0.0001 | -0.0508 | (0.0041) | <0.0001 | -0.1205 | (0.0041) | <0.0001 | -0.0511 | (0.0041) | <0.0001 |
| 80+ | -0.1673 | (0.0057) | <0.0001 | -0.0838 | (0.0061) | <0.0001 | -0.1626 | (0.0057) | <0.0001 | -0.0854 | (0.0062) | <0.0001 |
| *Sex (ref: Female)* | | | | | | | | | | | | |
| Male | 0.0120 | (0.0017) | <0.0001 | 0.0160 | (0.0016) | <0.0001 | 0.0135 | (0.0016) | <0.0001 | 0.0165 | (0.0015) | <0.0001 |
| *Race/Ethnicity (ref: Non-Hispanic White)* | | | | | | | | | | | | |
| Non-Hispanic Black | 0.0074 | (0.0030) | 0.0139 | 0.0139 | (0.0027) | <0.0001 | 0.0134 | (0.0029) | <0.0001 | 0.0148 | (0.0027) | <0.0001 |
| Hispanic | 0.0251 | (0.0027) | <0.0001 | 0.0219 | (0.0024) | <0.0001 | 0.0270 | (0.0026) | <0.0001 | 0.0223 | (0.0024) | <0.0001 |
| Non-Hispanic Asian/Pacific Islander | 0.0215 | (0.0034) | <0.0001 | 0.0121 | (0.0032) | <0.001 | 0.0098 | (0.0034) | 0.0049 | 0.0101 | (0.0032) | 0.0018 |
| Non-Hispanic Other | -0.0093 | (0.0066) | 0.1591 | -0.0037 | (0.0058) | 0.5203 | -0.0066 | (0.0061) | 0.2756 | -0.0036 | (0.0057) | 0.5321 |
| *Education (ref: No degree)* | | | | | | | | | | | | |
| High school diploma / GED | 0.0200 | (0.0031) | <0.0001 | 0.0190 | (0.0027) | <0.0001 | 0.0227 | (0.0029) | <0.0001 | 0.0195 | (0.0027) | <0.0001 |
| Other post-secondary degree |  |  |  |  |  |  |  |  |  |  |  |  |
| Bachelor's degree | 0.0467 | (0.0035) | <0.0001 | 0.0370 | (0.0032) | <0.0001 | 0.0454 | (0.0034) | <0.0001 | 0.0374 | (0.0032) | <0.0001 |
| Master's or Doctorate degree | 0.0580 | (0.0042) | <0.0001 | 0.0458 | (0.0038) | <0.0001 | 0.0539 | (0.0042) | <0.0001 | 0.0456 | (0.0038) | <0.0001 |
| *Income (% poverty level, ref: Poor <100%)* | | | | | | | | | | | | |
| Near poor (100%-124%) | 0.0022 | (0.0070) | 0.7579 | 0.0016 | (0.0071) | 0.8263 | 0.0040 | (0.0074) | 0.5900 | 0.0015 | (0.0070) | 0.8338 |
| Low income (125%-199%) | 0.0292 | (0.0048) | <0.0001 | 0.0292 | (0.0042) | <0.0001 | 0.0298 | (0.0047) | <0.0001 | 0.0291 | (0.0042) | <0.0001 |
| Middle income (200%-399%) | 0.0570 | (0.0040) | <0.0001 | 0.0516 | (0.0036) | <0.0001 | 0.0572 | (0.0040) | <0.0001 | 0.0516 | (0.0036) | <0.0001 |
| High income (≥400%) | 0.0789 | (0.0040) | <0.0001 | 0.0692 | (0.0035) | <0.0001 | 0.0770 | (0.0039) | <0.0001 | 0.0688 | (0.0035) | <0.0001 |
| *Cardiometabolic Conditions* | | | | | | | | | | | | |
| Diabetes |  |  |  | -0.0409 | (0.0036) | <0.0001 |  |  |  | -0.0393 | (0.0037) | <0.0001 |
| Heart Disease |  |  |  | -0.0507 | (0.0030) | <0.0001 |  |  |  | -0.0506 | (0.0030) | <0.0001 |
| High Blood Pressure |  |  |  | -0.0334 | (0.0024) | <0.0001 |  |  |  | -0.0317 | (0.0024) | <0.0001 |
| High Cholesterol |  |  |  | -0.0272 | (0.0024) | <0.0001 |  |  |  | -0.0267 | (0.0024) | <0.0001 |
| Stroke |  |  |  | -0.0724 | (0.0062) | <0.0001 |  |  |  | -0.0725 | (0.0063) | <0.0001 |
| Obesity |  |  |  | -0.0287 | (0.0020) | <0.0001 |  |  |  | -0.0118 | (0.0031) | <0.001 |
| *Body Mass Index* | | | | | | | | | | | | |
| BMI (linear, centered at 25) |  |  |  |  |  |  | -0.0039 | (0.0002) | <0.0001 | -0.0017 | (0.0003) | <0.0001 |
| BMI² (quadratic) |  |  |  |  |  |  | 0.0000 | (0.0000) | 0.0014 | 0.0000 | (0.0000) | 0.1598 |

## Table S8. Survey-Weighted Linear Regression of EQ-5D Utility on Cardiometabolic Conditions and Covariates, MEPS 2014

|  | Model 1: Sociodemographic | | | Model 2: Sociodemographic + CMD | | | Model 3: Sociodemographic + BMI (centered at 25) | | | Model 4: Sociodemographic + CMD + BMI (centered at 25) | | |
| --- | --- | --- | --- | --- | --- | --- | --- | --- | --- | --- | --- | --- |
| Variable | Beta | (SE) | p | Beta | (SE) | p | Beta | (SE) | p | Beta | (SE) | p |
| Intercept | 0.8531 | (0.0051) | <0.0001 | 0.8730 | (0.0047) | <0.0001 | 0.8555 | (0.0051) | <0.0001 | 0.8722 | (0.0048) | <0.0001 |
| *Age group (ref: 18-29 years)* | | | | | | | | | | | | |
| 30-39 | -0.0335 | (0.0025) | <0.0001 | -0.0209 | (0.0024) | <0.0001 | -0.0253 | (0.0025) | <0.0001 | -0.0204 | (0.0025) | <0.0001 |
| 40-49 | -0.0575 | (0.0031) | <0.0001 | -0.0316 | (0.0029) | <0.0001 | -0.0463 | (0.0031) | <0.0001 | -0.0310 | (0.0029) | <0.0001 |
| 50-59 | -0.0920 | (0.0034) | <0.0001 | -0.0513 | (0.0031) | <0.0001 | -0.0807 | (0.0034) | <0.0001 | -0.0510 | (0.0032) | <0.0001 |
| 60-69 | -0.1171 | (0.0041) | <0.0001 | -0.0559 | (0.0036) | <0.0001 | -0.1050 | (0.0039) | <0.0001 | -0.0558 | (0.0036) | <0.0001 |
| 70-79 | -0.1368 | (0.0056) | <0.0001 | -0.0616 | (0.0052) | <0.0001 | -0.1289 | (0.0054) | <0.0001 | -0.0621 | (0.0051) | <0.0001 |
| 80+ | -0.1697 | (0.0067) | <0.0001 | -0.0874 | (0.0069) | <0.0001 | -0.1668 | (0.0068) | <0.0001 | -0.0881 | (0.0069) | <0.0001 |
| *Sex (ref: Female)* | | | | | | | | | | | | |
| Male | 0.0127 | (0.0019) | <0.0001 | 0.0164 | (0.0017) | <0.0001 | 0.0138 | (0.0019) | <0.0001 | 0.0163 | (0.0017) | <0.0001 |
| *Race/Ethnicity (ref: Non-Hispanic White)* | | | | | | | | | | | | |
| Non-Hispanic Black | 0.0042 | (0.0028) | 0.1418 | 0.0106 | (0.0027) | <0.001 | 0.0097 | (0.0029) | <0.001 | 0.0111 | (0.0027) | <0.0001 |
| Hispanic | 0.0266 | (0.0031) | <0.0001 | 0.0229 | (0.0027) | <0.0001 | 0.0280 | (0.0030) | <0.0001 | 0.0231 | (0.0027) | <0.0001 |
| Non-Hispanic Asian/Pacific Islander | 0.0213 | (0.0036) | <0.0001 | 0.0106 | (0.0034) | 0.0021 | 0.0096 | (0.0037) | 0.0104 | 0.0100 | (0.0034) | 0.0040 |
| Non-Hispanic Other | -0.0119 | (0.0060) | 0.0485 | -0.0064 | (0.0060) | 0.2838 | -0.0103 | (0.0060) | 0.0846 | -0.0062 | (0.0059) | 0.2943 |
| *Education (ref: No degree)* | | | | | | | | | | | | |
| High school diploma / GED | 0.0179 | (0.0034) | <0.0001 | 0.0175 | (0.0032) | <0.0001 | 0.0207 | (0.0034) | <0.0001 | 0.0178 | (0.0032) | <0.0001 |
| Other post-secondary degree |  |  |  |  |  |  |  |  |  |  |  |  |
| Bachelor's degree | 0.0437 | (0.0042) | <0.0001 | 0.0358 | (0.0037) | <0.0001 | 0.0421 | (0.0041) | <0.0001 | 0.0360 | (0.0037) | <0.0001 |
| Master's or Doctorate degree | 0.0546 | (0.0040) | <0.0001 | 0.0433 | (0.0037) | <0.0001 | 0.0517 | (0.0039) | <0.0001 | 0.0434 | (0.0037) | <0.0001 |
| *Income (% poverty level, ref: Poor <100%)* | | | | | | | | | | | | |
| Near poor (100%-124%) | 0.0151 | (0.0073) | 0.0410 | 0.0133 | (0.0064) | 0.0390 | 0.0149 | (0.0072) | 0.0399 | 0.0131 | (0.0064) | 0.0421 |
| Low income (125%-199%) | 0.0414 | (0.0050) | <0.0001 | 0.0346 | (0.0046) | <0.0001 | 0.0409 | (0.0048) | <0.0001 | 0.0345 | (0.0046) | <0.0001 |
| Middle income (200%-399%) | 0.0657 | (0.0040) | <0.0001 | 0.0573 | (0.0036) | <0.0001 | 0.0644 | (0.0040) | <0.0001 | 0.0572 | (0.0036) | <0.0001 |
| High income (≥400%) | 0.0883 | (0.0042) | <0.0001 | 0.0755 | (0.0037) | <0.0001 | 0.0859 | (0.0042) | <0.0001 | 0.0752 | (0.0037) | <0.0001 |
| *Cardiometabolic Conditions* | | | | | | | | | | | | |
| Diabetes |  |  |  | -0.0498 | (0.0040) | <0.0001 |  |  |  | -0.0488 | (0.0041) | <0.0001 |
| Heart Disease |  |  |  | -0.0506 | (0.0037) | <0.0001 |  |  |  | -0.0505 | (0.0037) | <0.0001 |
| High Blood Pressure |  |  |  | -0.0326 | (0.0025) | <0.0001 |  |  |  | -0.0317 | (0.0026) | <0.0001 |
| High Cholesterol |  |  |  | -0.0210 | (0.0025) | <0.0001 |  |  |  | -0.0208 | (0.0025) | <0.0001 |
| Stroke |  |  |  | -0.0640 | (0.0066) | <0.0001 |  |  |  | -0.0640 | (0.0066) | <0.0001 |
| Obesity |  |  |  | -0.0298 | (0.0021) | <0.0001 |  |  |  | -0.0207 | (0.0035) | <0.0001 |
| *Body Mass Index* | | | | | | | | | | | | |
| BMI (linear, centered at 25) |  |  |  |  |  |  | -0.0036 | (0.0003) | <0.0001 | -0.0007 | (0.0004) | 0.0710 |
| BMI² (quadratic) |  |  |  |  |  |  | 0.0000 | (0.0000) | 0.5297 | -0.0000 | (0.0000) | 0.3966 |

## Table S9. Survey-Weighted Linear Regression of EQ-5D Utility on Cardiometabolic Conditions and Covariates, MEPS 2013

|  | Model 1: Sociodemographic | | | Model 2: Sociodemographic + CMD | | | Model 3: Sociodemographic + BMI (centered at 25) | | | Model 4: Sociodemographic + CMD + BMI (centered at 25) | | |
| --- | --- | --- | --- | --- | --- | --- | --- | --- | --- | --- | --- | --- |
| Variable | Beta | (SE) | p | Beta | (SE) | p | Beta | (SE) | p | Beta | (SE) | p |
| Intercept | 0.8539 | (0.0049) | <0.0001 | 0.8719 | (0.0044) | <0.0001 | 0.8594 | (0.0048) | <0.0001 | 0.8737 | (0.0046) | <0.0001 |
| *Age group (ref: 18-29 years)* | | | | | | | | | | | | |
| 30-39 | -0.0372 | (0.0025) | <0.0001 | -0.0246 | (0.0024) | <0.0001 | -0.0303 | (0.0025) | <0.0001 | -0.0245 | (0.0024) | <0.0001 |
| 40-49 | -0.0621 | (0.0030) | <0.0001 | -0.0348 | (0.0028) | <0.0001 | -0.0511 | (0.0030) | <0.0001 | -0.0346 | (0.0028) | <0.0001 |
| 50-59 | -0.0906 | (0.0032) | <0.0001 | -0.0500 | (0.0031) | <0.0001 | -0.0807 | (0.0032) | <0.0001 | -0.0508 | (0.0031) | <0.0001 |
| 60-69 | -0.1181 | (0.0037) | <0.0001 | -0.0553 | (0.0038) | <0.0001 | -0.1069 | (0.0034) | <0.0001 | -0.0566 | (0.0037) | <0.0001 |
| 70-79 | -0.1399 | (0.0044) | <0.0001 | -0.0620 | (0.0045) | <0.0001 | -0.1325 | (0.0045) | <0.0001 | -0.0645 | (0.0046) | <0.0001 |
| 80+ | -0.1804 | (0.0067) | <0.0001 | -0.0985 | (0.0070) | <0.0001 | -0.1774 | (0.0064) | <0.0001 | -0.1013 | (0.0069) | <0.0001 |
| *Sex (ref: Female)* | | | | | | | | | | | | |
| Male | 0.0117 | (0.0019) | <0.0001 | 0.0163 | (0.0018) | <0.0001 | 0.0117 | (0.0019) | <0.0001 | 0.0150 | (0.0018) | <0.0001 |
| *Race/Ethnicity (ref: Non-Hispanic White)* | | | | | | | | | | | | |
| Non-Hispanic Black | 0.0030 | (0.0026) | 0.2554 | 0.0102 | (0.0024) | <0.0001 | 0.0078 | (0.0026) | 0.0031 | 0.0100 | (0.0024) | <0.0001 |
| Hispanic | 0.0232 | (0.0026) | <0.0001 | 0.0191 | (0.0022) | <0.0001 | 0.0234 | (0.0025) | <0.0001 | 0.0184 | (0.0022) | <0.0001 |
| Non-Hispanic Asian/Pacific Islander | 0.0153 | (0.0041) | <0.001 | 0.0038 | (0.0036) | 0.2849 | 0.0034 | (0.0041) | 0.4007 | 0.0030 | (0.0035) | 0.3908 |
| Non-Hispanic Other | -0.0160 | (0.0059) | 0.0074 | -0.0070 | (0.0055) | 0.2039 | -0.0140 | (0.0061) | 0.0238 | -0.0071 | (0.0056) | 0.2042 |
| *Education (ref: No degree)* | | | | | | | | | | | | |
| High school diploma / GED | 0.0149 | (0.0033) | <0.0001 | 0.0148 | (0.0030) | <0.0001 | 0.0172 | (0.0033) | <0.0001 | 0.0150 | (0.0030) | <0.0001 |
| Other post-secondary degree |  |  |  |  |  |  |  |  |  |  |  |  |
| Bachelor's degree | 0.0396 | (0.0038) | <0.0001 | 0.0327 | (0.0036) | <0.0001 | 0.0378 | (0.0038) | <0.0001 | 0.0324 | (0.0036) | <0.0001 |
| Master's or Doctorate degree | 0.0535 | (0.0045) | <0.0001 | 0.0430 | (0.0040) | <0.0001 | 0.0503 | (0.0044) | <0.0001 | 0.0433 | (0.0039) | <0.0001 |
| *Income (% poverty level, ref: Poor <100%)* | | | | | | | | | | | | |
| Near poor (100%-124%) | 0.0253 | (0.0063) | <0.0001 | 0.0244 | (0.0059) | <0.0001 | 0.0251 | (0.0060) | <0.0001 | 0.0239 | (0.0058) | <0.0001 |
| Low income (125%-199%) | 0.0418 | (0.0047) | <0.0001 | 0.0385 | (0.0041) | <0.0001 | 0.0416 | (0.0045) | <0.0001 | 0.0384 | (0.0041) | <0.0001 |
| Middle income (200%-399%) | 0.0668 | (0.0040) | <0.0001 | 0.0606 | (0.0037) | <0.0001 | 0.0669 | (0.0038) | <0.0001 | 0.0604 | (0.0036) | <0.0001 |
| High income (≥400%) | 0.0911 | (0.0039) | <0.0001 | 0.0799 | (0.0036) | <0.0001 | 0.0875 | (0.0038) | <0.0001 | 0.0792 | (0.0035) | <0.0001 |
| *Cardiometabolic Conditions* | | | | | | | | | | | | |
| Diabetes |  |  |  | -0.0402 | (0.0039) | <0.0001 |  |  |  | -0.0378 | (0.0039) | <0.0001 |
| Heart Disease |  |  |  | -0.0482 | (0.0039) | <0.0001 |  |  |  | -0.0479 | (0.0039) | <0.0001 |
| High Blood Pressure |  |  |  | -0.0357 | (0.0025) | <0.0001 |  |  |  | -0.0341 | (0.0025) | <0.0001 |
| High Cholesterol |  |  |  | -0.0256 | (0.0025) | <0.0001 |  |  |  | -0.0258 | (0.0026) | <0.0001 |
| Stroke |  |  |  | -0.0590 | (0.0074) | <0.0001 |  |  |  | -0.0592 | (0.0074) | <0.0001 |
| Obesity |  |  |  | -0.0318 | (0.0022) | <0.0001 |  |  |  | -0.0192 | (0.0033) | <0.0001 |
| *Body Mass Index* | | | | | | | | | | | | |
| BMI (linear, centered at 25) |  |  |  |  |  |  | -0.0030 | (0.0003) | <0.0001 | -0.0002 | (0.0004) | 0.5272 |
| BMI² (quadratic) |  |  |  |  |  |  | -0.0001 | (0.0000) | 0.0029 | -0.0001 | (0.0000) | <0.0001 |

## Table S10. Survey-Weighted Linear Regression of EQ-5D Utility on Cardiometabolic Conditions and Covariates, MEPS 2012

|  | Model 1: Sociodemographic | | | Model 2: Sociodemographic + CMD | | | Model 3: Sociodemographic + BMI (centered at 25) | | | Model 4: Sociodemographic + CMD + BMI (centered at 25) | | |
| --- | --- | --- | --- | --- | --- | --- | --- | --- | --- | --- | --- | --- |
| Variable | Beta | (SE) | p | Beta | (SE) | p | Beta | (SE) | p | Beta | (SE) | p |
| Intercept | 0.8420 | (0.0062) | <0.0001 | 0.8580 | (0.0060) | <0.0001 | 0.8456 | (0.0067) | <0.0001 | 0.8573 | (0.0063) | <0.0001 |
| *Age group (ref: 18-29 years)* | | | | | | | | | | | | |
| 30-39 | -0.0403 | (0.0035) | <0.0001 | -0.0266 | (0.0035) | <0.0001 | -0.0321 | (0.0034) | <0.0001 | -0.0254 | (0.0035) | <0.0001 |
| 40-49 | -0.0607 | (0.0039) | <0.0001 | -0.0396 | (0.0037) | <0.0001 | -0.0524 | (0.0039) | <0.0001 | -0.0390 | (0.0038) | <0.0001 |
| 50-59 | -0.0903 | (0.0043) | <0.0001 | -0.0530 | (0.0045) | <0.0001 | -0.0799 | (0.0043) | <0.0001 | -0.0523 | (0.0045) | <0.0001 |
| 60-69 | -0.1086 | (0.0049) | <0.0001 | -0.0523 | (0.0052) | <0.0001 | -0.0991 | (0.0048) | <0.0001 | -0.0525 | (0.0051) | <0.0001 |
| 70-79 | -0.1355 | (0.0060) | <0.0001 | -0.0614 | (0.0064) | <0.0001 | -0.1261 | (0.0060) | <0.0001 | -0.0626 | (0.0063) | <0.0001 |
| 80+ | -0.1769 | (0.0080) | <0.0001 | -0.1033 | (0.0085) | <0.0001 | -0.1780 | (0.0079) | <0.0001 | -0.1054 | (0.0085) | <0.0001 |
| *Sex (ref: Female)* | | | | | | | | | | | | |
| Male | 0.0151 | (0.0024) | <0.0001 | 0.0195 | (0.0023) | <0.0001 | 0.0162 | (0.0024) | <0.0001 | 0.0197 | (0.0024) | <0.0001 |
| *Race/Ethnicity (ref: Non-Hispanic White)* | | | | | | | | | | | | |
| Non-Hispanic Black | 0.0085 | (0.0036) | 0.0192 | 0.0174 | (0.0035) | <0.0001 | 0.0148 | (0.0037) | <0.0001 | 0.0183 | (0.0035) | <0.0001 |
| Hispanic | 0.0236 | (0.0036) | <0.0001 | 0.0203 | (0.0033) | <0.0001 | 0.0245 | (0.0037) | <0.0001 | 0.0205 | (0.0034) | <0.0001 |
| Non-Hispanic Asian/Pacific Islander | 0.0191 | (0.0051) | <0.001 | 0.0094 | (0.0051) | 0.0665 | 0.0090 | (0.0053) | 0.0878 | 0.0082 | (0.0052) | 0.1160 |
| Non-Hispanic Other | -0.0223 | (0.0106) | 0.0368 | -0.0153 | (0.0100) | 0.1262 | -0.0194 | (0.0104) | 0.0639 | -0.0145 | (0.0097) | 0.1383 |
| *Education (ref: No degree)* | | | | | | | | | | | | |
| High school diploma / GED | 0.0145 | (0.0036) | <0.001 | 0.0165 | (0.0034) | <0.0001 | 0.0188 | (0.0036) | <0.0001 | 0.0171 | (0.0034) | <0.0001 |
| Other post-secondary degree | 0.0345 | (0.0060) | <0.0001 | 0.0323 | (0.0058) | <0.0001 | 0.0371 | (0.0059) | <0.0001 | 0.0326 | (0.0058) | <0.0001 |
| Bachelor's degree | 0.0450 | (0.0052) | <0.0001 | 0.0392 | (0.0048) | <0.0001 | 0.0454 | (0.0051) | <0.0001 | 0.0396 | (0.0048) | <0.0001 |
| Master's or Doctorate degree | 0.0514 | (0.0060) | <0.0001 | 0.0415 | (0.0058) | <0.0001 | 0.0495 | (0.0059) | <0.0001 | 0.0416 | (0.0058) | <0.0001 |
| *Income (% poverty level, ref: Poor <100%)* | | | | | | | | | | | | |
| Near poor (100%-124%) | 0.0276 | (0.0085) | 0.0014 | 0.0255 | (0.0074) | <0.001 | 0.0258 | (0.0082) | 0.0019 | 0.0249 | (0.0074) | <0.001 |
| Low income (125%-199%) | 0.0613 | (0.0061) | <0.0001 | 0.0555 | (0.0057) | <0.0001 | 0.0590 | (0.0061) | <0.0001 | 0.0549 | (0.0057) | <0.0001 |
| Middle income (200%-399%) | 0.0714 | (0.0054) | <0.0001 | 0.0638 | (0.0052) | <0.0001 | 0.0684 | (0.0055) | <0.0001 | 0.0630 | (0.0052) | <0.0001 |
| High income (≥400%) | 0.0934 | (0.0057) | <0.0001 | 0.0816 | (0.0054) | <0.0001 | 0.0886 | (0.0057) | <0.0001 | 0.0804 | (0.0054) | <0.0001 |
| *Cardiometabolic Conditions* | | | | | | | | | | | | |
| Diabetes |  |  |  | -0.0454 | (0.0051) | <0.0001 |  |  |  | -0.0435 | (0.0050) | <0.0001 |
| Heart Disease |  |  |  | -0.0398 | (0.0047) | <0.0001 |  |  |  | -0.0400 | (0.0047) | <0.0001 |
| High Blood Pressure |  |  |  | -0.0404 | (0.0034) | <0.0001 |  |  |  | -0.0389 | (0.0035) | <0.0001 |
| High Cholesterol |  |  |  | -0.0148 | (0.0033) | <0.0001 |  |  |  | -0.0144 | (0.0033) | <0.0001 |
| Stroke |  |  |  | -0.0769 | (0.0101) | <0.0001 |  |  |  | -0.0770 | (0.0100) | <0.0001 |
| Obesity |  |  |  | -0.0290 | (0.0027) | <0.0001 |  |  |  | -0.0115 | (0.0042) | 0.0075 |
| *Body Mass Index* | | | | | | | | | | | | |
| BMI (linear, centered at 25) |  |  |  |  |  |  | -0.0035 | (0.0005) | <0.0001 | -0.0013 | (0.0006) | 0.0229 |
| BMI² (quadratic) |  |  |  |  |  |  | -0.0000 | (0.0000) | 0.6780 | -0.0000 | (0.0000) | 0.2652 |

## Table S11. Survey-Weighted Linear Regression of EQ-5D Utility on Cardiometabolic Conditions and Covariates, MEPS 2011

|  | Model 1: Sociodemographic | | | Model 2: Sociodemographic + CMD | | | Model 3: Sociodemographic + BMI (centered at 25) | | | Model 4: Sociodemographic + CMD + BMI (centered at 25) | | |
| --- | --- | --- | --- | --- | --- | --- | --- | --- | --- | --- | --- | --- |
| Variable | Beta | (SE) | p | Beta | (SE) | p | Beta | (SE) | p | Beta | (SE) | p |
| Intercept | 0.8444 | (0.0040) | <0.0001 | 0.8614 | (0.0035) | <0.0001 | 0.8498 | (0.0038) | <0.0001 | 0.8635 | (0.0037) | <0.0001 |
| *Age group (ref: 18-29 years)* | | | | | | | | | | | | |
| 30-39 | -0.0389 | (0.0025) | <0.0001 | -0.0257 | (0.0025) | <0.0001 | -0.0311 | (0.0025) | <0.0001 | -0.0253 | (0.0025) | <0.0001 |
| 40-49 | -0.0639 | (0.0029) | <0.0001 | -0.0395 | (0.0028) | <0.0001 | -0.0546 | (0.0030) | <0.0001 | -0.0393 | (0.0028) | <0.0001 |
| 50-59 | -0.0920 | (0.0030) | <0.0001 | -0.0514 | (0.0033) | <0.0001 | -0.0815 | (0.0031) | <0.0001 | -0.0518 | (0.0032) | <0.0001 |
| 60-69 | -0.1111 | (0.0037) | <0.0001 | -0.0505 | (0.0038) | <0.0001 | -0.1003 | (0.0037) | <0.0001 | -0.0518 | (0.0037) | <0.0001 |
| 70-79 | -0.1346 | (0.0045) | <0.0001 | -0.0541 | (0.0049) | <0.0001 | -0.1247 | (0.0044) | <0.0001 | -0.0569 | (0.0049) | <0.0001 |
| 80+ | -0.1696 | (0.0059) | <0.0001 | -0.0885 | (0.0065) | <0.0001 | -0.1667 | (0.0062) | <0.0001 | -0.0908 | (0.0065) | <0.0001 |
| *Sex (ref: Female)* | | | | | | | | | | | | |
| Male | 0.0144 | (0.0016) | <0.0001 | 0.0204 | (0.0016) | <0.0001 | 0.0153 | (0.0016) | <0.0001 | 0.0192 | (0.0016) | <0.0001 |
| *Race/Ethnicity (ref: Non-Hispanic White)* | | | | | | | | | | | | |
| Non-Hispanic Black | 0.0022 | (0.0028) | 0.4321 | 0.0109 | (0.0025) | <0.0001 | 0.0090 | (0.0028) | 0.0014 | 0.0112 | (0.0025) | <0.0001 |
| Hispanic | 0.0219 | (0.0024) | <0.0001 | 0.0194 | (0.0023) | <0.0001 | 0.0234 | (0.0024) | <0.0001 | 0.0188 | (0.0023) | <0.0001 |
| Non-Hispanic Asian/Pacific Islander | 0.0228 | (0.0033) | <0.0001 | 0.0131 | (0.0030) | <0.0001 | 0.0137 | (0.0033) | <0.0001 | 0.0126 | (0.0031) | <0.0001 |
| Non-Hispanic Other | -0.0279 | (0.0078) | <0.001 | -0.0186 | (0.0074) | 0.0123 | -0.0212 | (0.0076) | 0.0057 | -0.0176 | (0.0074) | 0.0179 |
| *Education (ref: No degree)* | | | | | | | | | | | | |
| High school diploma / GED | 0.0203 | (0.0028) | <0.0001 | 0.0195 | (0.0026) | <0.0001 | 0.0229 | (0.0027) | <0.0001 | 0.0200 | (0.0026) | <0.0001 |
| Other post-secondary degree | 0.0331 | (0.0046) | <0.0001 | 0.0330 | (0.0043) | <0.0001 | 0.0363 | (0.0045) | <0.0001 | 0.0339 | (0.0043) | <0.0001 |
| Bachelor's degree | 0.0494 | (0.0036) | <0.0001 | 0.0432 | (0.0033) | <0.0001 | 0.0487 | (0.0036) | <0.0001 | 0.0437 | (0.0033) | <0.0001 |
| Master's or Doctorate degree | 0.0601 | (0.0036) | <0.0001 | 0.0500 | (0.0034) | <0.0001 | 0.0584 | (0.0036) | <0.0001 | 0.0503 | (0.0035) | <0.0001 |
| *Income (% poverty level, ref: Poor <100%)* | | | | | | | | | | | | |
| Near poor (100%-124%) | 0.0222 | (0.0058) | <0.001 | 0.0193 | (0.0053) | <0.001 | 0.0207 | (0.0055) | <0.001 | 0.0184 | (0.0052) | <0.001 |
| Low income (125%-199%) | 0.0411 | (0.0042) | <0.0001 | 0.0351 | (0.0036) | <0.0001 | 0.0398 | (0.0039) | <0.0001 | 0.0341 | (0.0036) | <0.0001 |
| Middle income (200%-399%) | 0.0612 | (0.0039) | <0.0001 | 0.0542 | (0.0035) | <0.0001 | 0.0587 | (0.0038) | <0.0001 | 0.0529 | (0.0035) | <0.0001 |
| High income (≥400%) | 0.0853 | (0.0038) | <0.0001 | 0.0736 | (0.0034) | <0.0001 | 0.0809 | (0.0037) | <0.0001 | 0.0723 | (0.0034) | <0.0001 |
| *Cardiometabolic Conditions* | | | | | | | | | | | | |
| Diabetes |  |  |  | -0.0427 | (0.0036) | <0.0001 |  |  |  | -0.0391 | (0.0035) | <0.0001 |
| Heart Disease |  |  |  | -0.0533 | (0.0036) | <0.0001 |  |  |  | -0.0528 | (0.0036) | <0.0001 |
| High Blood Pressure |  |  |  | -0.0387 | (0.0025) | <0.0001 |  |  |  | -0.0367 | (0.0024) | <0.0001 |
| High Cholesterol |  |  |  | -0.0190 | (0.0029) | <0.0001 |  |  |  | -0.0197 | (0.0028) | <0.0001 |
| Stroke |  |  |  | -0.0734 | (0.0065) | <0.0001 |  |  |  | -0.0738 | (0.0065) | <0.0001 |
| Obesity |  |  |  | -0.0292 | (0.0021) | <0.0001 |  |  |  | -0.0135 | (0.0032) | <0.0001 |
| *Body Mass Index* | | | | | | | | | | | | |
| BMI (linear, centered at 25) |  |  |  |  |  |  | -0.0027 | (0.0003) | <0.0001 | -0.0003 | (0.0003) | 0.4069 |
| BMI² (quadratic) |  |  |  |  |  |  | -0.0001 | (0.0000) | <0.0001 | -0.0001 | (0.0000) | <0.0001 |

## Table S12. Survey-Weighted Linear Regression of EQ-5D Utility on Cardiometabolic Conditions and Covariates, MEPS 2010

|  | Model 1: Sociodemographic | | | Model 2: Sociodemographic + CMD | | | Model 3: Sociodemographic + BMI (centered at 25) | | | Model 4: Sociodemographic + CMD + BMI (centered at 25) | | |
| --- | --- | --- | --- | --- | --- | --- | --- | --- | --- | --- | --- | --- |
| Variable | Beta | (SE) | p | Beta | (SE) | p | Beta | (SE) | p | Beta | (SE) | p |
| Intercept | 0.8451 | (0.0042) | <0.0001 | 0.8627 | (0.0039) | <0.0001 | 0.8520 | (0.0042) | <0.0001 | 0.8646 | (0.0041) | <0.0001 |
| *Age group (ref: 18-29 years)* | | | | | | | | | | | | |
| 30-39 | -0.0347 | (0.0024) | <0.0001 | -0.0216 | (0.0023) | <0.0001 | -0.0258 | (0.0025) | <0.0001 | -0.0206 | (0.0024) | <0.0001 |
| 40-49 | -0.0621 | (0.0029) | <0.0001 | -0.0381 | (0.0026) | <0.0001 | -0.0519 | (0.0029) | <0.0001 | -0.0377 | (0.0026) | <0.0001 |
| 50-59 | -0.0937 | (0.0029) | <0.0001 | -0.0524 | (0.0027) | <0.0001 | -0.0832 | (0.0028) | <0.0001 | -0.0527 | (0.0027) | <0.0001 |
| 60-69 | -0.1106 | (0.0033) | <0.0001 | -0.0509 | (0.0032) | <0.0001 | -0.1000 | (0.0033) | <0.0001 | -0.0520 | (0.0033) | <0.0001 |
| 70-79 | -0.1329 | (0.0045) | <0.0001 | -0.0541 | (0.0041) | <0.0001 | -0.1254 | (0.0044) | <0.0001 | -0.0568 | (0.0042) | <0.0001 |
| 80+ | -0.1768 | (0.0065) | <0.0001 | -0.0956 | (0.0066) | <0.0001 | -0.1760 | (0.0064) | <0.0001 | -0.0985 | (0.0065) | <0.0001 |
| *Sex (ref: Female)* | | | | | | | | | | | | |
| Male | 0.0137 | (0.0017) | <0.0001 | 0.0172 | (0.0017) | <0.0001 | 0.0138 | (0.0017) | <0.0001 | 0.0162 | (0.0017) | <0.0001 |
| *Race/Ethnicity (ref: Non-Hispanic White)* | | | | | | | | | | | | |
| Non-Hispanic Black | 0.0025 | (0.0028) | 0.3696 | 0.0114 | (0.0025) | <0.0001 | 0.0081 | (0.0027) | 0.0031 | 0.0115 | (0.0025) | <0.0001 |
| Hispanic | 0.0234 | (0.0030) | <0.0001 | 0.0196 | (0.0027) | <0.0001 | 0.0239 | (0.0028) | <0.0001 | 0.0188 | (0.0027) | <0.0001 |
| Non-Hispanic Asian/Pacific Islander | 0.0167 | (0.0038) | <0.0001 | 0.0063 | (0.0035) | 0.0734 | 0.0059 | (0.0038) | 0.1221 | 0.0060 | (0.0035) | 0.0905 |
| Non-Hispanic Other | -0.0158 | (0.0074) | 0.0341 | -0.0074 | (0.0073) | 0.3112 | -0.0103 | (0.0075) | 0.1705 | -0.0065 | (0.0073) | 0.3792 |
| *Education (ref: No degree)* | | | | | | | | | | | | |
| High school diploma / GED | 0.0224 | (0.0030) | <0.0001 | 0.0201 | (0.0029) | <0.0001 | 0.0228 | (0.0030) | <0.0001 | 0.0202 | (0.0029) | <0.0001 |
| Other post-secondary degree | 0.0352 | (0.0042) | <0.0001 | 0.0330 | (0.0042) | <0.0001 | 0.0352 | (0.0042) | <0.0001 | 0.0329 | (0.0042) | <0.0001 |
| Bachelor's degree | 0.0514 | (0.0034) | <0.0001 | 0.0432 | (0.0032) | <0.0001 | 0.0483 | (0.0033) | <0.0001 | 0.0428 | (0.0033) | <0.0001 |
| Master's or Doctorate degree | 0.0594 | (0.0039) | <0.0001 | 0.0481 | (0.0037) | <0.0001 | 0.0553 | (0.0039) | <0.0001 | 0.0476 | (0.0038) | <0.0001 |
| *Income (% poverty level, ref: Poor <100%)* | | | | | | | | | | | | |
| Near poor (100%-124%) | 0.0212 | (0.0055) | <0.001 | 0.0241 | (0.0050) | <0.0001 | 0.0232 | (0.0053) | <0.0001 | 0.0240 | (0.0049) | <0.0001 |
| Low income (125%-199%) | 0.0391 | (0.0047) | <0.0001 | 0.0361 | (0.0043) | <0.0001 | 0.0391 | (0.0046) | <0.0001 | 0.0356 | (0.0043) | <0.0001 |
| Middle income (200%-399%) | 0.0596 | (0.0037) | <0.0001 | 0.0552 | (0.0035) | <0.0001 | 0.0582 | (0.0036) | <0.0001 | 0.0545 | (0.0035) | <0.0001 |
| High income (≥400%) | 0.0852 | (0.0039) | <0.0001 | 0.0767 | (0.0034) | <0.0001 | 0.0827 | (0.0037) | <0.0001 | 0.0756 | (0.0034) | <0.0001 |
| *Cardiometabolic Conditions* | | | | | | | | | | | | |
| Diabetes |  |  |  | -0.0434 | (0.0041) | <0.0001 |  |  |  | -0.0409 | (0.0040) | <0.0001 |
| Heart Disease |  |  |  | -0.0524 | (0.0035) | <0.0001 |  |  |  | -0.0519 | (0.0035) | <0.0001 |
| High Blood Pressure |  |  |  | -0.0369 | (0.0024) | <0.0001 |  |  |  | -0.0349 | (0.0024) | <0.0001 |
| High Cholesterol |  |  |  | -0.0195 | (0.0023) | <0.0001 |  |  |  | -0.0198 | (0.0022) | <0.0001 |
| Stroke |  |  |  | -0.0768 | (0.0066) | <0.0001 |  |  |  | -0.0763 | (0.0066) | <0.0001 |
| Obesity |  |  |  | -0.0306 | (0.0023) | <0.0001 |  |  |  | -0.0150 | (0.0036) | <0.0001 |
| *Body Mass Index* | | | | | | | | | | | | |
| BMI (linear, centered at 25) |  |  |  |  |  |  | -0.0028 | (0.0003) | <0.0001 | -0.0003 | (0.0004) | 0.3805 |
| BMI² (quadratic) |  |  |  |  |  |  | -0.0001 | (0.0000) | <0.0001 | -0.0001 | (0.0000) | <0.0001 |

## Table S13. Survey-Weighted Linear Regression of EQ-5D Utility on Cardiometabolic Conditions and Covariates, MEPS 2009

|  | Model 1: Sociodemographic | | | Model 2: Sociodemographic + CMD | | | Model 3: Sociodemographic + BMI (centered at 25) | | | Model 4: Sociodemographic + CMD + BMI (centered at 25) | | |
| --- | --- | --- | --- | --- | --- | --- | --- | --- | --- | --- | --- | --- |
| Variable | Beta | (SE) | p | Beta | (SE) | p | Beta | (SE) | p | Beta | (SE) | p |
| Intercept | 0.8415 | (0.0045) | <0.0001 | 0.8610 | (0.0038) | <0.0001 | 0.8475 | (0.0046) | <0.0001 | 0.8611 | (0.0041) | <0.0001 |
| *Age group (ref: 18-29 years)* | | | | | | | | | | | | |
| 30-39 | -0.0289 | (0.0025) | <0.0001 | -0.0164 | (0.0024) | <0.0001 | -0.0211 | (0.0025) | <0.0001 | -0.0155 | (0.0024) | <0.0001 |
| 40-49 | -0.0566 | (0.0027) | <0.0001 | -0.0314 | (0.0027) | <0.0001 | -0.0471 | (0.0028) | <0.0001 | -0.0308 | (0.0027) | <0.0001 |
| 50-59 | -0.0878 | (0.0027) | <0.0001 | -0.0460 | (0.0028) | <0.0001 | -0.0767 | (0.0029) | <0.0001 | -0.0454 | (0.0029) | <0.0001 |
| 60-69 | -0.1062 | (0.0034) | <0.0001 | -0.0431 | (0.0035) | <0.0001 | -0.0939 | (0.0034) | <0.0001 | -0.0433 | (0.0035) | <0.0001 |
| 70-79 | -0.1222 | (0.0048) | <0.0001 | -0.0443 | (0.0049) | <0.0001 | -0.1149 | (0.0048) | <0.0001 | -0.0458 | (0.0049) | <0.0001 |
| 80+ | -0.1692 | (0.0058) | <0.0001 | -0.0860 | (0.0061) | <0.0001 | -0.1673 | (0.0059) | <0.0001 | -0.0884 | (0.0062) | <0.0001 |
| *Sex (ref: Female)* | | | | | | | | | | | | |
| Male | 0.0163 | (0.0017) | <0.0001 | 0.0199 | (0.0016) | <0.0001 | 0.0173 | (0.0018) | <0.0001 | 0.0192 | (0.0017) | <0.0001 |
| *Race/Ethnicity (ref: Non-Hispanic White)* | | | | | | | | | | | | |
| Non-Hispanic Black | 0.0026 | (0.0026) | 0.3238 | 0.0097 | (0.0025) | <0.001 | 0.0083 | (0.0025) | 0.0012 | 0.0105 | (0.0025) | <0.0001 |
| Hispanic | 0.0219 | (0.0029) | <0.0001 | 0.0172 | (0.0026) | <0.0001 | 0.0226 | (0.0027) | <0.0001 | 0.0172 | (0.0025) | <0.0001 |
| Non-Hispanic Asian/Pacific Islander | 0.0193 | (0.0033) | <0.0001 | 0.0096 | (0.0032) | 0.0035 | 0.0090 | (0.0034) | 0.0088 | 0.0085 | (0.0033) | 0.0097 |
| Non-Hispanic Other | -0.0108 | (0.0074) | 0.1460 | -0.0017 | (0.0063) | 0.7885 | -0.0079 | (0.0070) | 0.2606 | -0.0014 | (0.0063) | 0.8260 |
| *Education (ref: No degree)* | | | | | | | | | | | | |
| High school diploma / GED | 0.0207 | (0.0029) | <0.0001 | 0.0168 | (0.0026) | <0.0001 | 0.0206 | (0.0029) | <0.0001 | 0.0171 | (0.0026) | <0.0001 |
| Other post-secondary degree | 0.0304 | (0.0039) | <0.0001 | 0.0260 | (0.0036) | <0.0001 | 0.0307 | (0.0037) | <0.0001 | 0.0262 | (0.0036) | <0.0001 |
| Bachelor's degree | 0.0517 | (0.0036) | <0.0001 | 0.0418 | (0.0032) | <0.0001 | 0.0470 | (0.0035) | <0.0001 | 0.0417 | (0.0032) | <0.0001 |
| Master's or Doctorate degree | 0.0638 | (0.0041) | <0.0001 | 0.0490 | (0.0038) | <0.0001 | 0.0575 | (0.0040) | <0.0001 | 0.0491 | (0.0038) | <0.0001 |
| *Income (% poverty level, ref: Poor <100%)* | | | | | | | | | | | | |
| Near poor (100%-124%) | 0.0104 | (0.0056) | 0.0653 | 0.0125 | (0.0052) | 0.0160 | 0.0118 | (0.0055) | 0.0329 | 0.0128 | (0.0051) | 0.0135 |
| Low income (125%-199%) | 0.0328 | (0.0041) | <0.0001 | 0.0310 | (0.0038) | <0.0001 | 0.0330 | (0.0041) | <0.0001 | 0.0308 | (0.0038) | <0.0001 |
| Middle income (200%-399%) | 0.0606 | (0.0035) | <0.0001 | 0.0548 | (0.0033) | <0.0001 | 0.0595 | (0.0035) | <0.0001 | 0.0543 | (0.0033) | <0.0001 |
| High income (≥400%) | 0.0789 | (0.0037) | <0.0001 | 0.0715 | (0.0035) | <0.0001 | 0.0770 | (0.0037) | <0.0001 | 0.0706 | (0.0035) | <0.0001 |
| *Cardiometabolic Conditions* | | | | | | | | | | | | |
| Diabetes |  |  |  | -0.0393 | (0.0041) | <0.0001 |  |  |  | -0.0372 | (0.0040) | <0.0001 |
| Heart Disease |  |  |  | -0.0529 | (0.0032) | <0.0001 |  |  |  | -0.0522 | (0.0032) | <0.0001 |
| High Blood Pressure |  |  |  | -0.0397 | (0.0021) | <0.0001 |  |  |  | -0.0378 | (0.0021) | <0.0001 |
| High Cholesterol |  |  |  | -0.0216 | (0.0022) | <0.0001 |  |  |  | -0.0215 | (0.0023) | <0.0001 |
| Stroke |  |  |  | -0.0734 | (0.0066) | <0.0001 |  |  |  | -0.0738 | (0.0066) | <0.0001 |
| Obesity |  |  |  | -0.0286 | (0.0019) | <0.0001 |  |  |  | -0.0117 | (0.0027) | <0.0001 |
| *Body Mass Index* | | | | | | | | | | | | |
| BMI (linear, centered at 25) |  |  |  |  |  |  | -0.0032 | (0.0004) | <0.0001 | -0.0009 | (0.0005) | 0.0572 |
| BMI² (quadratic) |  |  |  |  |  |  | -0.0000 | (0.0000) | 0.1124 | -0.0001 | (0.0000) | 0.0354 |

## Table S14. Survey-Weighted Linear Regression of EQ-5D Utility on Cardiometabolic Conditions and Covariates, MEPS 2008

|  | Model 1: Sociodemographic | | | Model 2: Sociodemographic + CMD | | | Model 3: Sociodemographic + BMI (centered at 25) | | | Model 4: Sociodemographic + CMD + BMI (centered at 25) | | |
| --- | --- | --- | --- | --- | --- | --- | --- | --- | --- | --- | --- | --- |
| Variable | Beta | (SE) | p | Beta | (SE) | p | Beta | (SE) | p | Beta | (SE) | p |
| Intercept | 0.8413 | (0.0050) | <0.0001 | 0.8624 | (0.0046) | <0.0001 | 0.8506 | (0.0050) | <0.0001 | 0.8647 | (0.0047) | <0.0001 |
| *Age group (ref: 18-29 years)* | | | | | | | | | | | | |
| 30-39 | -0.0295 | (0.0023) | <0.0001 | -0.0155 | (0.0022) | <0.0001 | -0.0216 | (0.0024) | <0.0001 | -0.0153 | (0.0022) | <0.0001 |
| 40-49 | -0.0579 | (0.0024) | <0.0001 | -0.0301 | (0.0023) | <0.0001 | -0.0473 | (0.0024) | <0.0001 | -0.0297 | (0.0023) | <0.0001 |
| 50-59 | -0.0873 | (0.0029) | <0.0001 | -0.0431 | (0.0028) | <0.0001 | -0.0751 | (0.0029) | <0.0001 | -0.0432 | (0.0027) | <0.0001 |
| 60-69 | -0.1071 | (0.0039) | <0.0001 | -0.0432 | (0.0036) | <0.0001 | -0.0970 | (0.0038) | <0.0001 | -0.0445 | (0.0036) | <0.0001 |
| 70-79 | -0.1222 | (0.0047) | <0.0001 | -0.0433 | (0.0044) | <0.0001 | -0.1156 | (0.0047) | <0.0001 | -0.0458 | (0.0044) | <0.0001 |
| 80+ | -0.1667 | (0.0069) | <0.0001 | -0.0830 | (0.0062) | <0.0001 | -0.1650 | (0.0067) | <0.0001 | -0.0865 | (0.0062) | <0.0001 |
| *Sex (ref: Female)* | | | | | | | | | | | | |
| Male | 0.0147 | (0.0016) | <0.0001 | 0.0180 | (0.0015) | <0.0001 | 0.0145 | (0.0016) | <0.0001 | 0.0165 | (0.0015) | <0.0001 |
| *Race/Ethnicity (ref: Non-Hispanic White)* | | | | | | | | | | | | |
| Non-Hispanic Black | 0.0027 | (0.0031) | 0.3832 | 0.0102 | (0.0029) | <0.001 | 0.0090 | (0.0031) | 0.0040 | 0.0111 | (0.0030) | <0.001 |
| Hispanic | 0.0254 | (0.0031) | <0.0001 | 0.0213 | (0.0029) | <0.0001 | 0.0253 | (0.0031) | <0.0001 | 0.0204 | (0.0029) | <0.0001 |
| Non-Hispanic Asian/Pacific Islander | 0.0109 | (0.0036) | 0.0026 | 0.0022 | (0.0033) | 0.5047 | 0.0008 | (0.0035) | 0.8187 | 0.0015 | (0.0033) | 0.6534 |
| Non-Hispanic Other | -0.0271 | (0.0086) | 0.0019 | -0.0156 | (0.0065) | 0.0168 | -0.0225 | (0.0080) | 0.0057 | -0.0139 | (0.0065) | 0.0337 |
| *Education (ref: No degree)* | | | | | | | | | | | | |
| High school diploma / GED | 0.0212 | (0.0032) | <0.0001 | 0.0165 | (0.0030) | <0.0001 | 0.0219 | (0.0033) | <0.0001 | 0.0170 | (0.0030) | <0.0001 |
| Other post-secondary degree | 0.0282 | (0.0044) | <0.0001 | 0.0248 | (0.0041) | <0.0001 | 0.0279 | (0.0044) | <0.0001 | 0.0250 | (0.0041) | <0.0001 |
| Bachelor's degree | 0.0473 | (0.0041) | <0.0001 | 0.0385 | (0.0037) | <0.0001 | 0.0439 | (0.0040) | <0.0001 | 0.0384 | (0.0037) | <0.0001 |
| Master's or Doctorate degree | 0.0602 | (0.0049) | <0.0001 | 0.0472 | (0.0044) | <0.0001 | 0.0557 | (0.0048) | <0.0001 | 0.0476 | (0.0044) | <0.0001 |
| *Income (% poverty level, ref: Poor <100%)* | | | | | | | | | | | | |
| Near poor (100%-124%) | 0.0276 | (0.0073) | <0.001 | 0.0270 | (0.0065) | <0.0001 | 0.0279 | (0.0069) | <0.0001 | 0.0265 | (0.0063) | <0.0001 |
| Low income (125%-199%) | 0.0362 | (0.0051) | <0.0001 | 0.0327 | (0.0048) | <0.0001 | 0.0346 | (0.0050) | <0.0001 | 0.0327 | (0.0047) | <0.0001 |
| Middle income (200%-399%) | 0.0617 | (0.0045) | <0.0001 | 0.0548 | (0.0041) | <0.0001 | 0.0591 | (0.0044) | <0.0001 | 0.0537 | (0.0041) | <0.0001 |
| High income (≥400%) | 0.0833 | (0.0044) | <0.0001 | 0.0717 | (0.0041) | <0.0001 | 0.0773 | (0.0043) | <0.0001 | 0.0698 | (0.0041) | <0.0001 |
| *Cardiometabolic Conditions* | | | | | | | | | | | | |
| Diabetes |  |  |  | -0.0465 | (0.0039) | <0.0001 |  |  |  | -0.0433 | (0.0038) | <0.0001 |
| Heart Disease |  |  |  | -0.0500 | (0.0034) | <0.0001 |  |  |  | -0.0491 | (0.0033) | <0.0001 |
| High Blood Pressure |  |  |  | -0.0399 | (0.0024) | <0.0001 |  |  |  | -0.0376 | (0.0024) | <0.0001 |
| High Cholesterol |  |  |  | -0.0213 | (0.0023) | <0.0001 |  |  |  | -0.0217 | (0.0023) | <0.0001 |
| Stroke |  |  |  | -0.0761 | (0.0059) | <0.0001 |  |  |  | -0.0762 | (0.0059) | <0.0001 |
| Obesity |  |  |  | -0.0300 | (0.0021) | <0.0001 |  |  |  | -0.0117 | (0.0031) | <0.001 |
| *Body Mass Index* | | | | | | | | | | | | |
| BMI (linear, centered at 25) |  |  |  |  |  |  | -0.0025 | (0.0003) | <0.0001 | -0.0004 | (0.0004) | 0.2471 |
| BMI² (quadratic) |  |  |  |  |  |  | -0.0001 | (0.0000) | <0.0001 | -0.0001 | (0.0000) | <0.0001 |

## Table S15. Survey-Weighted Linear Regression of EQ-5D Utility on Cardiometabolic Conditions and Covariates, MEPS 2007

|  | Model 1: Sociodemographic | | | Model 2: Sociodemographic + CMD | | | Model 3: Sociodemographic + BMI (centered at 25) | | | Model 4: Sociodemographic + CMD + BMI (centered at 25) | | |
| --- | --- | --- | --- | --- | --- | --- | --- | --- | --- | --- | --- | --- |
| Variable | Beta | (SE) | p | Beta | (SE) | p | Beta | (SE) | p | Beta | (SE) | p |
| Intercept | 0.8401 | (0.0045) | <0.0001 | 0.8571 | (0.0042) | <0.0001 | 0.8445 | (0.0046) | <0.0001 | 0.8573 | (0.0044) | <0.0001 |
| *Age group (ref: 18-29 years)* | | | | | | | | | | | | |
| 30-39 | -0.0300 | (0.0024) | <0.0001 | -0.0187 | (0.0024) | <0.0001 | -0.0217 | (0.0025) | <0.0001 | -0.0180 | (0.0024) | <0.0001 |
| 40-49 | -0.0556 | (0.0026) | <0.0001 | -0.0340 | (0.0025) | <0.0001 | -0.0456 | (0.0026) | <0.0001 | -0.0333 | (0.0026) | <0.0001 |
| 50-59 | -0.0874 | (0.0030) | <0.0001 | -0.0487 | (0.0031) | <0.0001 | -0.0762 | (0.0032) | <0.0001 | -0.0482 | (0.0032) | <0.0001 |
| 60-69 | -0.1053 | (0.0035) | <0.0001 | -0.0481 | (0.0035) | <0.0001 | -0.0946 | (0.0035) | <0.0001 | -0.0485 | (0.0035) | <0.0001 |
| 70-79 | -0.1300 | (0.0045) | <0.0001 | -0.0586 | (0.0046) | <0.0001 | -0.1239 | (0.0044) | <0.0001 | -0.0599 | (0.0046) | <0.0001 |
| 80+ | -0.1750 | (0.0065) | <0.0001 | -0.0998 | (0.0066) | <0.0001 | -0.1738 | (0.0065) | <0.0001 | -0.1016 | (0.0066) | <0.0001 |
| *Sex (ref: Female)* | | | | | | | | | | | | |
| Male | 0.0156 | (0.0016) | <0.0001 | 0.0178 | (0.0016) | <0.0001 | 0.0172 | (0.0018) | <0.0001 | 0.0176 | (0.0017) | <0.0001 |
| *Race/Ethnicity (ref: Non-Hispanic White)* | | | | | | | | | | | | |
| Non-Hispanic Black | 0.0038 | (0.0029) | 0.1879 | 0.0124 | (0.0027) | <0.0001 | 0.0107 | (0.0029) | <0.001 | 0.0131 | (0.0027) | <0.0001 |
| Hispanic | 0.0189 | (0.0032) | <0.0001 | 0.0168 | (0.0030) | <0.0001 | 0.0214 | (0.0032) | <0.0001 | 0.0166 | (0.0030) | <0.0001 |
| Non-Hispanic Asian/Pacific Islander | 0.0081 | (0.0044) | 0.0672 | -0.0006 | (0.0044) | 0.8948 | -0.0008 | (0.0044) | 0.8482 | -0.0015 | (0.0044) | 0.7399 |
| Non-Hispanic Other | -0.0166 | (0.0086) | 0.0551 | -0.0134 | (0.0082) | 0.1027 | -0.0138 | (0.0086) | 0.1087 | -0.0116 | (0.0081) | 0.1494 |
| *Education (ref: No degree)* | | | | | | | | | | | | |
| High school diploma / GED | 0.0254 | (0.0026) | <0.0001 | 0.0226 | (0.0025) | <0.0001 | 0.0262 | (0.0026) | <0.0001 | 0.0228 | (0.0025) | <0.0001 |
| Other post-secondary degree | 0.0296 | (0.0042) | <0.0001 | 0.0285 | (0.0038) | <0.0001 | 0.0299 | (0.0042) | <0.0001 | 0.0287 | (0.0039) | <0.0001 |
| Bachelor's degree | 0.0483 | (0.0034) | <0.0001 | 0.0410 | (0.0033) | <0.0001 | 0.0444 | (0.0033) | <0.0001 | 0.0403 | (0.0033) | <0.0001 |
| Master's or Doctorate degree | 0.0616 | (0.0038) | <0.0001 | 0.0507 | (0.0036) | <0.0001 | 0.0558 | (0.0037) | <0.0001 | 0.0500 | (0.0036) | <0.0001 |
| *Income (% poverty level, ref: Poor <100%)* | | | | | | | | | | | | |
| Near poor (100%-124%) | 0.0214 | (0.0071) | 0.0025 | 0.0195 | (0.0067) | 0.0039 | 0.0200 | (0.0070) | 0.0045 | 0.0199 | (0.0067) | 0.0032 |
| Low income (125%-199%) | 0.0428 | (0.0046) | <0.0001 | 0.0392 | (0.0043) | <0.0001 | 0.0435 | (0.0044) | <0.0001 | 0.0395 | (0.0043) | <0.0001 |
| Middle income (200%-399%) | 0.0619 | (0.0044) | <0.0001 | 0.0577 | (0.0041) | <0.0001 | 0.0618 | (0.0043) | <0.0001 | 0.0574 | (0.0041) | <0.0001 |
| High income (≥400%) | 0.0848 | (0.0045) | <0.0001 | 0.0768 | (0.0042) | <0.0001 | 0.0816 | (0.0045) | <0.0001 | 0.0759 | (0.0042) | <0.0001 |
| *Cardiometabolic Conditions* | | | | | | | | | | | | |
| Diabetes |  |  |  | -0.0513 | (0.0038) | <0.0001 |  |  |  | -0.0487 | (0.0038) | <0.0001 |
| Heart Disease |  |  |  | -0.0546 | (0.0036) | <0.0001 |  |  |  | -0.0549 | (0.0036) | <0.0001 |
| High Blood Pressure |  |  |  | -0.0334 | (0.0026) | <0.0001 |  |  |  | -0.0318 | (0.0026) | <0.0001 |
| High Cholesterol |  |  |  | -0.0211 | (0.0025) | <0.0001 |  |  |  | -0.0208 | (0.0025) | <0.0001 |
| Stroke |  |  |  | -0.0798 | (0.0067) | <0.0001 |  |  |  | -0.0797 | (0.0067) | <0.0001 |
| Obesity |  |  |  | -0.0321 | (0.0021) | <0.0001 |  |  |  | -0.0170 | (0.0031) | <0.0001 |
| *Body Mass Index* | | | | | | | | | | | | |
| BMI (linear, centered at 25) |  |  |  |  |  |  | -0.0034 | (0.0004) | <0.0001 | -0.0008 | (0.0004) | 0.0471 |
| BMI² (quadratic) |  |  |  |  |  |  | -0.0001 | (0.0000) | 0.1143 | -0.0001 | (0.0000) | 0.0266 |

## Table S16. Survey-Weighted Linear Regression of EQ-5D Utility on Cardiometabolic Conditions and Covariates, MEPS 2006

|  | Model 1: Sociodemographic | | | Model 2: Sociodemographic + CMD | | | Model 3: Sociodemographic + BMI (centered at 25) | | | Model 4: Sociodemographic + CMD + BMI (centered at 25) | | |
| --- | --- | --- | --- | --- | --- | --- | --- | --- | --- | --- | --- | --- |
| Variable | Beta | (SE) | p | Beta | (SE) | p | Beta | (SE) | p | Beta | (SE) | p |
| Intercept | 0.8481 | (0.0044) | <0.0001 | 0.8619 | (0.0042) | <0.0001 | 0.8506 | (0.0043) | <0.0001 | 0.8596 | (0.0041) | <0.0001 |
| *Age group (ref: 18-29 years)* | | | | | | | | | | | | |
| 30-39 | -0.0277 | (0.0024) | <0.0001 | -0.0169 | (0.0022) | <0.0001 | -0.0193 | (0.0024) | <0.0001 | -0.0155 | (0.0023) | <0.0001 |
| 40-49 | -0.0573 | (0.0024) | <0.0001 | -0.0371 | (0.0024) | <0.0001 | -0.0470 | (0.0024) | <0.0001 | -0.0354 | (0.0024) | <0.0001 |
| 50-59 | -0.0900 | (0.0030) | <0.0001 | -0.0518 | (0.0029) | <0.0001 | -0.0777 | (0.0031) | <0.0001 | -0.0507 | (0.0029) | <0.0001 |
| 60-69 | -0.1081 | (0.0036) | <0.0001 | -0.0517 | (0.0037) | <0.0001 | -0.0966 | (0.0035) | <0.0001 | -0.0512 | (0.0037) | <0.0001 |
| 70-79 | -0.1292 | (0.0047) | <0.0001 | -0.0580 | (0.0047) | <0.0001 | -0.1215 | (0.0043) | <0.0001 | -0.0583 | (0.0046) | <0.0001 |
| 80+ | -0.1831 | (0.0065) | <0.0001 | -0.1132 | (0.0064) | <0.0001 | -0.1824 | (0.0064) | <0.0001 | -0.1148 | (0.0064) | <0.0001 |
| *Sex (ref: Female)* | | | | | | | | | | | | |
| Male | 0.0150 | (0.0015) | <0.0001 | 0.0170 | (0.0015) | <0.0001 | 0.0175 | (0.0015) | <0.0001 | 0.0181 | (0.0015) | <0.0001 |
| *Race/Ethnicity (ref: Non-Hispanic White)* | | | | | | | | | | | | |
| Non-Hispanic Black | -0.0040 | (0.0029) | 0.1615 | 0.0056 | (0.0025) | 0.0298 | 0.0023 | (0.0027) | 0.4103 | 0.0066 | (0.0025) | 0.0105 |
| Hispanic | 0.0146 | (0.0031) | <0.0001 | 0.0147 | (0.0028) | <0.0001 | 0.0178 | (0.0031) | <0.0001 | 0.0152 | (0.0028) | <0.0001 |
| Non-Hispanic Asian/Pacific Islander | 0.0155 | (0.0044) | <0.001 | 0.0070 | (0.0041) | 0.0907 | 0.0059 | (0.0046) | 0.1991 | 0.0050 | (0.0042) | 0.2389 |
| Non-Hispanic Other | -0.0301 | (0.0075) | <0.0001 | -0.0232 | (0.0072) | 0.0016 | -0.0265 | (0.0073) | <0.001 | -0.0225 | (0.0072) | 0.0021 |
| *Education (ref: No degree)* | | | | | | | | | | | | |
| High school diploma / GED | 0.0194 | (0.0029) | <0.0001 | 0.0173 | (0.0028) | <0.0001 | 0.0204 | (0.0029) | <0.0001 | 0.0177 | (0.0028) | <0.0001 |
| Other post-secondary degree | 0.0333 | (0.0046) | <0.0001 | 0.0303 | (0.0042) | <0.0001 | 0.0342 | (0.0045) | <0.0001 | 0.0306 | (0.0042) | <0.0001 |
| Bachelor's degree | 0.0454 | (0.0034) | <0.0001 | 0.0368 | (0.0032) | <0.0001 | 0.0415 | (0.0032) | <0.0001 | 0.0364 | (0.0032) | <0.0001 |
| Master's or Doctorate degree | 0.0603 | (0.0041) | <0.0001 | 0.0471 | (0.0039) | <0.0001 | 0.0539 | (0.0041) | <0.0001 | 0.0469 | (0.0039) | <0.0001 |
| *Income (% poverty level, ref: Poor <100%)* | | | | | | | | | | | | |
| Near poor (100%-124%) | 0.0129 | (0.0055) | 0.0193 | 0.0142 | (0.0050) | 0.0046 | 0.0129 | (0.0054) | 0.0169 | 0.0140 | (0.0050) | 0.0053 |
| Low income (125%-199%) | 0.0398 | (0.0045) | <0.0001 | 0.0365 | (0.0042) | <0.0001 | 0.0387 | (0.0044) | <0.0001 | 0.0361 | (0.0042) | <0.0001 |
| Middle income (200%-399%) | 0.0616 | (0.0041) | <0.0001 | 0.0578 | (0.0040) | <0.0001 | 0.0602 | (0.0041) | <0.0001 | 0.0573 | (0.0040) | <0.0001 |
| High income (≥400%) | 0.0805 | (0.0041) | <0.0001 | 0.0751 | (0.0038) | <0.0001 | 0.0772 | (0.0041) | <0.0001 | 0.0741 | (0.0038) | <0.0001 |
| *Cardiometabolic Conditions* | | | | | | | | | | | | |
| Diabetes |  |  |  | -0.0540 | (0.0040) | <0.0001 |  |  |  | -0.0523 | (0.0040) | <0.0001 |
| Heart Disease |  |  |  | -0.0552 | (0.0036) | <0.0001 |  |  |  | -0.0555 | (0.0036) | <0.0001 |
| High Blood Pressure |  |  |  | -0.0381 | (0.0027) | <0.0001 |  |  |  | -0.0362 | (0.0027) | <0.0001 |
| High Cholesterol |  |  |  | -0.0165 | (0.0024) | <0.0001 |  |  |  | -0.0156 | (0.0024) | <0.0001 |
| Stroke |  |  |  | -0.0817 | (0.0066) | <0.0001 |  |  |  | -0.0824 | (0.0066) | <0.0001 |
| Obesity |  |  |  | -0.0318 | (0.0022) | <0.0001 |  |  |  | -0.0136 | (0.0030) | <0.0001 |
| *Body Mass Index* | | | | | | | | | | | | |
| BMI (linear, centered at 25) |  |  |  |  |  |  | -0.0040 | (0.0002) | <0.0001 | -0.0019 | (0.0002) | <0.0001 |
| BMI² (quadratic) |  |  |  |  |  |  | 0.0000 | (0.0000) | <0.001 | 0.0000 | (0.0000) | 0.6175 |

## Table S17. Survey-Weighted Linear Regression of EQ-5D Utility on Cardiometabolic Conditions and Covariates, MEPS 2005

|  | Model 1: Sociodemographic | | | Model 2: Sociodemographic + CMD | | | Model 3: Sociodemographic + BMI (centered at 25) | | | Model 4: Sociodemographic + CMD + BMI (centered at 25) | | |
| --- | --- | --- | --- | --- | --- | --- | --- | --- | --- | --- | --- | --- |
| Variable | Beta | (SE) | p | Beta | (SE) | p | Beta | (SE) | p | Beta | (SE) | p |
| Intercept | 0.8420 | (0.0044) | <0.0001 | 0.8569 | (0.0040) | <0.0001 | 0.8456 | (0.0044) | <0.0001 | 0.8567 | (0.0041) | <0.0001 |
| *Age group (ref: 18-29 years)* | | | | | | | | | | | | |
| 30-39 | -0.0318 | (0.0025) | <0.0001 | -0.0217 | (0.0023) | <0.0001 | -0.0245 | (0.0025) | <0.0001 | -0.0209 | (0.0023) | <0.0001 |
| 40-49 | -0.0599 | (0.0025) | <0.0001 | -0.0400 | (0.0025) | <0.0001 | -0.0502 | (0.0026) | <0.0001 | -0.0390 | (0.0025) | <0.0001 |
| 50-59 | -0.0922 | (0.0029) | <0.0001 | -0.0561 | (0.0028) | <0.0001 | -0.0804 | (0.0030) | <0.0001 | -0.0553 | (0.0028) | <0.0001 |
| 60-69 | -0.1085 | (0.0037) | <0.0001 | -0.0550 | (0.0035) | <0.0001 | -0.0977 | (0.0037) | <0.0001 | -0.0550 | (0.0035) | <0.0001 |
| 70-79 | -0.1313 | (0.0041) | <0.0001 | -0.0630 | (0.0039) | <0.0001 | -0.1247 | (0.0043) | <0.0001 | -0.0642 | (0.0039) | <0.0001 |
| 80+ | -0.1699 | (0.0067) | <0.0001 | -0.1038 | (0.0064) | <0.0001 | -0.1681 | (0.0066) | <0.0001 | -0.1059 | (0.0064) | <0.0001 |
| *Sex (ref: Female)* | | | | | | | | | | | | |
| Male | 0.0166 | (0.0018) | <0.0001 | 0.0182 | (0.0016) | <0.0001 | 0.0188 | (0.0019) | <0.0001 | 0.0185 | (0.0017) | <0.0001 |
| *Race/Ethnicity (ref: Non-Hispanic White)* | | | | | | | | | | | | |
| Non-Hispanic Black | -0.0053 | (0.0031) | 0.0881 | 0.0014 | (0.0028) | 0.6154 | 0.0012 | (0.0030) | 0.6789 | 0.0024 | (0.0028) | 0.3772 |
| Hispanic | 0.0150 | (0.0028) | <0.0001 | 0.0127 | (0.0027) | <0.0001 | 0.0170 | (0.0027) | <0.0001 | 0.0128 | (0.0027) | <0.0001 |
| Non-Hispanic Asian/Pacific Islander | 0.0142 | (0.0044) | 0.0016 | 0.0044 | (0.0043) | 0.3026 | 0.0047 | (0.0046) | 0.3046 | 0.0033 | (0.0043) | 0.4468 |
| Non-Hispanic Other | -0.0371 | (0.0080) | <0.0001 | -0.0224 | (0.0081) | 0.0061 | -0.0298 | (0.0079) | <0.001 | -0.0219 | (0.0081) | 0.0071 |
| *Education (ref: No degree)* | | | | | | | | | | | | |
| High school diploma / GED | 0.0192 | (0.0028) | <0.0001 | 0.0180 | (0.0027) | <0.0001 | 0.0199 | (0.0028) | <0.0001 | 0.0180 | (0.0027) | <0.0001 |
| Other post-secondary degree | 0.0326 | (0.0046) | <0.0001 | 0.0304 | (0.0044) | <0.0001 | 0.0328 | (0.0047) | <0.0001 | 0.0306 | (0.0044) | <0.0001 |
| Bachelor's degree | 0.0454 | (0.0033) | <0.0001 | 0.0388 | (0.0033) | <0.0001 | 0.0420 | (0.0033) | <0.0001 | 0.0385 | (0.0033) | <0.0001 |
| Master's or Doctorate degree | 0.0607 | (0.0038) | <0.0001 | 0.0515 | (0.0037) | <0.0001 | 0.0547 | (0.0037) | <0.0001 | 0.0512 | (0.0036) | <0.0001 |
| *Income (% poverty level, ref: Poor <100%)* | | | | | | | | | | | | |
| Near poor (100%-124%) | 0.0313 | (0.0057) | <0.0001 | 0.0288 | (0.0052) | <0.0001 | 0.0296 | (0.0057) | <0.0001 | 0.0289 | (0.0052) | <0.0001 |
| Low income (125%-199%) | 0.0473 | (0.0049) | <0.0001 | 0.0449 | (0.0048) | <0.0001 | 0.0465 | (0.0049) | <0.0001 | 0.0443 | (0.0047) | <0.0001 |
| Middle income (200%-399%) | 0.0644 | (0.0042) | <0.0001 | 0.0603 | (0.0040) | <0.0001 | 0.0633 | (0.0041) | <0.0001 | 0.0596 | (0.0040) | <0.0001 |
| High income (≥400%) | 0.0890 | (0.0046) | <0.0001 | 0.0812 | (0.0044) | <0.0001 | 0.0857 | (0.0045) | <0.0001 | 0.0801 | (0.0043) | <0.0001 |
| *Cardiometabolic Conditions* | | | | | | | | | | | | |
| Diabetes |  |  |  | -0.0540 | (0.0039) | <0.0001 |  |  |  | -0.0524 | (0.0039) | <0.0001 |
| Heart Disease |  |  |  | -0.0617 | (0.0036) | <0.0001 |  |  |  | -0.0613 | (0.0036) | <0.0001 |
| High Blood Pressure |  |  |  | -0.0326 | (0.0025) | <0.0001 |  |  |  | -0.0307 | (0.0026) | <0.0001 |
| High Cholesterol |  |  |  | -0.0147 | (0.0025) | <0.0001 |  |  |  | -0.0143 | (0.0025) | <0.0001 |
| Stroke |  |  |  | -0.0807 | (0.0076) | <0.0001 |  |  |  | -0.0803 | (0.0075) | <0.0001 |
| Obesity |  |  |  | -0.0326 | (0.0020) | <0.0001 |  |  |  | -0.0166 | (0.0029) | <0.0001 |
| *Body Mass Index* | | | | | | | | | | | | |
| BMI (linear, centered at 25) |  |  |  |  |  |  | -0.0035 | (0.0004) | <0.0001 | -0.0011 | (0.0004) | 0.0072 |
| BMI² (quadratic) |  |  |  |  |  |  | -0.0000 | (0.0000) | 0.3479 | -0.0000 | (0.0000) | 0.1337 |

## Table S18. Survey-Weighted Linear Regression of EQ-5D Utility on Cardiometabolic Conditions and Covariates, MEPS 2004

|  | Model 1: Sociodemographic | | | Model 2: Sociodemographic + CMD | | | Model 3: Sociodemographic + BMI (centered at 25) | | | Model 4: Sociodemographic + CMD + BMI (centered at 25) | | |
| --- | --- | --- | --- | --- | --- | --- | --- | --- | --- | --- | --- | --- |
| Variable | Beta | (SE) | p | Beta | (SE) | p | Beta | (SE) | p | Beta | (SE) | p |
| Intercept | 0.8390 | (0.0042) | <0.0001 | 0.8531 | (0.0039) | <0.0001 | 0.8394 | (0.0040) | <0.0001 | 0.8503 | (0.0038) | <0.0001 |
| *Age group (ref: 18-29 years)* | | | | | | | | | | | | |
| 30-39 | -0.0307 | (0.0022) | <0.0001 | -0.0221 | (0.0021) | <0.0001 | -0.0235 | (0.0022) | <0.0001 | -0.0209 | (0.0021) | <0.0001 |
| 40-49 | -0.0581 | (0.0023) | <0.0001 | -0.0413 | (0.0022) | <0.0001 | -0.0483 | (0.0024) | <0.0001 | -0.0398 | (0.0023) | <0.0001 |
| 50-59 | -0.0898 | (0.0027) | <0.0001 | -0.0559 | (0.0028) | <0.0001 | -0.0770 | (0.0027) | <0.0001 | -0.0544 | (0.0028) | <0.0001 |
| 60-69 | -0.0998 | (0.0038) | <0.0001 | -0.0526 | (0.0035) | <0.0001 | -0.0890 | (0.0038) | <0.0001 | -0.0514 | (0.0035) | <0.0001 |
| 70-79 | -0.1279 | (0.0039) | <0.0001 | -0.0644 | (0.0037) | <0.0001 | -0.1194 | (0.0039) | <0.0001 | -0.0643 | (0.0037) | <0.0001 |
| 80+ | -0.1720 | (0.0067) | <0.0001 | -0.1020 | (0.0074) | <0.0001 | -0.1657 | (0.0070) | <0.0001 | -0.1027 | (0.0074) | <0.0001 |
| *Sex (ref: Female)* | | | | | | | | | | | | |
| Male | 0.0154 | (0.0016) | <0.0001 | 0.0183 | (0.0015) | <0.0001 | 0.0182 | (0.0016) | <0.0001 | 0.0193 | (0.0015) | <0.0001 |
| *Race/Ethnicity (ref: Non-Hispanic White)* | | | | | | | | | | | | |
| Non-Hispanic Black | -0.0005 | (0.0033) | 0.8697 | 0.0067 | (0.0031) | 0.0323 | 0.0059 | (0.0033) | 0.0710 | 0.0075 | (0.0031) | 0.0155 |
| Hispanic | 0.0184 | (0.0028) | <0.0001 | 0.0150 | (0.0025) | <0.0001 | 0.0204 | (0.0026) | <0.0001 | 0.0157 | (0.0025) | <0.0001 |
| Non-Hispanic Asian/Pacific Islander | 0.0138 | (0.0046) | 0.0028 | 0.0038 | (0.0044) | 0.3896 | 0.0042 | (0.0045) | 0.3553 | 0.0023 | (0.0044) | 0.6053 |
| Non-Hispanic Other | -0.0409 | (0.0086) | <0.0001 | -0.0298 | (0.0079) | <0.001 | -0.0345 | (0.0082) | <0.0001 | -0.0290 | (0.0078) | <0.001 |
| *Education (ref: No degree)* | | | | | | | | | | | | |
| High school diploma / GED | 0.0215 | (0.0027) | <0.0001 | 0.0192 | (0.0025) | <0.0001 | 0.0226 | (0.0026) | <0.0001 | 0.0197 | (0.0025) | <0.0001 |
| Other post-secondary degree | 0.0319 | (0.0042) | <0.0001 | 0.0296 | (0.0039) | <0.0001 | 0.0331 | (0.0040) | <0.0001 | 0.0301 | (0.0039) | <0.0001 |
| Bachelor's degree | 0.0504 | (0.0031) | <0.0001 | 0.0440 | (0.0029) | <0.0001 | 0.0483 | (0.0029) | <0.0001 | 0.0441 | (0.0029) | <0.0001 |
| Master's or Doctorate degree | 0.0625 | (0.0038) | <0.0001 | 0.0519 | (0.0036) | <0.0001 | 0.0569 | (0.0038) | <0.0001 | 0.0517 | (0.0036) | <0.0001 |
| *Income (% poverty level, ref: Poor <100%)* | | | | | | | | | | | | |
| Near poor (100%-124%) | 0.0328 | (0.0055) | <0.0001 | 0.0323 | (0.0053) | <0.0001 | 0.0315 | (0.0054) | <0.0001 | 0.0323 | (0.0053) | <0.0001 |
| Low income (125%-199%) | 0.0457 | (0.0043) | <0.0001 | 0.0442 | (0.0037) | <0.0001 | 0.0455 | (0.0041) | <0.0001 | 0.0441 | (0.0037) | <0.0001 |
| Middle income (200%-399%) | 0.0639 | (0.0039) | <0.0001 | 0.0601 | (0.0036) | <0.0001 | 0.0638 | (0.0037) | <0.0001 | 0.0600 | (0.0036) | <0.0001 |
| High income (≥400%) | 0.0888 | (0.0041) | <0.0001 | 0.0806 | (0.0037) | <0.0001 | 0.0859 | (0.0039) | <0.0001 | 0.0805 | (0.0037) | <0.0001 |
| *Cardiometabolic Conditions* | | | | | | | | | | | | |
| Diabetes |  |  |  | -0.0501 | (0.0042) | <0.0001 |  |  |  | -0.0484 | (0.0042) | <0.0001 |
| Heart Disease |  |  |  | -0.0665 | (0.0037) | <0.0001 |  |  |  | -0.0664 | (0.0037) | <0.0001 |
| High Blood Pressure |  |  |  | -0.0374 | (0.0024) | <0.0001 |  |  |  | -0.0358 | (0.0025) | <0.0001 |
| High Cholesterol |  |  |  |  |  |  |  |  |  |  |  |  |
| Stroke |  |  |  | -0.0656 | (0.0065) | <0.0001 |  |  |  | -0.0663 | (0.0065) | <0.0001 |
| Obesity |  |  |  | -0.0338 | (0.0021) | <0.0001 |  |  |  | -0.0190 | (0.0031) | <0.0001 |
| *Body Mass Index* | | | | | | | | | | | | |
| BMI (linear, centered at 25) |  |  |  |  |  |  | -0.0039 | (0.0002) | <0.0001 | -0.0015 | (0.0003) | <0.0001 |
| BMI² (quadratic) |  |  |  |  |  |  | 0.0000 | (0.0000) | <0.0001 | 0.0000 | (0.0000) | 0.2886 |

## Table S19. Survey-Weighted Linear Regression of EQ-5D Utility on Cardiometabolic Conditions and Covariates, MEPS 2003

|  | Model 1: Sociodemographic | | | Model 2: Sociodemographic + CMD | | | Model 3: Sociodemographic + BMI (centered at 25) | | | Model 4: Sociodemographic + CMD + BMI (centered at 25) | | |
| --- | --- | --- | --- | --- | --- | --- | --- | --- | --- | --- | --- | --- |
| Variable | Beta | (SE) | p | Beta | (SE) | p | Beta | (SE) | p | Beta | (SE) | p |
| Intercept | 0.8502 | (0.0042) | <0.0001 | 0.8638 | (0.0040) | <0.0001 | 0.8513 | (0.0041) | <0.0001 | 0.8621 | (0.0040) | <0.0001 |
| *Age group (ref: 18-29 years)* | | | | | | | | | | | | |
| 30-39 | -0.0283 | (0.0025) | <0.0001 | -0.0208 | (0.0025) | <0.0001 | -0.0218 | (0.0026) | <0.0001 | -0.0197 | (0.0025) | <0.0001 |
| 40-49 | -0.0555 | (0.0023) | <0.0001 | -0.0406 | (0.0022) | <0.0001 | -0.0478 | (0.0023) | <0.0001 | -0.0394 | (0.0022) | <0.0001 |
| 50-59 | -0.0892 | (0.0029) | <0.0001 | -0.0577 | (0.0029) | <0.0001 | -0.0793 | (0.0031) | <0.0001 | -0.0568 | (0.0029) | <0.0001 |
| 60-69 | -0.0955 | (0.0038) | <0.0001 | -0.0522 | (0.0038) | <0.0001 | -0.0869 | (0.0039) | <0.0001 | -0.0514 | (0.0038) | <0.0001 |
| 70-79 | -0.1267 | (0.0043) | <0.0001 | -0.0673 | (0.0042) | <0.0001 | -0.1202 | (0.0043) | <0.0001 | -0.0674 | (0.0042) | <0.0001 |
| 80+ | -0.1680 | (0.0074) | <0.0001 | -0.1052 | (0.0073) | <0.0001 | -0.1660 | (0.0075) | <0.0001 | -0.1065 | (0.0073) | <0.0001 |
| *Sex (ref: Female)* | | | | | | | | | | | | |
| Male | 0.0120 | (0.0017) | <0.0001 | 0.0143 | (0.0016) | <0.0001 | 0.0141 | (0.0017) | <0.0001 | 0.0150 | (0.0016) | <0.0001 |
| *Race/Ethnicity (ref: Non-Hispanic White)* | | | | | | | | | | | | |
| Non-Hispanic Black | -0.0037 | (0.0029) | 0.2071 | 0.0045 | (0.0026) | 0.0823 | 0.0039 | (0.0027) | 0.1612 | 0.0054 | (0.0025) | 0.0365 |
| Hispanic | 0.0181 | (0.0026) | <0.0001 | 0.0143 | (0.0024) | <0.0001 | 0.0207 | (0.0026) | <0.0001 | 0.0150 | (0.0024) | <0.0001 |
| Non-Hispanic Asian/Pacific Islander | 0.0085 | (0.0039) | 0.0295 | 0.0018 | (0.0035) | 0.6039 | 0.0000 | (0.0035) | 0.9996 | 0.0002 | (0.0034) | 0.9433 |
| Non-Hispanic Other | -0.0382 | (0.0069) | <0.0001 | -0.0296 | (0.0065) | <0.0001 | -0.0351 | (0.0067) | <0.0001 | -0.0298 | (0.0064) | <0.0001 |
| *Education (ref: No degree)* | | | | | | | | | | | | |
| High school diploma / GED | 0.0210 | (0.0027) | <0.0001 | 0.0183 | (0.0027) | <0.0001 | 0.0222 | (0.0027) | <0.0001 | 0.0185 | (0.0027) | <0.0001 |
| Other post-secondary degree | 0.0236 | (0.0043) | <0.0001 | 0.0208 | (0.0041) | <0.0001 | 0.0245 | (0.0043) | <0.0001 | 0.0211 | (0.0041) | <0.0001 |
| Bachelor's degree | 0.0446 | (0.0036) | <0.0001 | 0.0373 | (0.0034) | <0.0001 | 0.0425 | (0.0035) | <0.0001 | 0.0373 | (0.0034) | <0.0001 |
| Master's or Doctorate degree | 0.0605 | (0.0039) | <0.0001 | 0.0489 | (0.0036) | <0.0001 | 0.0566 | (0.0039) | <0.0001 | 0.0488 | (0.0036) | <0.0001 |
| *Income (% poverty level, ref: Poor <100%)* | | | | | | | | | | | | |
| Near poor (100%-124%) | 0.0154 | (0.0064) | 0.0176 | 0.0168 | (0.0060) | 0.0053 | 0.0163 | (0.0063) | 0.0099 | 0.0166 | (0.0060) | 0.0059 |
| Low income (125%-199%) | 0.0395 | (0.0041) | <0.0001 | 0.0380 | (0.0038) | <0.0001 | 0.0389 | (0.0041) | <0.0001 | 0.0374 | (0.0038) | <0.0001 |
| Middle income (200%-399%) | 0.0597 | (0.0041) | <0.0001 | 0.0571 | (0.0036) | <0.0001 | 0.0594 | (0.0040) | <0.0001 | 0.0568 | (0.0036) | <0.0001 |
| High income (≥400%) | 0.0826 | (0.0043) | <0.0001 | 0.0758 | (0.0037) | <0.0001 | 0.0806 | (0.0042) | <0.0001 | 0.0754 | (0.0037) | <0.0001 |
| *Cardiometabolic Conditions* | | | | | | | | | | | | |
| Diabetes |  |  |  | -0.0547 | (0.0037) | <0.0001 |  |  |  | -0.0532 | (0.0037) | <0.0001 |
| Heart Disease |  |  |  | -0.0634 | (0.0037) | <0.0001 |  |  |  | -0.0629 | (0.0036) | <0.0001 |
| High Blood Pressure |  |  |  | -0.0356 | (0.0025) | <0.0001 |  |  |  | -0.0343 | (0.0025) | <0.0001 |
| High Cholesterol |  |  |  |  |  |  |  |  |  |  |  |  |
| Stroke |  |  |  | -0.0736 | (0.0076) | <0.0001 |  |  |  | -0.0742 | (0.0076) | <0.0001 |
| Obesity |  |  |  | -0.0300 | (0.0020) | <0.0001 |  |  |  | -0.0162 | (0.0034) | <0.0001 |
| *Body Mass Index* | | | | | | | | | | | | |
| BMI (linear, centered at 25) |  |  |  |  |  |  | -0.0036 | (0.0002) | <0.0001 | -0.0014 | (0.0003) | <0.0001 |
| BMI² (quadratic) |  |  |  |  |  |  | 0.0000 | (0.0000) | 0.0315 | -0.0000 | (0.0000) | 0.5213 |

## Table S20. Survey-Weighted Linear Regression of EQ-5D Utility on Cardiometabolic Conditions and Covariates, MEPS 2002

|  | Model 1: Sociodemographic | | | Model 2: Sociodemographic + CMD | | | Model 3: Sociodemographic + BMI (centered at 25) | | | Model 4: Sociodemographic + CMD + BMI (centered at 25) | | |
| --- | --- | --- | --- | --- | --- | --- | --- | --- | --- | --- | --- | --- |
| Variable | Beta | (SE) | p | Beta | (SE) | p | Beta | (SE) | p | Beta | (SE) | p |
| Intercept | 0.8391 | (0.0046) | <0.0001 | 0.8508 | (0.0044) | <0.0001 | 0.8386 | (0.0046) | <0.0001 | 0.8482 | (0.0045) | <0.0001 |
| *Age group (ref: 18-29 years)* | | | | | | | | | | | | |
| 30-39 | -0.0275 | (0.0023) | <0.0001 | -0.0199 | (0.0025) | <0.0001 | -0.0206 | (0.0025) | <0.0001 | -0.0184 | (0.0025) | <0.0001 |
| 40-49 | -0.0558 | (0.0026) | <0.0001 | -0.0399 | (0.0026) | <0.0001 | -0.0476 | (0.0027) | <0.0001 | -0.0384 | (0.0026) | <0.0001 |
| 50-59 | -0.0851 | (0.0028) | <0.0001 | -0.0547 | (0.0026) | <0.0001 | -0.0755 | (0.0029) | <0.0001 | -0.0534 | (0.0027) | <0.0001 |
| 60-69 | -0.0962 | (0.0034) | <0.0001 | -0.0525 | (0.0031) | <0.0001 | -0.0866 | (0.0035) | <0.0001 | -0.0512 | (0.0032) | <0.0001 |
| 70-79 | -0.1262 | (0.0040) | <0.0001 | -0.0654 | (0.0043) | <0.0001 | -0.1183 | (0.0039) | <0.0001 | -0.0650 | (0.0042) | <0.0001 |
| 80+ | -0.1642 | (0.0056) | <0.0001 | -0.1008 | (0.0058) | <0.0001 | -0.1611 | (0.0057) | <0.0001 | -0.1016 | (0.0058) | <0.0001 |
| *Sex (ref: Female)* | | | | | | | | | | | | |
| Male | 0.0159 | (0.0016) | <0.0001 | 0.0182 | (0.0015) | <0.0001 | 0.0183 | (0.0016) | <0.0001 | 0.0190 | (0.0015) | <0.0001 |
| *Race/Ethnicity (ref: Non-Hispanic White)* | | | | | | | | | | | | |
| Non-Hispanic Black | -0.0029 | (0.0029) | 0.3143 | 0.0061 | (0.0027) | 0.0242 | 0.0040 | (0.0030) | 0.1776 | 0.0075 | (0.0027) | 0.0057 |
| Hispanic | 0.0186 | (0.0027) | <0.0001 | 0.0159 | (0.0024) | <0.0001 | 0.0206 | (0.0027) | <0.0001 | 0.0166 | (0.0024) | <0.0001 |
| Non-Hispanic Asian/Pacific Islander | 0.0050 | (0.0050) | 0.3225 | -0.0013 | (0.0050) | 0.7909 | -0.0049 | (0.0051) | 0.3459 | -0.0031 | (0.0050) | 0.5340 |
| Non-Hispanic Other | -0.0348 | (0.0066) | <0.0001 | -0.0250 | (0.0063) | <0.001 | -0.0310 | (0.0065) | <0.0001 | -0.0247 | (0.0063) | <0.001 |
| *Education (ref: No degree)* | | | | | | | | | | | | |
| High school diploma / GED | 0.0200 | (0.0024) | <0.0001 | 0.0186 | (0.0023) | <0.0001 | 0.0215 | (0.0024) | <0.0001 | 0.0190 | (0.0023) | <0.0001 |
| Other post-secondary degree | 0.0307 | (0.0037) | <0.0001 | 0.0291 | (0.0036) | <0.0001 | 0.0329 | (0.0037) | <0.0001 | 0.0297 | (0.0035) | <0.0001 |
| Bachelor's degree | 0.0423 | (0.0031) | <0.0001 | 0.0366 | (0.0029) | <0.0001 | 0.0408 | (0.0031) | <0.0001 | 0.0367 | (0.0029) | <0.0001 |
| Master's or Doctorate degree | 0.0581 | (0.0037) | <0.0001 | 0.0493 | (0.0034) | <0.0001 | 0.0558 | (0.0035) | <0.0001 | 0.0493 | (0.0034) | <0.0001 |
| *Income (% poverty level, ref: Poor <100%)* | | | | | | | | | | | | |
| Near poor (100%-124%) | 0.0224 | (0.0072) | 0.0020 | 0.0228 | (0.0071) | 0.0014 | 0.0217 | (0.0073) | 0.0031 | 0.0228 | (0.0071) | 0.0014 |
| Low income (125%-199%) | 0.0469 | (0.0041) | <0.0001 | 0.0452 | (0.0038) | <0.0001 | 0.0467 | (0.0041) | <0.0001 | 0.0451 | (0.0038) | <0.0001 |
| Middle income (200%-399%) | 0.0663 | (0.0042) | <0.0001 | 0.0624 | (0.0039) | <0.0001 | 0.0663 | (0.0042) | <0.0001 | 0.0625 | (0.0039) | <0.0001 |
| High income (≥400%) | 0.0855 | (0.0045) | <0.0001 | 0.0789 | (0.0041) | <0.0001 | 0.0837 | (0.0045) | <0.0001 | 0.0786 | (0.0041) | <0.0001 |
| *Cardiometabolic Conditions* | | | | | | | | | | | | |
| Diabetes |  |  |  | -0.0557 | (0.0035) | <0.0001 |  |  |  | -0.0543 | (0.0035) | <0.0001 |
| Heart Disease |  |  |  | -0.0606 | (0.0034) | <0.0001 |  |  |  | -0.0605 | (0.0034) | <0.0001 |
| High Blood Pressure |  |  |  | -0.0342 | (0.0022) | <0.0001 |  |  |  | -0.0328 | (0.0022) | <0.0001 |
| High Cholesterol |  |  |  |  |  |  |  |  |  |  |  |  |
| Stroke |  |  |  | -0.0785 | (0.0062) | <0.0001 |  |  |  | -0.0795 | (0.0062) | <0.0001 |
| Obesity |  |  |  | -0.0297 | (0.0020) | <0.0001 |  |  |  | -0.0149 | (0.0030) | <0.0001 |
| *Body Mass Index* | | | | | | | | | | | | |
| BMI (linear, centered at 25) |  |  |  |  |  |  | -0.0035 | (0.0003) | <0.0001 | -0.0014 | (0.0003) | <0.0001 |
| BMI² (quadratic) |  |  |  |  |  |  | 0.0000 | (0.0000) | 0.5437 | -0.0000 | (0.0000) | 0.7881 |

## Table S21. Survey-Weighted Linear Regression of EQ-5D Utility on Cardiometabolic Conditions and Covariates, MEPS 2001

|  | Model 1: Sociodemographic | | | Model 2: Sociodemographic + CMD | | | Model 3: Sociodemographic + BMI (centered at 25) | | | Model 4: Sociodemographic + CMD + BMI (centered at 25) | | |
| --- | --- | --- | --- | --- | --- | --- | --- | --- | --- | --- | --- | --- |
| Variable | Beta | (SE) | p | Beta | (SE) | p | Beta | (SE) | p | Beta | (SE) | p |
| Intercept | 0.8383 | (0.0047) | <0.0001 | 0.8529 | (0.0043) | <0.0001 | 0.8446 | (0.0048) | <0.0001 | 0.8550 | (0.0044) | <0.0001 |
| *Age group (ref: 18-29 years)* | | | | | | | | | | | | |
| 30-39 | -0.0266 | (0.0025) | <0.0001 | -0.0193 | (0.0025) | <0.0001 | -0.0215 | (0.0026) | <0.0001 | -0.0191 | (0.0026) | <0.0001 |
| 40-49 | -0.0520 | (0.0026) | <0.0001 | -0.0372 | (0.0026) | <0.0001 | -0.0459 | (0.0027) | <0.0001 | -0.0373 | (0.0026) | <0.0001 |
| 50-59 | -0.0788 | (0.0031) | <0.0001 | -0.0490 | (0.0029) | <0.0001 | -0.0710 | (0.0031) | <0.0001 | -0.0495 | (0.0029) | <0.0001 |
| 60-69 | -0.0919 | (0.0033) | <0.0001 | -0.0483 | (0.0033) | <0.0001 | -0.0847 | (0.0035) | <0.0001 | -0.0495 | (0.0034) | <0.0001 |
| 70-79 | -0.1239 | (0.0040) | <0.0001 | -0.0637 | (0.0042) | <0.0001 | -0.1180 | (0.0041) | <0.0001 | -0.0655 | (0.0042) | <0.0001 |
| 80+ | -0.1588 | (0.0053) | <0.0001 | -0.0995 | (0.0059) | <0.0001 | -0.1566 | (0.0054) | <0.0001 | -0.1012 | (0.0058) | <0.0001 |
| *Sex (ref: Female)* | | | | | | | | | | | | |
| Male | 0.0184 | (0.0016) | <0.0001 | 0.0206 | (0.0014) | <0.0001 | 0.0181 | (0.0016) | <0.0001 | 0.0195 | (0.0015) | <0.0001 |
| *Race/Ethnicity (ref: Non-Hispanic White)* | | | | | | | | | | | | |
| Non-Hispanic Black | 0.0036 | (0.0028) | 0.2107 | 0.0101 | (0.0027) | <0.001 | 0.0090 | (0.0029) | 0.0018 | 0.0111 | (0.0026) | <0.0001 |
| Hispanic | 0.0216 | (0.0028) | <0.0001 | 0.0177 | (0.0027) | <0.0001 | 0.0219 | (0.0029) | <0.0001 | 0.0176 | (0.0027) | <0.0001 |
| Non-Hispanic Asian/Pacific Islander | 0.0001 | (0.0052) | 0.9901 | -0.0073 | (0.0051) | 0.1524 | -0.0074 | (0.0052) | 0.1563 | -0.0073 | (0.0050) | 0.1462 |
| Non-Hispanic Other | -0.0394 | (0.0131) | 0.0029 | -0.0327 | (0.0131) | 0.0130 | -0.0365 | (0.0131) | 0.0056 | -0.0328 | (0.0128) | 0.0111 |
| *Education (ref: No degree)* | | | | | | | | | | | | |
| High school diploma / GED | 0.0221 | (0.0030) | <0.0001 | 0.0190 | (0.0027) | <0.0001 | 0.0229 | (0.0029) | <0.0001 | 0.0193 | (0.0026) | <0.0001 |
| Other post-secondary degree | 0.0351 | (0.0041) | <0.0001 | 0.0315 | (0.0038) | <0.0001 | 0.0358 | (0.0039) | <0.0001 | 0.0317 | (0.0037) | <0.0001 |
| Bachelor's degree | 0.0493 | (0.0034) | <0.0001 | 0.0420 | (0.0031) | <0.0001 | 0.0468 | (0.0033) | <0.0001 | 0.0420 | (0.0030) | <0.0001 |
| Master's or Doctorate degree | 0.0562 | (0.0043) | <0.0001 | 0.0458 | (0.0040) | <0.0001 | 0.0526 | (0.0042) | <0.0001 | 0.0459 | (0.0040) | <0.0001 |
| *Income (% poverty level, ref: Poor <100%)* | | | | | | | | | | | | |
| Near poor (100%-124%) | 0.0337 | (0.0067) | <0.0001 | 0.0325 | (0.0063) | <0.0001 | 0.0326 | (0.0066) | <0.0001 | 0.0318 | (0.0063) | <0.0001 |
| Low income (125%-199%) | 0.0396 | (0.0047) | <0.0001 | 0.0373 | (0.0045) | <0.0001 | 0.0386 | (0.0048) | <0.0001 | 0.0370 | (0.0045) | <0.0001 |
| Middle income (200%-399%) | 0.0603 | (0.0046) | <0.0001 | 0.0555 | (0.0043) | <0.0001 | 0.0585 | (0.0046) | <0.0001 | 0.0547 | (0.0043) | <0.0001 |
| High income (≥400%) | 0.0782 | (0.0047) | <0.0001 | 0.0708 | (0.0044) | <0.0001 | 0.0751 | (0.0047) | <0.0001 | 0.0700 | (0.0044) | <0.0001 |
| *Cardiometabolic Conditions* | | | | | | | | | | | | |
| Diabetes |  |  |  | -0.0500 | (0.0039) | <0.0001 |  |  |  | -0.0481 | (0.0039) | <0.0001 |
| Heart Disease |  |  |  | -0.0611 | (0.0039) | <0.0001 |  |  |  | -0.0607 | (0.0038) | <0.0001 |
| High Blood Pressure |  |  |  | -0.0361 | (0.0024) | <0.0001 |  |  |  | -0.0351 | (0.0024) | <0.0001 |
| High Cholesterol |  |  |  |  |  |  |  |  |  |  |  |  |
| Stroke |  |  |  | -0.0854 | (0.0072) | <0.0001 |  |  |  | -0.0855 | (0.0071) | <0.0001 |
| Obesity |  |  |  | -0.0311 | (0.0020) | <0.0001 |  |  |  | -0.0165 | (0.0029) | <0.0001 |
| *Body Mass Index* | | | | | | | | | | | | |
| BMI (linear, centered at 25) |  |  |  |  |  |  | -0.0019 | (0.0002) | <0.0001 | -0.0002 | (0.0003) | 0.5973 |
| BMI² (quadratic) |  |  |  |  |  |  | -0.0001 | (0.0000) | <0.0001 | -0.0001 | (0.0000) | <0.0001 |

## Table S22. Meta-Regression of Disease-Specific EQ-5D Trends: Linear Specification, MEPS 2001–2022

| Condition | k (years) | β (per year) | (SE) | p | p (adj.) | Q | Q (p-value) | I² (%) | Δ (2022 vs. 2001) | p |
| --- | --- | --- | --- | --- | --- | --- | --- | --- | --- | --- |
| Type 2 Diabetes | 19 | 0.000669 | (0.000151) | <0.001 | <0.001 | 15.64 | 0.549 | 0.0 | 0.0082 | 0.189 |
| Heart Disease | 19 | 0.000718 | (0.000149) | <0.001 | <0.001 | 19.17 | 0.319 | 11.3 | 0.0098 | 0.091 |
| High blood pressure | 19 | 0.000017 | (0.000129) | 0.894 | 0.942 | 27.96 | 0.045 | 39.2 | 0.0031 | 0.458 |
| High Cholesterol | 15 | -0.000387 | (0.000164) | 0.019 | 0.037 | 20.48 | 0.084 | 36.5 | -0.0099 | 0.009 |
| Stroke/CVA | 19 | 0.000456 | (0.000264) | 0.084 | 0.127 | 16.47 | 0.491 | 0.0 | 0.0262 | 0.016 |
| Obesity (BMI ≥ 30) | 19 | 0.000006 | (0.000088) | 0.942 | 0.942 | 19.07 | 0.324 | 10.9 | -0.0062 | 0.076 |

*Survey-weighted OLS estimates of condition-specific EQ-5D decrements were pooled across 19 MEPS survey years (2001–2022) using DerSimonian–Laird random-effects meta-regression with calendar year as the predictor. High Cholesterol was measured in only 15 years (2003–2022; not collected in 2001, 2002, 2007, 2009). β = linear time slope (change in EQ-5D decrement per calendar year). SE = standard error. p (adj.) = Benjamini–Hochberg FDR-adjusted p-value. Q = Cochran's Q heterogeneity statistic. I² = percentage of total variance attributable to between-year heterogeneity. Δ = difference in estimated decrement between 2022 and the earliest available year (2001 for all conditions; 2003 for High Cholesterol). Rightmost p = two-sided test for Δ ≠ 0.*

## Table S23. Meta-Regression of Disease-Specific EQ-5D Trends: Quadratic Specification, MEPS 2001–2022

| Condition | k (years) | β (per year) | (SE) | p | p (adj.) | Q | Q (p-value) | I² (%) | Δ (2022 vs. 2001) | p |
| --- | --- | --- | --- | --- | --- | --- | --- | --- | --- | --- |
| Type 2 Diabetes | 19 | 0.000669 | (0.000151) | <0.001 | <0.001 | 15.64 | 0.549 | 0.0 | 0.0082 | 0.189 |
| Heart Disease | 19 | 0.000718 | (0.000149) | <0.001 | <0.001 | 19.17 | 0.319 | 11.3 | 0.0098 | 0.091 |
| High blood pressure | 19 | 0.000017 | (0.000129) | 0.894 | 0.942 | 27.96 | 0.045 | 39.2 | 0.0031 | 0.458 |
| High Cholesterol | 15 | -0.000387 | (0.000164) | 0.019 | 0.037 | 20.48 | 0.084 | 36.5 | -0.0099 | 0.009 |
| Stroke/CVA | 19 | 0.000456 | (0.000264) | 0.084 | 0.127 | 16.47 | 0.491 | 0.0 | 0.0262 | 0.016 |
| Obesity (BMI ≥ 30) | 19 | 0.000006 | (0.000088) | 0.942 | 0.942 | 19.07 | 0.324 | 10.9 | -0.0062 | 0.076 |

*Survey-weighted OLS estimates of condition-specific EQ-5D decrements were pooled across 19 MEPS survey years (2001–2022) using DerSimonian–Laird random-effects meta-regression with calendar year as the predictor. High Cholesterol was measured in only 15 years (2003–2022; not collected in 2001, 2002, 2007, 2009). β = linear time slope (change in EQ-5D decrement per calendar year). SE = standard error. p (adj.) = Benjamini–Hochberg FDR-adjusted p-value. Q = Cochran's Q heterogeneity statistic. I² = percentage of total variance attributable to between-year heterogeneity. Δ = difference in estimated decrement between 2022 and the earliest available year (2001 for all conditions; 2003 for High Cholesterol). Rightmost p = two-sided test for Δ ≠ 0.*

# **Section III. Sensitivity Analyses**

## Table S24. Survey-Weighted Linear Regression of EQ-5D Utility Including COVID-19 Pandemic Year, MEPS 2015, 2016, 2018, 2020, and 2022

|  | Model 1: Sociodemographic | | | Model 2: Sociodemographic + CMD | | | Model 3: Sociodemographic + BMI | | | Model 4: Sociodemographic + CMD + BMI (Primary) | | |
| --- | --- | --- | --- | --- | --- | --- | --- | --- | --- | --- | --- | --- |
| Variable | β | (SE) | p | β | (SE) | p | β | (SE) | p | β | (SE) | p |
| Intercept | 0.8485 | (0.0026) | <0.001 | 0.8687 | (0.0024) | <0.001 | 0.8530 | (0.0027) | <0.001 | 0.8672 | (0.0025) | <0.001 |
| *Age Group (ref: 18–29 years)* | | | | | | | | | | | | |
| 30–39 years | -0.0252 | (0.0017) | <0.001 | -0.0134 | (0.0014) | <0.001 | -0.0179 | (0.0016) | <0.001 | -0.0123 | (0.0015) | <0.001 |
| 40–49 years | -0.0474 | (0.0018) | <0.001 | -0.0229 | (0.0016) | <0.001 | -0.0375 | (0.0018) | <0.001 | -0.0217 | (0.0016) | <0.001 |
| 50–59 years | -0.0745 | (0.0018) | <0.001 | -0.0356 | (0.0016) | <0.001 | -0.0652 | (0.0019) | <0.001 | -0.0351 | (0.0017) | <0.001 |
| 60–69 years | -0.0970 | (0.0022) | <0.001 | -0.0401 | (0.0019) | <0.001 | -0.0884 | (0.0021) | <0.001 | -0.0403 | (0.0020) | <0.001 |
| 70–79 years | -0.1169 | (0.0024) | <0.001 | -0.0442 | (0.0022) | <0.001 | -0.1104 | (0.0024) | <0.001 | -0.0452 | (0.0022) | <0.001 |
| ≥80 years | -0.1642 | (0.0030) | <0.001 | -0.0861 | (0.0030) | <0.001 | -0.1632 | (0.0029) | <0.001 | -0.0880 | (0.0030) | <0.001 |
| *Sex (ref: Female)* | | | | | | | | | | | | |
| Male | 0.0136 | (0.0009) | <0.001 | 0.0177 | (0.0008) | <0.001 | 0.0134 | (0.0009) | <0.001 | 0.0176 | (0.0008) | <0.001 |
| *Race/Ethnicity (ref: Non-Hispanic White)* | | | | | | | | | | | | |
| Non-Hispanic Black | 0.0071 | (0.0019) | <0.001 | 0.0132 | (0.0017) | <0.001 | 0.0125 | (0.0018) | <0.001 | 0.0139 | (0.0017) | <0.001 |
| Hispanic | 0.0286 | (0.0017) | <0.001 | 0.0241 | (0.0015) | <0.001 | 0.0282 | (0.0016) | <0.001 | 0.0241 | (0.0015) | <0.001 |
| Non-Hispanic Asian/Pacific Islander | 0.0202 | (0.0025) | <0.001 | 0.0109 | (0.0022) | <0.001 | 0.0097 | (0.0023) | <0.001 | 0.0093 | (0.0022) | <0.001 |
| Non-Hispanic Other | -0.0172 | (0.0036) | <0.001 | -0.0105 | (0.0034) | 0.002 | -0.0146 | (0.0036) | <0.001 | -0.0103 | (0.0034) | 0.003 |
| *Education (ref: No degree)* | | | | | | | | | | | | |
| High school diploma / GED | 0.0129 | (0.0018) | <0.001 | 0.0133 | (0.0017) | <0.001 | 0.0163 | (0.0018) | <0.001 | 0.0140 | (0.0017) | <0.001 |
| Other post-secondary degree | 0.0217 | (0.0026) | <0.001 | 0.0217 | (0.0024) | <0.001 | 0.0251 | (0.0026) | <0.001 | 0.0224 | (0.0024) | <0.001 |
| Bachelor's degree | 0.0396 | (0.0020) | <0.001 | 0.0320 | (0.0018) | <0.001 | 0.0392 | (0.0019) | <0.001 | 0.0325 | (0.0018) | <0.001 |
| Master's or Doctorate degree | 0.0524 | (0.0023) | <0.001 | 0.0428 | (0.0020) | <0.001 | 0.0500 | (0.0023) | <0.001 | 0.0429 | (0.0020) | <0.001 |
| *Income (ref: Poor, <100% FPL)* | | | | | | | | | | | | |
| Near poor (100%–124%) | 0.0214 | (0.0033) | <0.001 | 0.0198 | (0.0031) | <0.001 | 0.0216 | (0.0034) | <0.001 | 0.0199 | (0.0031) | <0.001 |
| Low income (125%–199%) | 0.0364 | (0.0024) | <0.001 | 0.0327 | (0.0022) | <0.001 | 0.0368 | (0.0024) | <0.001 | 0.0328 | (0.0022) | <0.001 |
| Middle income (200%–399%) | 0.0597 | (0.0022) | <0.001 | 0.0532 | (0.0021) | <0.001 | 0.0602 | (0.0022) | <0.001 | 0.0531 | (0.0021) | <0.001 |
| High income (≥400%) | 0.0807 | (0.0023) | <0.001 | 0.0694 | (0.0020) | <0.001 | 0.0788 | (0.0022) | <0.001 | 0.0688 | (0.0020) | <0.001 |
| *Cardiometabolic Conditions* | | | | | | | | | | | | |
| Type 2 Diabetes |  |  |  | -0.0423 | (0.0021) | <0.001 |  |  |  | -0.0404 | (0.0021) | <0.001 |
| Heart Disease |  |  |  | -0.0495 | (0.0018) | <0.001 |  |  |  | -0.0493 | (0.0017) | <0.001 |
| High blood pressure |  |  |  | -0.0334 | (0.0014) | <0.001 |  |  |  | -0.0319 | (0.0014) | <0.001 |
| High Cholesterol |  |  |  | -0.0231 | (0.0013) | <0.001 |  |  |  | -0.0228 | (0.0013) | <0.001 |
| Stroke/CVA |  |  |  | -0.0714 | (0.0034) | <0.001 |  |  |  | -0.0718 | (0.0034) | <0.001 |
| Obesity (BMI ≥ 30) |  |  |  | -0.0316 | (0.0011) | <0.001 |  |  |  | -0.0146 | (0.0018) | <0.001 |
| *Body Mass Index* | | | | | | | | | | | | |
| BMI − 25 kg/m² (linear) |  |  |  |  |  |  | -0.0034 | (0.0002) | <0.001 | -0.0014 | (0.0002) | <0.001 |
| (BMI − 25)² (quadratic) |  |  |  |  |  |  | -0.000013 | (0.000018) | 0.463 | -0.000020 | (0.000015) | 0.187 |

*Model 1: sociodemographic covariates only (age group, sex, race/ethnicity, education, income). Model 2: sociodemographic + six cardiometabolic conditions (CMD). Model 3: sociodemographic + BMI centered at 25 kg/m² + (BMI−25)². Model 4: sociodemographic + CMD + BMI + (BMI−25)² (fully adjusted; primary model). Conditions not included in a model are shown as blank. FPL = Federal Poverty Level. β = regression coefficient (adjusted EQ-5D utility difference). SE = standard error.*

## Table S25. Annual High Blood Pressure Associated EQ-5D Decrement Across All Survey Years, MEPS 2001–2022

| Year | BMI-Missing Year | Coefficient (SE) | 95% CI | p-value |
| --- | --- | --- | --- | --- |
| 2001 | No | -0.0392 (0.0025) | -0.0440 to -0.0343 | <0.001 |
| 2002 | No | -0.0382 (0.0022) | -0.0426 to -0.0338 | <0.001 |
| 2003 | No | -0.0393 (0.0024) | -0.0441 to -0.0345 | <0.001 |
| 2004 | No | -0.0413 (0.0023) | -0.0459 to -0.0368 | <0.001 |
| 2005 | No | -0.0372 (0.0025) | -0.0420 to -0.0324 | <0.001 |
| 2006 | No | -0.0426 (0.0027) | -0.0478 to -0.0373 | <0.001 |
| 2007 | No | -0.0384 (0.0026) | -0.0435 to -0.0333 | <0.001 |
| 2008 | No | -0.0455 (0.0023) | -0.0501 to -0.0409 | <0.001 |
| 2009 | No | -0.0445 (0.0021) | -0.0486 to -0.0403 | <0.001 |
| 2010 | No | -0.0418 (0.0025) | -0.0467 to -0.0369 | <0.001 |
| 2011 | No | -0.0438 (0.0025) | -0.0487 to -0.0390 | <0.001 |
| 2012 | No | -0.0465 (0.0034) | -0.0531 to -0.0399 | <0.001 |
| 2013 | No | -0.0407 (0.0025) | -0.0456 to -0.0358 | <0.001 |
| 2014 | No | -0.0379 (0.0026) | -0.0429 to -0.0328 | <0.001 |
| 2015 | No | -0.0386 (0.0023) | -0.0431 to -0.0340 | <0.001 |
| 2016 | No | -0.0405 (0.0025) | -0.0453 to -0.0357 | <0.001 |
| 2017 | Yes | -0.0413 (0.0025) | -0.0461 to -0.0365 | <0.001 |
| 2018 | No | -0.0364 (0.0027) | -0.0417 to -0.0312 | <0.001 |
| 2019 | Yes | -0.0404 (0.0025) | -0.0453 to -0.0356 | <0.001 |
| 2020 | No | -0.0413 (0.0031) | -0.0473 to -0.0353 | <0.001 |
| 2021 | Yes | -0.0336 (0.0026) | -0.0388 to -0.0285 | <0.001 |
| 2022 | No | -0.0366 (0.0034) | -0.0432 to -0.0300 | <0.001 |

*Annual high blood pressure–associated EQ-5D utility decrements were estimated from a reduced version of Model 4 in which BMI (centered at 25 kg/m²), BMI², and obesity were omitted to permit inclusion of the three BMI-missing survey cycles (2017, 2019, 2021). All estimates are survey-weighted using MEPS SAQ person weights and were obtained via svyglm (R survey package). BMI-missing years (Yes) indicate cycles in which MEPS did not administer the BMI module; all other years are primary analytic years (No). Confidence intervals are calculated as coefficient ± 1.96 × SE. SE = standard error; CI = confidence interval.*

## Table S26. Meta-Regression Test for High Blood Pressure Associated EQ-5D Trends in BMI-Missing Cycles

| Term | Coefficient (SE) | 95% CI | p-value |
| --- | --- | --- | --- |
| Intercept (year = 2012) | -0.0405 (0.0007) | -0.0419 to -0.0390 | <0.001 |
| Year (per year) | 0.0000 (0.0001) | -0.0002 to 0.0003 | 0.719 |
| BMI-missing year | -0.0113 (0.0075) | -0.0259 to 0.0034 | 0.131 |
| Year x BMI-missing | 0.0019 (0.0010) | -0.0002 to 0.0039 | 0.077 |

*DerSimonian–Laird random-effects meta-regression (metafor package, R; Viechtbauer, 2010) was fitted to the 22 annual high blood pressure–associated EQ-5D decrement estimates from Table S25. Calendar year was centered at 2012; the intercept therefore represents the estimated high blood pressure decrement in 2012. The Year coefficient represents the average annual linear change in the high blood pressure–associated decrement across all 22 survey years. The BMI-missing year indicator (coded 1 for 2017, 2019, and 2021; 0 otherwise) tests whether high blood pressure–associated decrements were systematically different in BMI-missing cycles relative to primary analytic years. The Year × BMI-missing interaction tests whether the temporal trend slope differed between BMI-missing and primary years. Neither the BMI-missing year indicator (p = 0.131) nor the interaction term (p = 0.077) reached statistical significance at the conventional α = 0.05 threshold; together these results provide no statistically significant evidence that excluding BMI-missing cycles biased the primary trend estimates. Tau² = 0.00000; I² = 25.8%; R² = 21.8%. SE = standard error; CI = confidence interval.*

## Table S27. Full Model Comparison: Survey-Weighted Linear Regression of EQ-5D Utility With and Without Ceiling Observations (EQ-5D = 1.0), Pooled MEPS 2015, 2016, 2018, and 2022 (Model 4, Fully Adjusted)

|  | Primary Analysis  (n = 70,601) | | Excluding Ceiling Obs.  (n = 64,053) | | Change  (n = 6,548) | |
| --- | --- | --- | --- | --- | --- | --- |
| Variable | β | p-value | β | p-value | Δβ | \|%\| |
| Intercept | 0.8671 | <0.001 | 0.8537 | <0.001 | -0.0134 | 1.5% |
| *Age Group (ref: 18–29 years)* | | | | | | |
| 30–39 years | -0.0140 | <0.001 | -0.0107 | <0.001 | +0.0033 | 23.6% |
| 40–49 years | -0.0230 | <0.001 | -0.0189 | <0.001 | +0.0040 | 17.6% |
| 50–59 years | -0.0370 | <0.001 | -0.0318 | <0.001 | +0.0053 | 14.2% |
| 60–69 years | -0.0428 | <0.001 | -0.0375 | <0.001 | +0.0054 | 12.6% |
| 70–79 years | -0.0483 | <0.001 | -0.0421 | <0.001 | +0.0062 | 12.9% |
| ≥80 years | -0.0894 | <0.001 | -0.0825 | <0.001 | +0.0068 | 7.6% |
| *Sex (ref: Female)* | | | | | | |
| Male | 0.0172 | <0.001 | 0.0169 | <0.001 | -0.0002 | 1.2% |
| *Race/Ethnicity (ref: Non-Hispanic White)* | | | | | | |
| Non-Hispanic Black | 0.0150 | <0.001 | 0.0130 | <0.001 | -0.0020 | 13.4% |
| Hispanic | 0.0245 | <0.001 | 0.0207 | <0.001 | -0.0038 | 15.5% |
| Non-Hispanic Asian/Pacific Islander | 0.0078 | <0.001 | 0.0047 | 0.061 | -0.0032 | 40.6% |
| Non-Hispanic Other | -0.0082 | 0.016 | -0.0112 | 0.002 | -0.0030 | 36.6% |
| *Education (ref: No degree)* | | | | | | |
| High school diploma / GED | 0.0147 | <0.001 | 0.0154 | <0.001 | +0.0007 | 4.9% |
| Other post-secondary degree | 0.0207 | <0.001 | 0.0214 | <0.001 | +0.0007 | 3.4% |
| Bachelor's degree | 0.0341 | <0.001 | 0.0353 | <0.001 | +0.0012 | 3.6% |
| Master's or Doctorate degree | 0.0442 | <0.001 | 0.0441 | <0.001 | -0.0000 | 0.1% |
| *Income (ref: Poor, <100% FPL)* | | | | | | |
| Near poor (100%–124%) | 0.0207 | <0.001 | 0.0191 | <0.001 | -0.0016 | 7.9% |
| Low income (125%–199%) | 0.0342 | <0.001 | 0.0332 | <0.001 | -0.0010 | 2.9% |
| Middle income (200%–399%) | 0.0540 | <0.001 | 0.0541 | <0.001 | +0.0001 | 0.2% |
| High income (≥400%) | 0.0703 | <0.001 | 0.0719 | <0.001 | +0.0016 | 2.2% |
| *Cardiometabolic Conditions* | | | | | | |
| Type 2 Diabetes | -0.0411 | <0.001 | -0.0400 | <0.001 | +0.0011 | 2.6% |
| Heart Disease | -0.0500 | <0.001 | -0.0484 | <0.001 | +0.0016 | 3.3% |
| High blood pressure | -0.0313 | <0.001 | -0.0286 | <0.001 | +0.0027 | 8.5% |
| High Cholesterol | -0.0232 | <0.001 | -0.0206 | <0.001 | +0.0027 | 11.4% |
| Stroke/CVA | -0.0710 | <0.001 | -0.0702 | <0.001 | +0.0008 | 1.1% |
| Obesity (BMI ≥ 30) | -0.0143 | <0.001 | -0.0115 | <0.001 | +0.0028 | 19.7% |
| *Body Mass Index* | | | | | | |
| BMI − 25 (linear) | -0.0014 | <0.001 | -0.0014 | <0.001 | +0.0000 | 1.7% |
| (BMI − 25)² (quadratic) | -0.000017 | 0.228 | -0.000016 | 0.254 | +0.000001 | 7.2% |

*Model 4 = survey-weighted multivariable linear regression fully adjusted for age group, sex, race/ethnicity, family income (% federal poverty level), education, six cardiometabolic conditions, and BMI (linear and quadratic, centered at 25 kg/m²). Pooled MEPS 2015, 2016, 2018, and 2022; SAQ person-level weights divided by 4. β = regression coefficient (EQ-5D index units). Δβ = (no-ceiling coefficient) − (primary coefficient); |Δβ| ≥ 0.002 shown in bold. |%| = percentage change in absolute coefficient magnitude. Ceiling observations: predicted EQ-5D utility = 1.0 (n = 6,548; 9.3% of pooled sample). Maximum |Δβ| across all six conditions = 0.0027, well below the EQ-5D MCID (~0.03–0.07). Highlighted rows (pale yellow) = cardiometabolic conditions of primary interest. FPL = federal poverty level.*

## Table S28. Comparison of DerSimonian–Laird and Continuous-Time AR(1) Meta-Regression Estimates for Disease-Specific HRQoL Trends

|  | DL Meta-Regression (Primary) | | CAR AR(1) Meta-Regression | |  |
| --- | --- | --- | --- | --- | --- |
| Condition | β (SE) | p-value | β (SE) | p-value | ρ |
| Type 2 Diabetes | 0.000669 (0.000151) | <0.001 | 0.000622 (0.000228) | 0.006 | 0.848 |
| Heart Disease | 0.000718 (0.000149) | <0.001 | 0.000668 (0.000287) | 0.020 | 0.943 |
| High blood pressure | 0.000017 (0.000129) | 0.894 | 0.000046 (0.000352) | 0.897 | 1.000 |
| Stroke/CVA | 0.000456 (0.000264) | 0.084 | 0.000467 (0.000289) | 0.106 | 0.503 |
| Obesity | 0.000006 (0.000088) | 0.942 | -0.000140 (0.000265) | 0.598 | 1.000 |
| High Cholesterolᵃ | -0.000387 (0.000164) | 0.019 | -0.000386 (0.000174) | 0.027 | 0.290 |

*DL = DerSimonian–Laird random-effects meta-regression (primary analysis). CAR AR(1) = continuous-time autoregressive model of order 1 estimated via rma.mv (struct = "CAR", metafor package v4.8), which applies an exponential decay covariance exp(−|tᵢ−tⱼ|) using actual calendar distances; preferred over discrete AR(1) because three MEPS cycles (2017, 2019, 2021) were excluded, creating unequal time intervals. β = annual change in condition-specific EQ-5D regression coefficient; SE = standard error. ρ = estimated autocorrelation between adjacent survey years. ᵃHigh Cholesterol: analysis begins 2005 (variable unavailable in MEPS prior to 2005).*

## Table S29. Survey-Weighted Linear Regression of EQ-5D Utility Using Self-Reported Versus Corrected BMI, Pooled MEPS 2015, 2016, 2018, and 2022 (Model 4, Fully Adjusted)

|  | Primary Model (Self-Reported BMI) | | | Corrected Model (Measured BMI) | | | Difference | |
| --- | --- | --- | --- | --- | --- | --- | --- | --- |
| Variable | β | (SE) | p | β | (SE) | p | Δβ | % Change |
| Intercept | 0.8671 | (0.0026) | <0.001 | 0.8667 | (0.0027) | <0.001 | -0.0004 | 0.0% |
| *Age Group (ref: 18–29 years)* | | | | | | | | |
| 30–39 years | -0.0140 | (0.0016) | <0.001 | -0.0140 | (0.0016) | <0.001 | > −0.0001 | 0.0% |
| 40–49 years | -0.0230 | (0.0018) | <0.001 | -0.0229 | (0.0018) | <0.001 | < 0.0001 | 0.0% |
| 50–59 years | -0.0370 | (0.0018) | <0.001 | -0.0370 | (0.0018) | <0.001 | < 0.0001 | 0.1% |
| 60–69 years | -0.0428 | (0.0021) | <0.001 | -0.0428 | (0.0021) | <0.001 | < 0.0001 | 0.1% |
| 70–79 years | -0.0483 | (0.0025) | <0.001 | -0.0483 | (0.0025) | <0.001 | < 0.0001 | 0.1% |
| ≥80 years | -0.0894 | (0.0032) | <0.001 | -0.0893 | (0.0032) | <0.001 | < 0.0001 | 0.0% |
| *Sex (ref: Female)* | | | | | | | | |
| Male | 0.0172 | (0.0009) | <0.001 | 0.0171 | (0.0009) | <0.001 | > −0.0001 | 0.1% |
| *Race/Ethnicity (ref: Non-Hispanic White)* | | | | | | | | |
| Non-Hispanic Black | 0.0150 | (0.0017) | <0.001 | 0.0150 | (0.0017) | <0.001 | < 0.0001 | 0.1% |
| Hispanic | 0.0245 | (0.0015) | <0.001 | 0.0245 | (0.0015) | <0.001 | < 0.0001 | 0.1% |
| Non-Hispanic Asian/Pacific Islander | 0.0078 | (0.0022) | <0.001 | 0.0079 | (0.0022) | <0.001 | +0.0001 | 0.7% |
| Non-Hispanic Other | -0.0082 | (0.0034) | 0.016 | -0.0081 | (0.0034) | 0.017 | < 0.0001 | 0.6% |
| *Education (ref: No degree)* | | | | | | | | |
| High school diploma / GED | 0.0147 | (0.0017) | <0.001 | 0.0147 | (0.0017) | <0.001 | < 0.0001 | 0.0% |
| Other post-secondary degree | 0.0207 | (0.0027) | <0.001 | 0.0207 | (0.0027) | <0.001 | > −0.0001 | 0.1% |
| Bachelor's degree | 0.0341 | (0.0019) | <0.001 | 0.0340 | (0.0019) | <0.001 | > −0.0001 | 0.0% |
| Master's or Doctorate degree | 0.0442 | (0.0021) | <0.001 | 0.0441 | (0.0021) | <0.001 | > −0.0001 | 0.0% |
| *Income (ref: Poor, <100% FPL)* | | | | | | | | |
| Near poor (100%–124%) | 0.0207 | (0.0034) | <0.001 | 0.0207 | (0.0034) | <0.001 | > −0.0001 | 0.0% |
| Low income (125%–199%) | 0.0342 | (0.0025) | <0.001 | 0.0343 | (0.0025) | <0.001 | < 0.0001 | 0.1% |
| Middle income (200%–399%) | 0.0540 | (0.0022) | <0.001 | 0.0540 | (0.0022) | <0.001 | < 0.0001 | 0.0% |
| High income (≥400%) | 0.0703 | (0.0022) | <0.001 | 0.0703 | (0.0022) | <0.001 | < 0.0001 | 0.0% |
| *Cardiometabolic Conditions* | | | | | | | | |
| Type 2 Diabetes | -0.0411 | (0.0023) | <0.001 | -0.0411 | (0.0023) | <0.001 | > −0.0001 | 0.0% |
| Heart Disease | -0.0500 | (0.0019) | <0.001 | -0.0500 | (0.0019) | <0.001 | > −0.0001 | 0.0% |
| High blood pressure | -0.0313 | (0.0015) | <0.001 | -0.0313 | (0.0015) | <0.001 | > −0.0001 | 0.0% |
| High Cholesterol | -0.0232 | (0.0014) | <0.001 | -0.0232 | (0.0014) | <0.001 | < 0.0001 | 0.1% |
| Stroke/CVA | -0.0710 | (0.0036) | <0.001 | -0.0710 | (0.0036) | <0.001 | > −0.0001 | 0.0% |
| Obesity (BMI ≥ 30) | -0.0143 | (0.0019) | <0.001 | -0.0141 | (0.0019) | <0.001 | +0.0002 | 1.2% |
| *Body Mass Index* | | | | | | | | |
| BMI − 25 kg/m² (linear) | -0.0014 | (0.0002) | <0.001 | -0.0013 | (0.0002) | <0.001 | +0.0001 | 6.8% |
| (BMI − 25)² (quadratic) | -0.000017 | (0.000014) | 0.228 | -0.000015 | (0.000012) | 0.228 | +0.000003 | 14.7% |

*Primary model uses self-reported BMI from MEPS SAQ. Corrected model applies the Stommel and Schoenborn (2009) equation: corrected BMI = (self-reported BMI − 2.204) / 0.922. β = regression coefficient (EQ-5D index units); SE = standard error. Δβ = corrected β − primary β; % Change = |Δβ / primary β| × 100. Survey-weighted OLS (svyglm, Gaussian family); PSU/strata design preserved; sampling weights divided by 4. FPL = federal poverty level.*
